# Temperature Tunable Optical Properties of Tetraheptylaluminum(III) Porphyrin Toward Molecular Thermometer

**DOI:** 10.1002/cphc.202500087

**Published:** 2025-05-14

**Authors:** Stefan Charon, Niloofar Zarrabi, Jatan K. Sharma, Paul A. Karr, Francis D’Souza, Prashanth K. Poddutoori

**Affiliations:** ^1^ Advanced Materials Center University of Minnesota Duluth 1038 University Drive Duluth MN 55812 USA; ^2^ Department of Chemistry & Biochemistry University of Minnesota Duluth 1038 University Drive Duluth MN 55812 USA; ^3^ Department of Chemistry University of North Texas 1155 Union Circle #305070 Denton TX 76203‐5017 USA; ^4^ Department of Physical Sciences and Mathematics Wayne State College 1111 Main Street Wayne NE 68787 USA

**Keywords:** aggregation, amphiphilic aluminum(III) porphyrin, molecular thermometer, self‐assembly, thermochromism

## Abstract

Amphiphilic aluminum(III) porphyrin (AlC7P) has been designed, and its aggregation‐induced photophysics has been reported. The *meso*‐positions of the aluminum(III) porphyrin are functionalized with a heptyl chain (C7), while the central porphyrin features an axial OH group on the Al center. The hydrophilic (—OH) and hydrophobic (C7) properties combined with the Lewis acid (Al center) and Lewis base (—OH group) functionality to facilitate the formation of aggregation through self‐assembly. Spectroscopic studies indicate that aggregation can be modulated by solvent and temperature; it is more pronounced in long‐chain hydrocarbon solvents, such as hexanes and *n*‐heptane, and is favored at lower temperatures. At low temperature conditions, the aggregated molecules manifest low fluorescence yields, whereas at higher temperatures, the self‐assembly breaks down to exhibit high fluorescence yields. This temperature‐dependent aggregation‐induced photophysical behavior observed in hexanes makes this porphyrin ideal for a luminescent molecular thermometer.

## Graphical abstract

An amphiphilic aluminum(III) porphyrin (AlC7P) has been developed, and its temperature‐dependant aggregation‐induced photophysical properties have been investigated. The observed photophysical changes due to the aggregation in haxanes make AlC7P a suitable candidate for a luminescent molecular thermometer.

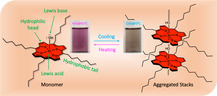

## 1 Introduction

Thermochromism refers to a reversible change in color in response to heating or cooling. This color change is closely linked to the molecular optical properties, such as absorption and emission characteristics. Thermochromism can be established by observing how these optical properties vary with temperature. Consequently, molecules that exhibit thermochromism hold potential as luminescent molecular thermometers (LMTs).^[^ ^1^, ^2^, ^3^, ^4^, ^5^, ^6^, ^7^, ^8^, ^9^, ^10^, ^11^, ^12^, ^13^, ^14^ ^]^ LMTs offer several advantages over traditional thermometers.^[^ ^15^ ^]^ They respond quickly, demonstrate high sensitivity, and provide greater spatial resolution, which many other thermometry techniques cannot achieve. Furthermore, because LMTs can be used nondestructively in areas that are too small for conventional thermometers, they allow for temperature measurement in otherwise impossible situations. One example of using LMTs is in photothermal therapy (PTT).^[^ ^16^, ^17^, ^18^ ^]^ PTT is an anticancer treatment that involves using a laser to heat a tumor to just above 42 °C. This temperature is sufficient to cause the death of cancer cells while keeping the surrounding healthy tissue below harmful temperatures. LMTs provide live feedback on the temperature of both the tumor and the surrounding tissue, enabling the modulation of the laser's power to ensure that only the cancerous cells are affected. Additionally, LMTs are beneficial for the high‐speed and high spatial resolution thermometry feedback required in microfluidic systems.^[^ ^13^ ^]^ In lab‐on‐a‐chip devices, which handle fluids at the nano to picoliter scale conventional thermometers, even the smallest ones, are not adequate for monitoring the entire system simultaneously.^[^ ^1^, ^19^ ^]^ LMTs allow for accurate measurement of the liquid temperature throughout the whole system with both high speed and resolution. These examples highlight the diverse applications of LMTs.

Numerous impressive systems have been reported in the literature for applications in luminescent temperature (LMT) sensing.^[^ ^1^, ^5^, ^17^ ^]^ Several features are essential for an effective temperature probe. Among these, high optical sensitivity toward temperature and good photostability are the most sought‐after characteristics. In this context, various types of molecules have been considered for temperature sensing, including organic dyes, such as pyrene,^[^ ^1^ ^]^ C_70_,^[^ ^9^ ^]^ and BODIPY,^[^ ^6^ ^]^ and inorganic systems, like mixed‐valent uranium (V, VI) organic frameworks,^[^ ^4^ ^]^ and porphyrin derivatives.^[^ ^2^, ^7^, ^10^, ^20^, ^21^, ^22^ ^]^ Porphyrins are well‐known photosensitizers that are characterized by their high fluorescence quantum yields and photostability. Their structural properties can be modified by substituting groups at the peripheral positions or by incorporating different elements into the central cavity.^[^ ^23^, ^24^ ^]^ Due to these versatile characteristics, porphyrins have been extensively used in applications such as artificial photosynthesis,^[^ ^25^, ^26^, ^27^, ^28^, ^29^ ^]^ molecular electronics,^[^ ^30^, ^31^ ^]^ photonics,^[^ ^32^ ^]^ molecular sensors,^[^ ^33^ ^]^ and photodynamic therapy.^[^ ^34^ ^]^ Given their versatility, it is reasonable to consider the potential for thermochromic properties in porphyrin molecules.^[^ ^2^, ^7^, ^10^, ^20^, ^21^, ^22^, ^35^, ^36^ ^]^ In fact, this was explored using the phosphorus(V) porphyrins, π‐extended zinc(II) porphyrins, and platinum(II) octaethylporphyrins for LMT applications.

Aluminum (III) porphyrins are particularly notable among various porphyrins because they feature Lewis acid–base sites.^[^ ^37^, ^38^ ^]^ The aluminum center within the molecule acts as a Lewis acid. This property has been exploited to create multicomponent donor–acceptor systems, which have been documented in the literature for studying photoinduced processes, including energy and electron transfers.^[^ ^39^, ^40^, ^41^, ^42^, ^43^, ^44^, ^45^, ^46^ ^]^ Furthermore, aluminum(III) porphyrins have been used to develop molecular stacks on semiconductor surfaces, 1D polymers, nanorings, and other structures.^[^ ^47^, ^48^, ^49^ ^]^ Despite these applications, the full potential of aluminum(III) porphyrins has not yet been realized. The axial hydroxy group acts as a potential Lewis base (LB) in its pristine form. The coexistence of Lewis acid (LA) and base centers could facilitate self‐assembly; however, this self‐assembly is often unstable enough to influence optical properties for sensor applications. One approach to enhance this stability is to modify the four *meso*‐positions of the porphyrin unit. By doing so, the improved molecular properties could be leveraged for specific applications.

**Scheme 1:**
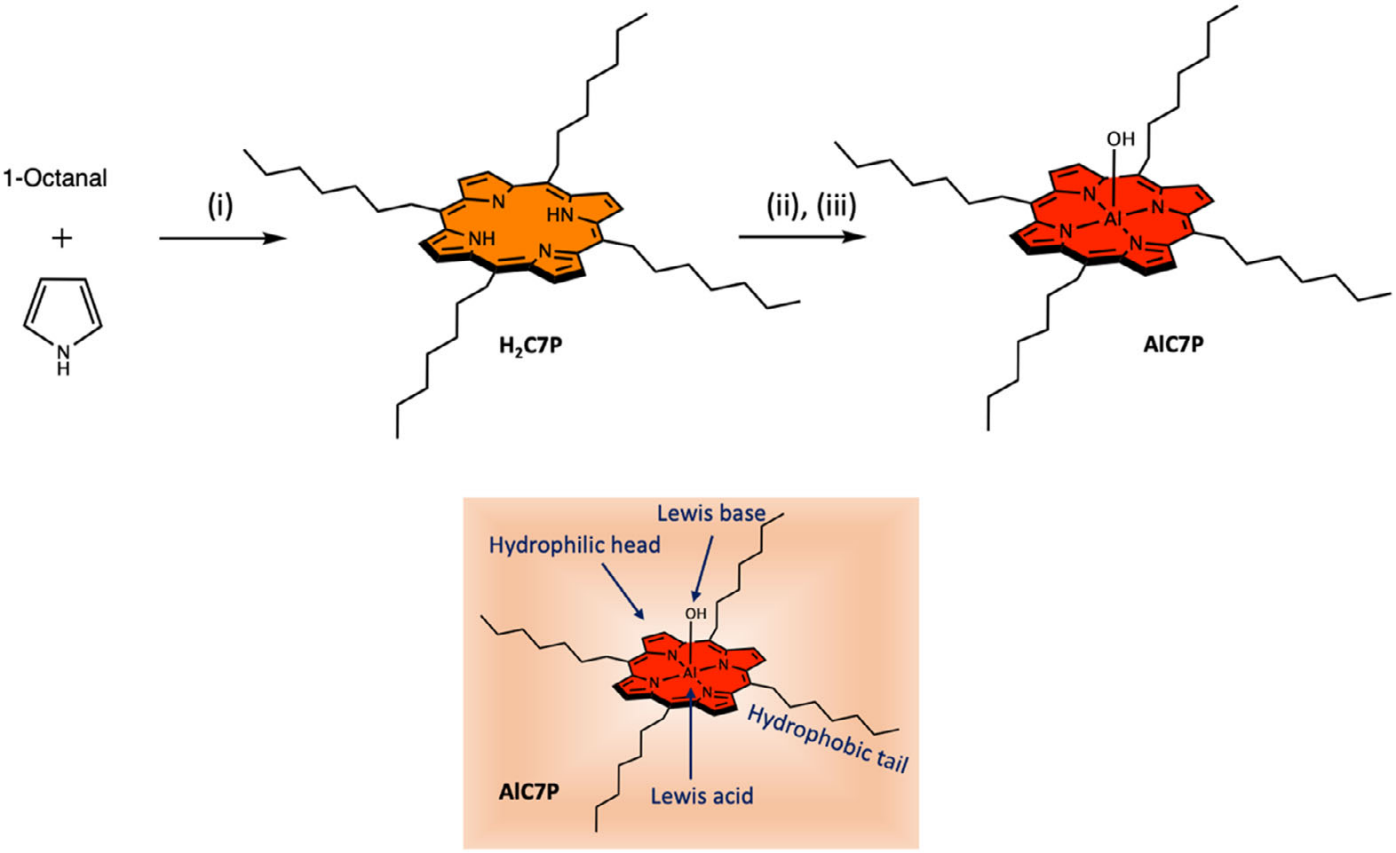
Synthesis of the studied amphiphilic aluminum(III) porphyrin AlC7P. *Reaction conditions*: (i) BF_3_.OEt_2_, CH_2_Cl_2_, stirring under N_2_ for (ii) AlMe_3_, Toluene, and (iii) H_2_O. The inset shows various functionalities in the AlC7P.

To achieve this objective, AlC7P was synthesized with hydrophobic heptyl (C7) units located in the *meso*‐positions (see **Scheme** **1**). The heptyl units serve as nonpolar tails, while the central porphyrin ring, which includes an Al—OH group, acts as a polar head. The axial —OH group also functions as a LB, with the aluminum acting as a LA center. The combination of hydrophilic and hydrophobic components makes this molecule amphiphilic. Our studies indicate that the presence of both nonpolar and polar units contributes to the solubility of AlC7P in nearly all common organic solvents, ranging from nonpolar hexanes to polar solvents like acetonitrile and CH_3_OH. This amphiphilic nature endows the molecule with a unique thermochromic property. Most importantly, both absorption and fluorescence properties are sensitive to the temperature, making the system an ideal candidate for use as a luminescent thermometer.

## 2 Results and Discussion

### 2.1 Synthesis and Characterization

**Figure 1:**
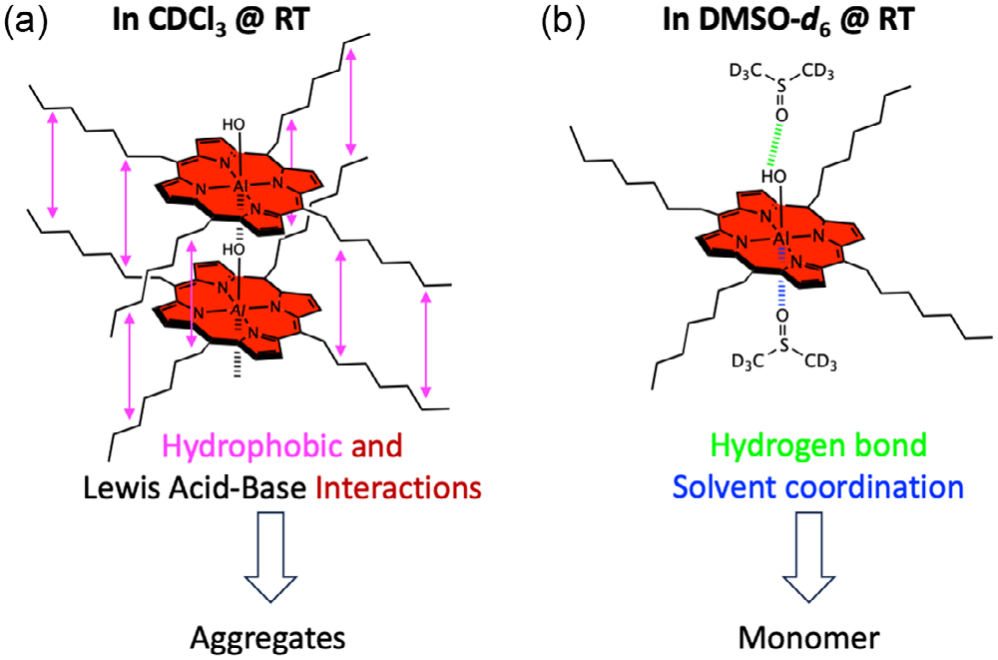
Proposed noncovalent interactions: a) self‐assembly in noncoordinating solvent and b) monomer formation in coordinating solvent of AlC7P at room temperature. Note: The eclipsed type of aggregation stacks and solvent‐AlC7P interactions are predicted based on molecular functionality and spectroscopic observations.

The synthesis details and structural characterization of H_2_C7P and AlC7P are provided in the experimental section. An initial characterization of the compounds was conducted using electrospray ionization (ESI) mass spectrometry, as shown in Figures S1 and S2, Supporting Information. The mass spectra reveal a prominent parent ion peak at 703.5400 for H_2_C7P and 745.5325 for AlC7P, corresponding to the mass (*m/z*) of [M + H]^+^. Additionally, the proton‐nuclear magentic resonance (^1^H NMR) spectra of the studied porphyrins are shown in Figures S3–S5, Supporting Information. In the ^1^H NMR spectrum of H_2_C7P, both shielding and deshielding effects are noticeable for the protons in the molecules, attributed to the ring current effect of the porphyrin macrocycle.^[^ ^50^ ^]^ Similar effects were observed in the ^1^H NMR spectrum of AlC7P in CDCl_3_. However, a careful examination of the spectrum revealed additional sets of peaks for each proton, and the intensity of these peaks increased with the sample concentration, as shown in Figure S4, Supporting Information. These results indicate that AlC7P aggregates through self‐assembly in solution. As shown in **Figure** **1**, this aggregation is driven by two factors: (1) the hydrophobic interactions between the C7 chains and (2) the Lewis acid–base interactions. The C7 units enhance the hydrophobic interactions between molecules, while the LB oxygen atoms form coordination bonds with the LA aluminum center. The *meso*‐heptyl units were chosen to increase the solubility of the AlC7P in nonpolar solvents and to reduce the intermolecular distance between them to enhance the aggregation property. Together, these complementary interactions facilitate the formation of an aggregation stack, as shown in Figure 1a. To confirm the LA‐LB interactions, we measured the ^1^H NMR in DMSO‐*d*_6_, which serves as the LB. The absence of additional sets of peaks indicates that AlC7P predominantly exists in monomer form (Figure S5, Supporting Information). This could be due to the DMSO‐*d*_6_ molecules interfering with self‐assembly by forming hydrogen and coordination bonds (Figure 1b). It is important to note that the structures shown in Figure 1 are the most probable scenarios based on the available functionalities of AlC7P. Also, in Figure 1a, the heptyl chains are depicted in an eclipsed manner to highlight the van der Waal interactions between them, but there is no experimental evidence to confirm it. Additionally, temperature‐dependent ^1^ H NMR studies were performed at 25 and 50 °C, Figure S5, Supporting Information. Upon heating the sample to 50 °C, the NMR revealed a single peak for each type of proton. Overall, the NMR results suggest that aggregation is sensitive to the solvent, concentration, and temperature.

### 2.2 Absorption and Fluorescence Spectroscopy

**Figure 2:**
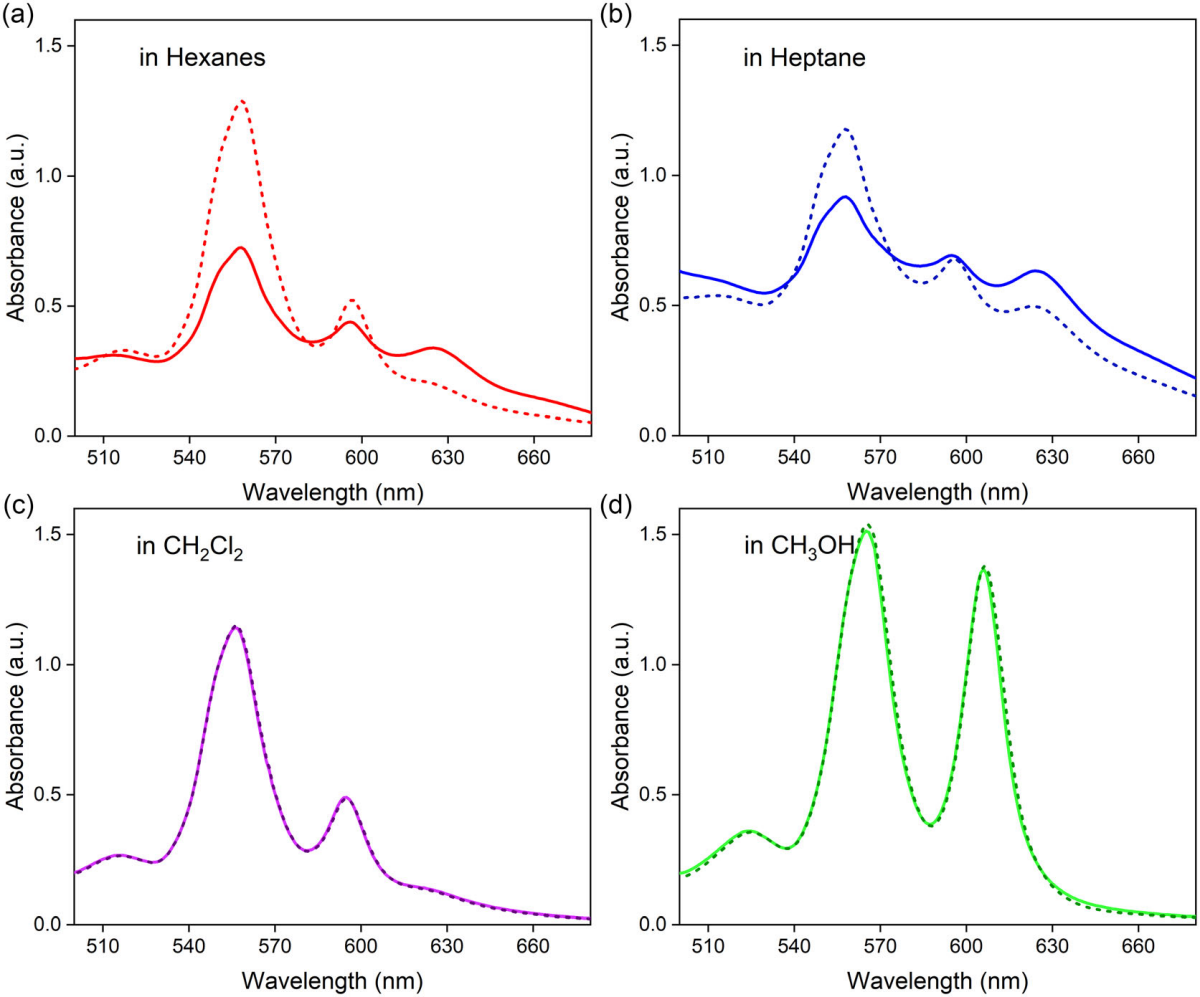
Electronic absorption spectra of AlC7P in different solvents and at two temperatures. The solid line shows the absorbance at room temperature (RT), and the dashed line shows the absorbance at elevated temperatures. a) hexanes (RT to 50 °C), b) *n*‐heptane (RT to 55 °C), c) CH_2_Cl_2_ (RT to 40 °C), and d) CH_3_OH (RT to 50 °C).

Preliminary characterization indicates that AlC7P is prone to aggregation through self‐assembly in noncoordinating solvents. This aggregation can be prevented using coordinating solvents or by increasing the temperature. AlC7P was dissolved in various solvents, including hexane, *n*‐heptane, CH_2_Cl_2_, and CH_3_OH, to investigate the electronic properties during the aggregation process. Absorbance spectra were collected at both room temperature and elevated temperatures. As shown in **Figure** **2**, significant changes were observed in the absorption spectrum of AlC7P when measured in hexanes and *n*‐heptane between room temperature and temperatures of 50–55 °C. At elevated temperatures, the absorption bands at 559 and 596 nm grew more intense, while the absorption band around 630 nm diminished. These spectral changes suggest aggregation occurs at room temperature and disassembles at higher temperatures. Interestingly, these changes were not observed in CH_2_Cl_2_ and CH_3_OH. The absence of changes in CH_3_OH is expected; as a LB, CH_3_OH can coordinate with the LA aluminum center, thereby preventing the formation of aggregates. Consequently, the absorption spectrum remains temperature‐independent under these conditions. However, solvent coordination does shift the spectrum to ≈10 nm longer wavelengths, a phenomenon well‐documented in the literature.^[^ ^51^ ^]^ Additionally, a weak aggregation (evidenced by a weak absorption band around 620 nm) was observed in the noncoordinating solvent CH_2_Cl_2_, likely due to weak LB chloride centers. Overall, the absorption studies suggest that significant aggregation occurs in hexanes and *n*‐heptane, weak aggregation in CH_2_Cl_2_, and no aggregation in CH_3_OH. Therefore, CH_2_Cl_2_ was selected for quantitative absorption and fluorescence studies. Between hexanes and *n*‐heptane, hexanes exhibit more effective thermal properties, leading to the selection of hexanes for further studies on aggregation‐induced spectroscopic properties.

**Figure 3:**
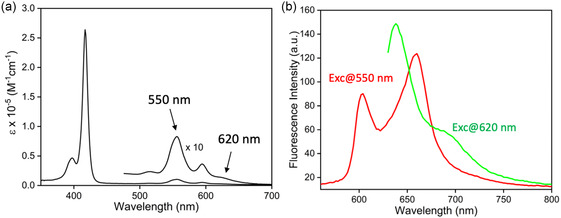
a) Absorption and b) fluorescence spectra (concentration = 1.74 × 10^−5 ^M) of AlC7P in CH_2_Cl_2_ at room temperature, 25 °C.

The quantitative absorption and fluorescence spectra of AlC7P in CH_2_Cl_2_ are shown in **Figure** **3**a. The absorption spectrum exhibits typical porphyrin characteristics, featuring a strong B‐band (Soret band) at 417 nm, along with two weaker Q‐bands at 556 and 594 nm. Notably, there is a weak absorption extending up to 700 nm, which is not typical for tetraphenylaluminum(III) porphyrin (AlTPP)^[^ ^38^ ^]^ or octaethylaluminum(III) porphyrin (AlOEP).^[^ ^52^ ^]^ This suggests that a portion of the sample may still be aggregated. The fluorescence spectra of AlC7P were measured with excitation wavelengths of 550 nm (which predominantly excites the monomer) and 620 nm (which exclusively excites the aggregated form). As shown in Figure 3b, exciting AlC7P at 550 nm produces two emission bands at ≈ 604 and 660 nm, which are characteristic of aluminum(III) porphyrins containing tetraarylporphyrins.^[^ ^38^ ^]^ In contrast, excitation at 620 nm yields a significantly different spectrum, featuring bands at 638 and 690 nm, which are believed to originate from the aggregated form. These results indicate that AlC7P in CH_2_Cl_2_ exists in an equilibrium between monomer and aggregate forms.

### 2.3 Thermochromic Studies

**Figure 4:**
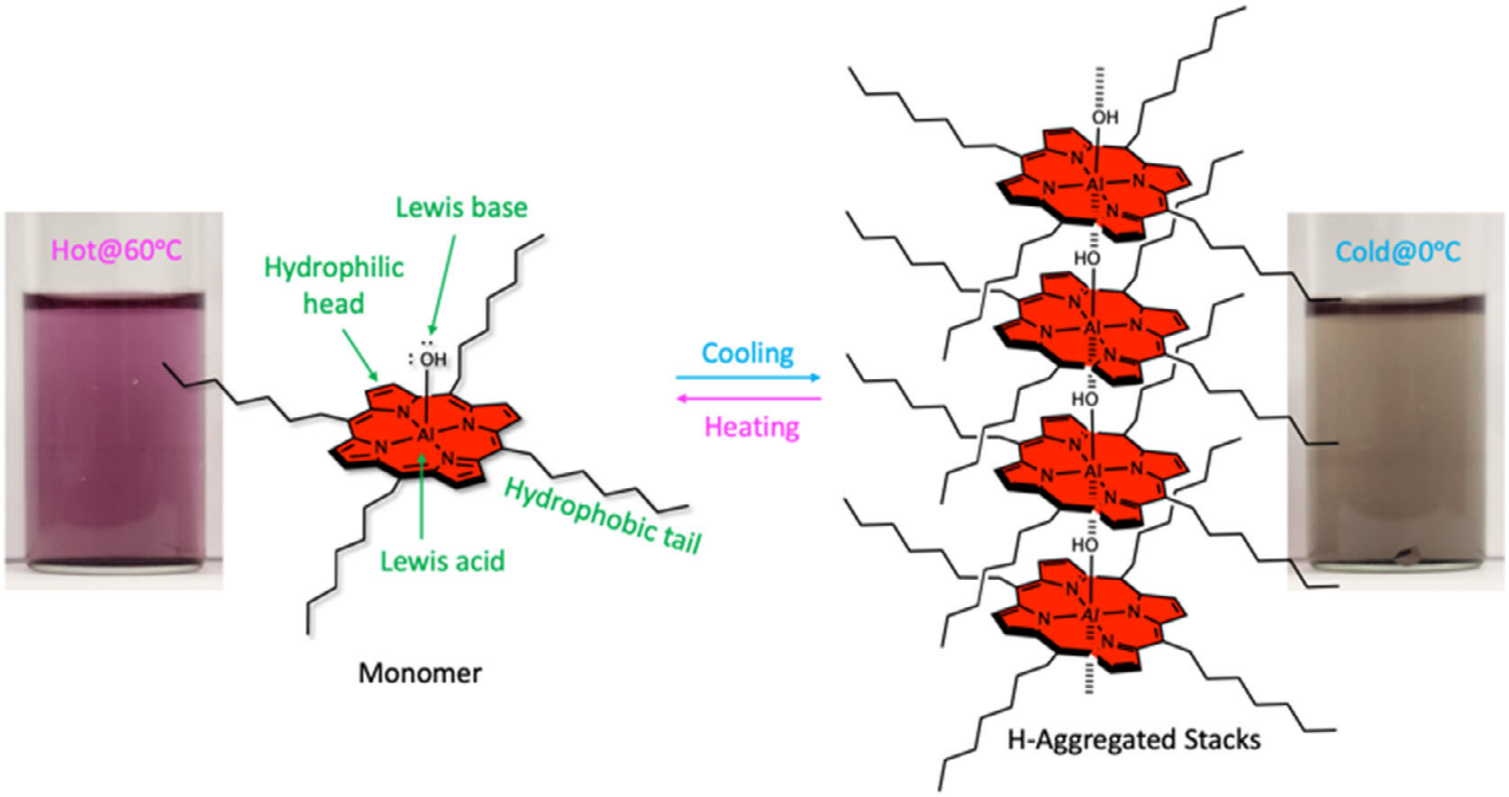
Thermochromic property and the corresponding structural changes of AlC7P at 0 °C ice (right) and at 6 °C (left) in hexanes. Note: The eclipsed type of aggregation is used to improve the clarity of the image.

Aggregation significantly affects both the geometry and the electronic structure of a molecule. These changes can be observed in the optical properties, as aggregated molecules possess a very different electronic structure than their corresponding monomers. Temperature is one of the external factors that can be used to modulate aggregation, and consequently, its optical properties. Observing the color changes of AlC7P in an appropriate solvent as a function of temperature is one of the simplest ways to demonstrate thermochromism. **Figure** **4** shows the thermochromic properties of AlC7P in hexanes. When the solution is cooled to 0 °C, it turns light gray, as shown on the right side of Figure 4. At this temperature, it is expected that AlC7P forms an aggregated state due to hydrophobic interactions and Lewis acid–base interactions. When the temperature is raised to 60 °C, the solution changes to pink, as depicted on the left side of Figure 4. At higher temperatures, the intermolecular interactions between AlC7P break down because the molecules are in a high kinetic state. This results in the formation of monomeric AlC7P, which exhibits the distinctive pink color characteristic of aluminum(III) porphyrin solutions.

**Figure 5:**
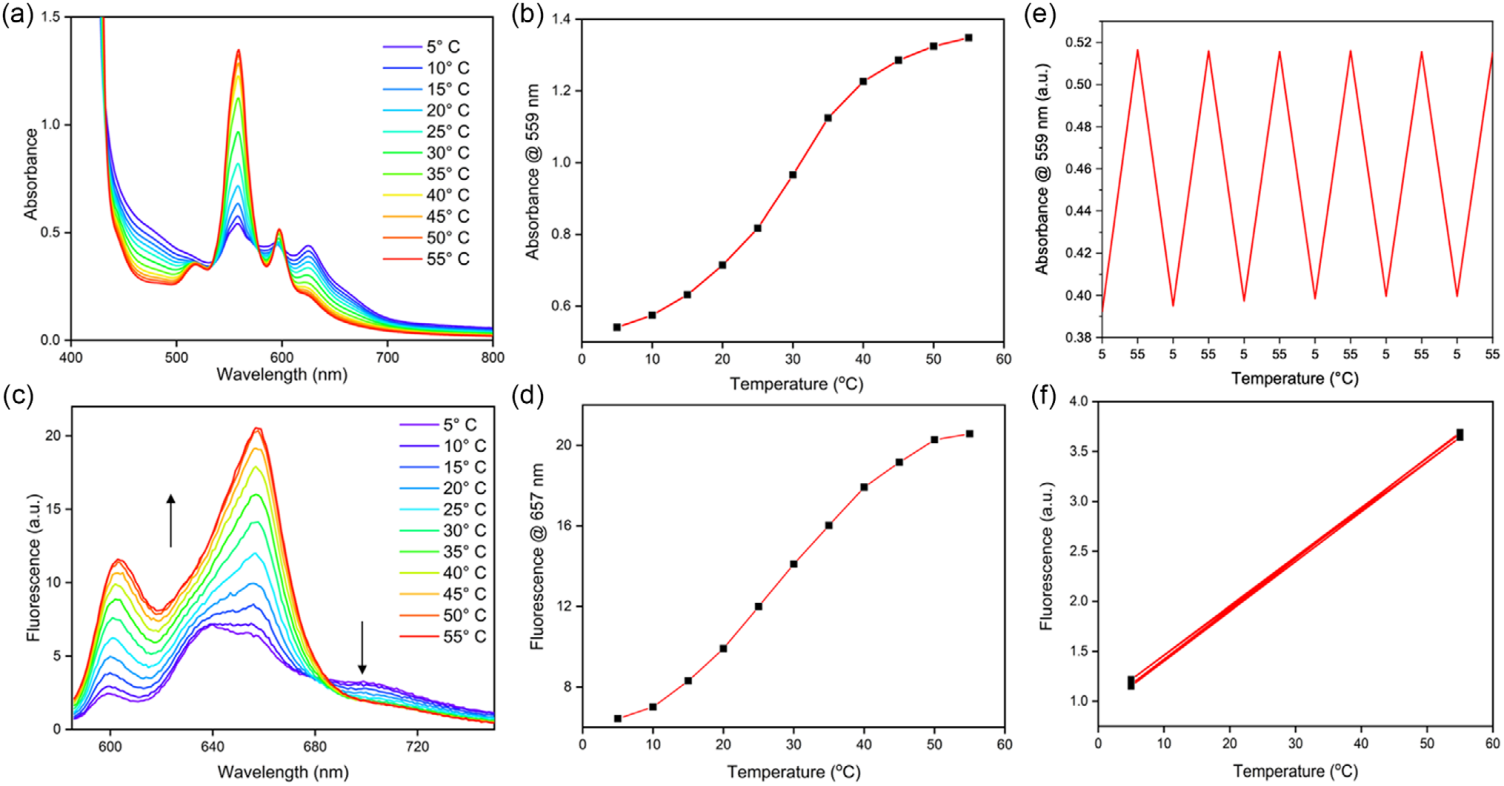
a) T‐dependent absorbance (@Q‐band concentration (1.42 × 10^−4^ M), b) absorbance versus temperature curve at 559 nm, c) T‐dependent fluorescence spectra (concentration = 1.42 × 10^−4^ M), excitation = 576 nm, d) fluorescence intensity versus temperature curve at 657 nm, and e,f) reversibility curves. All the studies were performed in hexanes. During these studies, the solutions were placed in a sealed cuvette to maintain a constant concentration.

A Peltier‐controlled UV–Vis spectrometer was used to collect temperature‐dependent absorbance spectra of AlC7P in hexanes. During the optical studies, the solutions were placed in a sealed cuvette to maintain a constant concentration. This approach prevented the evaporation of the solvent hexanes throughout the experiments. The studies were conducted using Q‐band and Soret (B‐band) concentrations and are shown in **Figures** **5** and S6, Supporting Information, respectively. The thermochromic spectral changes were more pronounced at the Q‐band compared to the B‐band concentration due to increased aggregation at higher concentrations. When the temperature was raised from 5 to 55 °C, the absorbance at 559 nm increased by a factor of 2.6, while the absorbance at 625 nm decreased by a factor of 2.1 (Figure 5a). These curves’ sigmoidal shape (Figure 5b) indicates a transition from an aggregated to a nonaggregated form within this temperature range. To assess the reversibility of the thermochromic properties of AlC7P, the absorbance at 559 nm (Q‐band) was measured while cycling the temperature between 5 and 55 °C six times, as shown in the right section of Figure 5e. Notably, there was no discernible decrease in the thermochromic properties over the number of cycles performed.

**Figure 6:**
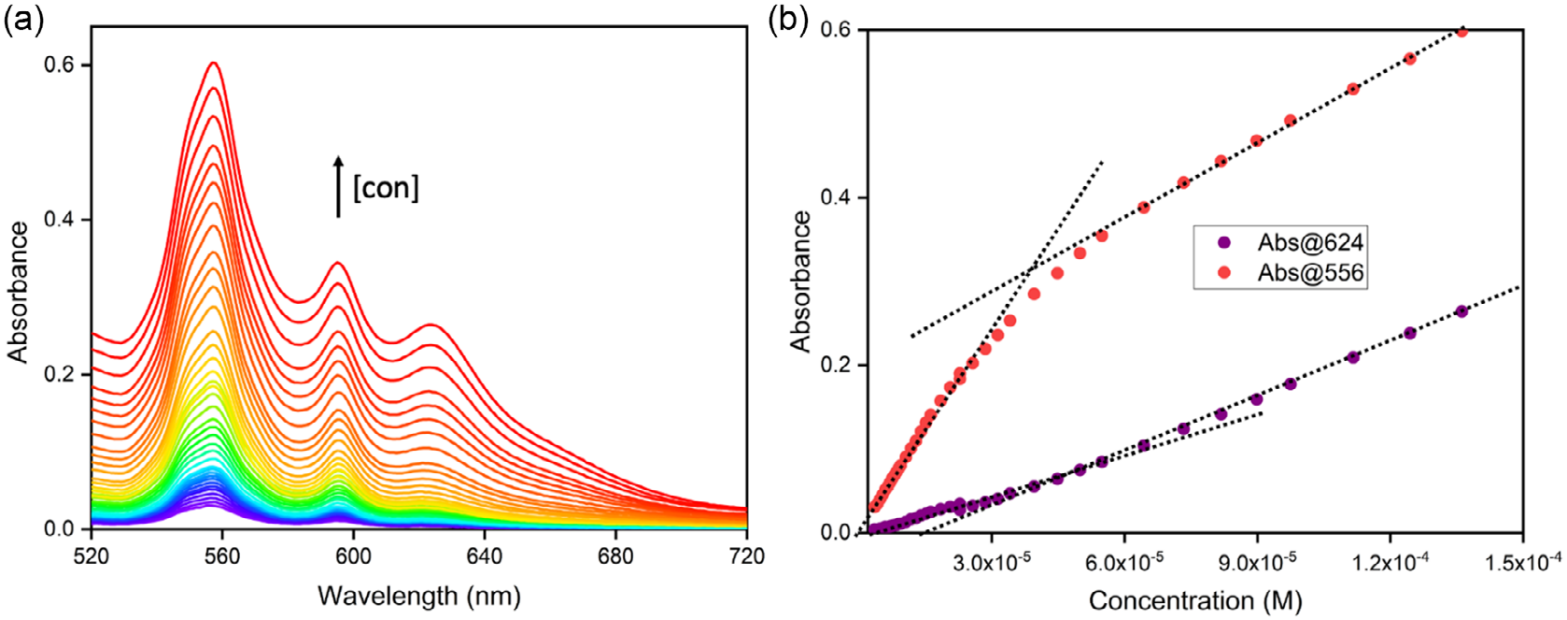
a) Absorption spectrum of AlC7P with increasing concentration from 1.9 × 10^−6^ to 1.5 × 10^−4^ M in hexanes. b) Beer–Lambert plot of AlC7P at 556 and 624 nm in hexanes at room temperature, 25 °C.

To further establish the aggregation, a Beer–Lambert plot was constructed, showing absorbance versus concentration at room temperature in hexanes. The absorbance spectra were measured across a range of concentrations from 1.9 × 10^−6^ M to 1.5 × 10 ^−4^ M. Concentration versus absorbance was plotted at two different wavelengths: 556 and 624 nm. The nonlinear relationship observed in **Figure** **6** indicates that aggregation occurs at room temperature. At a concentration of ≈ 5 × 10^−5^ M, the slope of the absorbance at 556 nm (the monomer peak) becomes shallower, while at the same concentration, the slope at 624 nm (the aggregate peak) becomes slightly steeper. This finding clarifies the results of the temperature‐dependent absorbance spectra. For the Soret band, the concentration of AlC7P in solution was 4.01 × 10^−6^ M, which is below the aggregation concentration at room temperature. In contrast, the concentration of AlC7P used to measure the Q‐bands was 1.42 × 10^−4^ M, exceeding the aggregation concentration at room temperature. This explains why the thermochromic properties were much less pronounced in the Soret band measurements compared to the Q‐band measurements.

T‐dependent fluorescence studies were performed on AlC7P using an excitation wavelength of 576 nm, corresponding to an isosbestic point in the Q‐band region of the absorption spectra. As shown in Figure 5c, at a low temperature (5 °C), the fluorescence spectrum exhibited peaks at 603, 640, 657, and 705 nm. According to quantitative fluorescence studies (see Figure 3b), the bands at 603 and 657 nm are attributed to the monomer, while the bands at 640 and 705 nm are associated with the aggregate form. As the temperature increased, the 640 and 705 nm peaks began to disappear, while the peaks at 603 and 657 nm grew significantly stronger. A sigmoidal curve of the fluorescence intensity at 657 nm as a function of temperature (Figure 5d), along with the reproducibility of fluorescence intensity at 5 and 55 °C over six repeated cycles (Figure 5f), establishes the robustness of the system. These findings further support the absorption studies, indicating that AlC7P exists primarily in aggregate form at lower temperatures, which breaks down as the temperature rises.

The optical studies can be used to reveal the nature of aggregation. In general, porphyrin aggregation can occur in two types: J‐ and H‐aggregation.^[^ ^53^, ^54^, ^55^, ^56^ ^]^ During these processes, the Soret band absorption feature changes distinctly as blue and red shifts will result in the H‐ and J‐aggregation, respectively. The fact that the 400 nm band increases with increasing concentration or decreases with increasing temperature suggests the H‐aggregation ‘face‐to‐face’ alignment is involved in these studies (Figure S6, Supporting Information).

**Figure 7:**
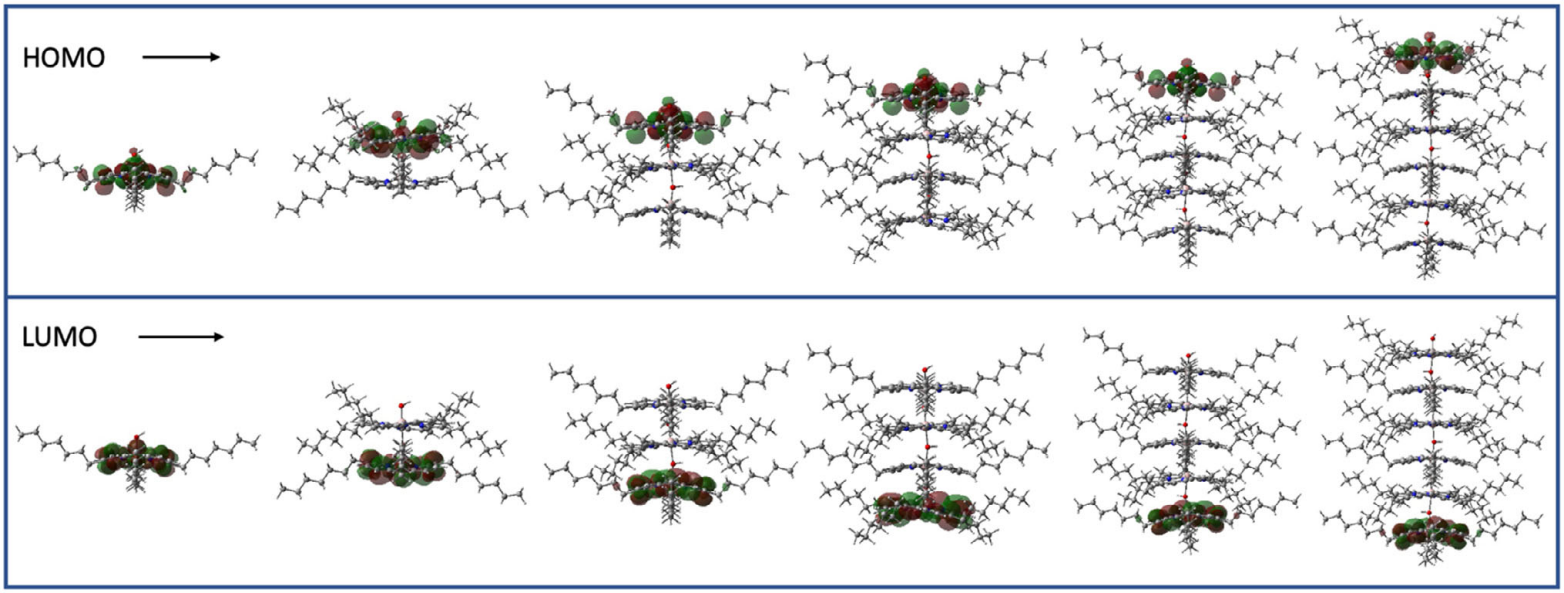
DFT calculated frontier orbitals (HOMO and LUMO) of series of H‐aggregated stacks.

The optical studies indicate that the molecular and electronic structures are very different at low and high temperature/concentration conditions. The aggregates have different absorbances than the monomers, giving the solution its thermochromic properties. To further elucidate the geometry and electronic structures of AlC7P, density functional theory (DFT) calculations were performed. **Figure** **7** shows optimized structures on a Born–Oppenheimer potential energy surface of a series of H‐aggregated stacks calculated using the B3LYP functional and the 6‐311 G(d, p) basis set as parameterized in Gaussian 16.^[^ ^57^ ^]^ The additional DFT details and the optimized atom coordinates (Table S2, Supporting Information) are given in the supporting information. The two porphyrin rings are arranged face‐to‐face, with four heptyl groups on each porphyrin extending outward in a staggered fashion to reduce steric repulsion. The aluminum center is situated in the plane of the porphyrin. When analyzing the monomer of AlC7P, it is observed that the highest occupied molecular orbital (HOMO) and lowest unoccupied molecular orbital (LUMO) are localized on the porphyrin ring, with an estimated HOMO–LUMO gap of 2.74 eV. In contrast, the stacked dimer exhibits a HOMO–LUMO gap of 2.02 eV, which is 0.72 eV lower than that of the monomer. In this dimer, the porphyrin containing the free LB (—OH group) functions as the HOMO, while the porphyrin with the free LA (aluminum center) serves as the LUMO. This behavior is consistent across all calculated stack sizes, extending up to six AlC7P units in the largest stack examined in this study.

**Figure 8:**
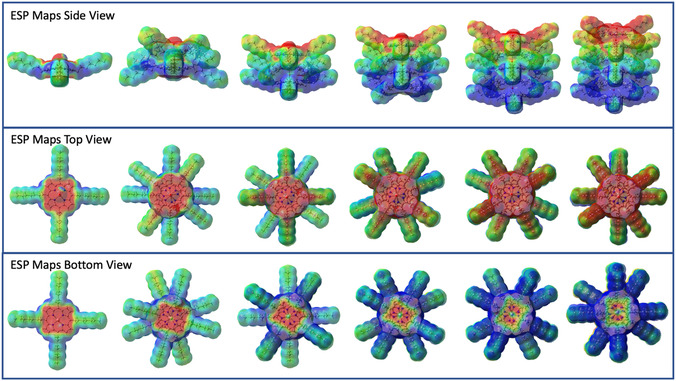
DFT calculated ESP map of series of H‐aggregated stacks. Red and blue colors indicate electron rich and poor regions, respectively.

Electrostatic potential (ESP) maps were computed for these H‐type aggregated stacks, see **Figure** **8**. Interestingly, a dipole is generated within the stacks due to electron disparity: the side with the free LB (the hydroxy unit) becomes more electron‐rich, while the side with the free LA (the Al center) becomes more electron‐poor. This uneven charge distribution increases as the number of AlC7P units in the aggregates rises. One significant consequence of this dipole is its impact on charge transfer properties. The spectral features observed at high concentrations or low temperatures in absorption and fluorescence studies likely arise from charge transfer transitions, which involve the movement of electron density from the electron‐rich portion to the electron‐poor portion of the stack. The long‐wavelength absorption feature at elevated or reduced temperatures may result from these charge transfer transitions. Similarly, the long‐wavelength fluorescence features and their reduced intensities can also be attributed to the charge transfer properties in the aggregates.

Time‐resolved fluorescence studies of AlC7Por were conducted in hexanes, varying temperature and concentration. The samples were excited at 560 nm, and emission was recorded at the high‐energy fluorescence band of 655 nm. Figure S7, Supporting Information, illustrates the fluorescence decay profiles, and the data on excited singlet‐state lifetimes are summarized in Table S1, Supporting Information. For discussion purposes, we considered the average values. The fluorescence lifetimes of AlC7P remained consistent at ≈5.50 ns across different temperatures and concentrations. This finding suggests that the aggregated compound is dark and nonfluorescent; thus, the lifetimes observed are attributed solely to the free‐existing monomer, which is in equilibrium with the aggregated species. According to Kasha's model, the emissive property of H‐aggregates is influenced by spin‐allowed and forbidden transitions.^[^ ^58^, ^59^, ^60^, ^61^ ^]^ In H‐type dimers with a ‘face‐to‐face’ alignment, the radiative decay is suppressed because the transition is symmetry‐forbidden, rendering the aggregates nonemissive.

## 3 Physical Methods

### 3.1 NMR and Mass Spectroscopy

NMR spectra were recorded on a Bruker Advance 400 MHz NMR spectrometer using CDCl_3_ or DMSO‐*d*_6_ as the solvent. ESI mass spectra were recorded on a Bruker MicroTOF‐III mass spectrometer using direct injection from an UltiMate 3000 High‐performance liquid column and CH_3_CN as a solvent.

### 3.2 Absorption and Emission Spectroscopy

UV/Vis spectra were recorded using an Agilent Cary 100 UV/Vis spectrometer. Variable temperature absorption studies were conducted with a Varian Cary 50 UV–Vis Spectrophotometer, which was equipped with an Agilent Cary Single Cell Peltier Accessory. To prevent solvent evaporation and maintain a constant concentration during measurements, the sample cuvette was sealed, and the temperature was varied from 5 to 55 °C. Steady‐state fluorescence spectra were captured using a Photon Technologies International Quanta Master 8075‐11 spectrofluorometer, which features a 75 W Xenon lamp and operates with FelixGX software. An excitation wavelength of 576 nm was chosen as the isosbestic point in the Q‐band region of the absorption spectra. Additionally, a Varian Cary Eclipse Fluorescence Spectrophotometer with a Varian Cary Single Cell Peltier Accessory was used for the temperature‐dependent spectra. The excitation and emission slit widths were set to 2.5 and 5.0 nm, respectively. Again, to prevent solvent evaporation and maintain a consistent concentration, the temperature was varied from 5 to 55 °C, and the sample cuvette was sealed. To enhance the clarity of the results and reduce the signal‐to‐noise ratio, the emission spectrum at each temperature was calculated as the average of five scans.

### 3.3 DFT Calculations

All of the amphiphilic aluminum(III) porphyrin structures were initially constructed on a local PC using the *GaussView 6* (GV6.0) software. DFT computations were performed on a supercomputer using the *Gaussian 16* software suite.^[^ ^57^ ^]^ The B3LYP was the DFT method chosen for this study. The 6‐31 G(d,p) split‐valence polarized basis set was used to model the compounds. Thus, the B3LYP method was coupled with the 6‐31 G(d,p) basis to form the B3LYP/6‐31 G(d,p) model chemistry was used to optimize the geometry of all the structures herein to a stationary point on the Born–Oppenheimer surface and calculate the first five excited singlet states of all chemical species in the current study. All the structures were optimized *sans* symmetry constraints as neutral closed‐shell singlets. The self‐consistent field convergence constraints and the DFT grid utilized in the calculation were the G16 default values, “Tight” and “UltraFine,” respectively. The optimization of the geometrical parameters of each of the chemical species in the study was continued until the maximum force, root‐mean square (RMS) force, maximum displacement, and RMS displacement reached or was less than, the default *Gaussian 16* minima and the predicted energy change upon a successive optimization cycle of the geometrical parameters was in the range of −5×10^−9^ A.U.

### 3.4 Time‐resolved Fluorescence Spectroscopy

A time‐correlated single‐photon‐counting apparatus utilizing a picosecond‐pulsed diode laser was used to measure the porphyrin fluorescence decay. Excitation pulses were delivered at 560 nm by a picosecond diode laser (PicoQuant, PDL 800‐B), 54 ps FWHM, at a repetition rate of 10 MHz. The porphyrin fluorescence was measured by a Hamamatsu R3809 microchannel plate photomultiplier screened by a double monochromator. A single‐photon‐counting PC card (Becker & Hickl, SPC‐730) was used for data collection. The instrument response time of the system was 80 ps.

## 4 Conclusion

The thermochromic properties have been effectively induced in aluminum(III) porphyrin by utilizing Lewis acid–base interactions and hydrophobic characteristics. The introduction of hydrophobic C7 chains enhances the solubility of aluminum(III) porphyrin and enables its thermochromic behavior. When placed in a nonpolar solvent, AlC7P demonstrates thermochromic properties due to the aggregation of the porphyrin at lower temperatures, which separates into monomers when heated. This transition primarily occurs within the temperature range of 5 to 55 °C. A noticeable color shift occurs as the temperature changes, accompanied by fluorescence quenching at lower temperatures. These characteristics suggest that AlC7P has promising potential as a LMT.

## Experimental Section

1

1.1

##### General

The chemicals and solvents were procured from Alfa Aesar, Acros Organics, Fisher Chemical, Sigma‐Aldrich, and Tokyo Chemical Industry (TCI). Chromatographic materials were acquired from Sigma‐Aldrich or SiliCycle. The 5,10,15,20‐tetraheptylporphyrin (H_2_C7P) and its Al(III) derivative 5,10,15,20‐tetraheptylaluminum(III) porphyrin (AlC7P) were prepared as described below.

##### Synthesis of 5,10,15,20‐Tetraheptylporphyrin (H_2_C7P)

A reaction mixture was prepared by dissolving pyrrole (1.88 g, 1.94 mL, 28.0 mmol) and octanal (3.60 g, 4.38 mL, 28.0 mmol) in dry CH_2_Cl_2_ (750 mL). The flask was flushed with N_2_ for 1 h, and BF_3_.Et_2_O (0.07 g, 0.08 mL, 0.49 mmol) was added, turning the solution red. The reaction was stirred at room temperature in the dark for 2 h. *p*‐Chloranil (6.88 g, 28.0 mmol) was added to the solution under a stream of N_2_. The solution was stirred at room temperature for an additional 12 h, turning the solution dark in color. The solution was vacuum filtered to remove solid impurities and concentrated under reduced pressure. The product was purified on a short Al_2_O_3_ column. The column was eluted with CH_2_Cl_2_: hexanes (50:50). This purification was repeated on another Al_2_O_3_ column. The collected pure compound was a purple solid. Yield: 425 mg (9%). ESI MS: *m/z* 703.5400 for [M + H]^+^, calculated 703.5674 for C_48_H_71_N_4_^+^. ^1^H NMR (400 MHz, CDCl_3_): *δ, ppm* 9.47 (s, 8 H), 4.94 (t, 8 H, *J* = 7.92 Hz), 2.52 (q, 8 H, *J* = 7.68 Hz), 1.81 (q, 8 H, *J* = 7.48 Hz), 1.54 (q, 8 H, *J* = 6.72 Hz), 1.37 (m, 8 H), 1.26 (bm, 8 H), 0.91 (m, 12 H), and −2.63 (s, 2 H).

##### Synthesis of 5,10,15,20‐tetraheptylaluminum(III) porphyrin (AlC7P)

A solution of H_2_C7P (55 mg, 0.074 mmol) in 5 mL of dry toluene was prepared, and trimethylaluminum (0.2 mL, 2.0 M in hexane) was added under an atmosphere of N_2_. The solution was stirred under a dark cloth at room temperature for 19 h. About 1 mL of water was then added dropwise, and the solution was stirred at room temperature for an additional 22 h. The toluene was removed under reduced pressure, and the compound was purified using an Al_2_O_3_ column with CH_2_Cl_2_: CH_3_OH (= 98:2) eluent. The pure compound was collected as a dark green solid. Yield: 53 mg (91%). ESI MS: *m/z* 745.5325 for [M + H]^+^, calculated 745.5365 for C_48_H_70_AlN_4_O^+^. ^1^H NMR (400 MHz, CDCl_3_ @ 50 °C): *δ, ppm* 9.50 (s, 8 H), 4.72 (m, 8 H), 2.39 (m, 8 H), 1.62 (m, 8 H), 1.39 (m, 8 H), 1.25 (m, 8 H), and 0.83 (m, 20 H). The ^1^H NMR spectrum of this compound contained two sets of peaks, one for the aggregated compound and one for the nonaggregated compound. This made interpretation of the NMR difficult, as some peaks were not distinct.

## Conflict of Interest

The authors declare no conflict of interest.

## Supporting information

Supplementary Material

## Data Availability

The data that support the findings of this study are available in the supplementary material of this article.
