# Supplementary material for: Temperature Tunable Optical Properties of Tetraheptylaluminum(III) Porphyrin Toward Molecular Thermometer
Source: Chemphyschem. 2025 May 14;26(13):e202500087. doi: 10.1002/cphc.202500087 (PMC12225750; doi:10.1002/cphc.202500087)
Supplement: Supplementary file 1 — Supplementary Material [file CPHC-26-e202500087-s001.pdf]

Supporting Information

Temperature Tunable Optical Properties of Tetraheptylaluminum(III) Porphyrin Towards Molecular Thermometer

Stefan Charon,[a] *Niloofar Zarrabi,*[b] *Jatan K. Sharma,*[c] *Paul A. Karr,*[d] *Francis D'Souza,*[c] *Prashanth K. Poddutoori**[,a,b]

[a]Advanced Materials Center, University of Minnesota Duluth, 1038 University Drive, Duluth, Minnesota 55812, USA.

[b]*Department of Chemistry & Biochemistry*, University of Minnesota Duluth, 1038 University Drive, Duluth, Minnesota 55812, USA.

[c]Department of Chemistry, University of North Texas, 1155 Union Circle, # 305070, Denton, Texas 76203-5017, USA.

[d]Department of Physical Sciences and Mathematics, Wayne State College, 1111 Main Street, Wayne, Nebraska 68787, USA.

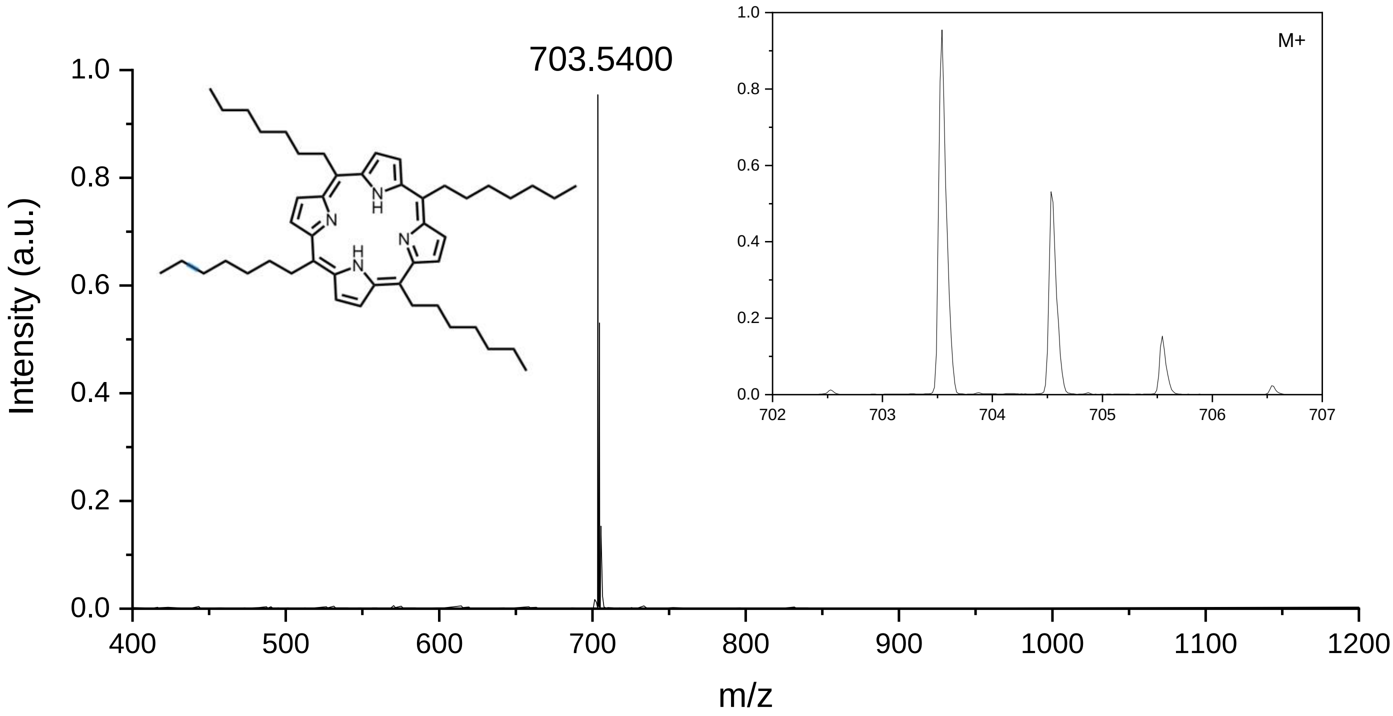

Figure S1. ESI-MS spectrum of 5,10,15,20-tetraheltylporphyrin H_2C7P.

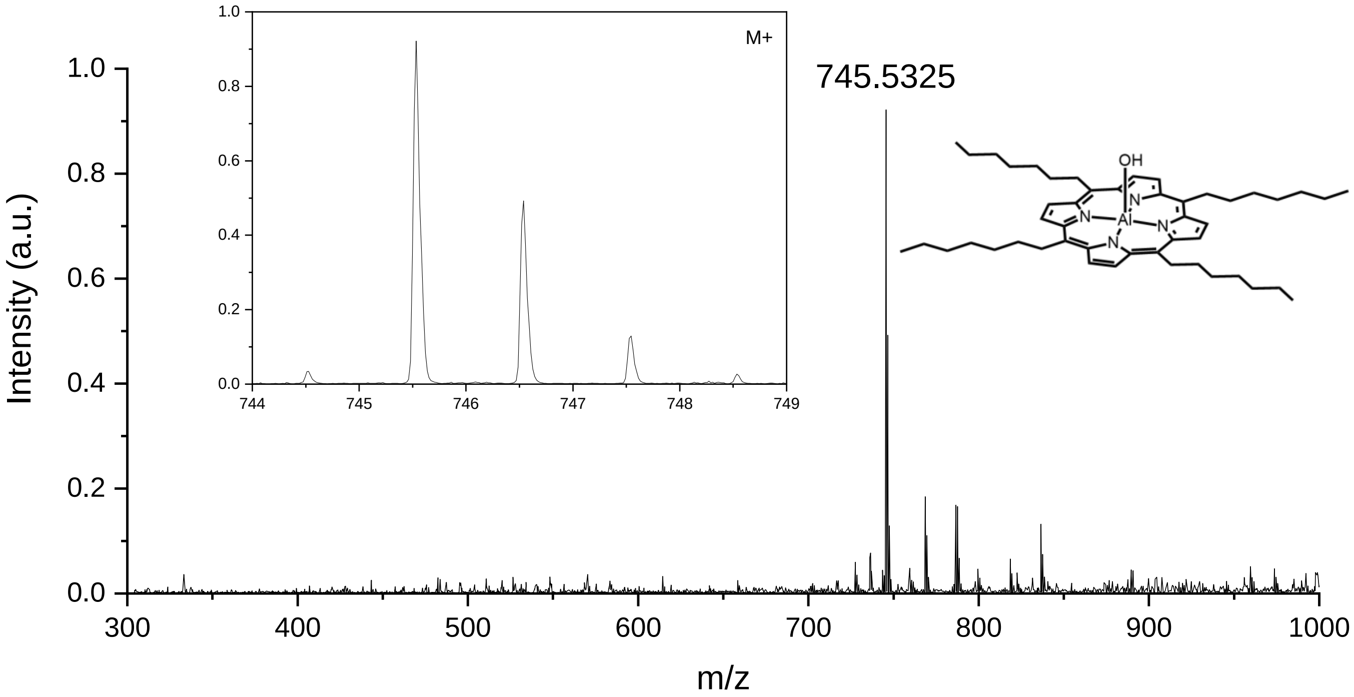

Figure S2. ESI-MS spectrum of 5,10,15,20-tetraheptylaluminum(III) porphyrin (AlC7P).

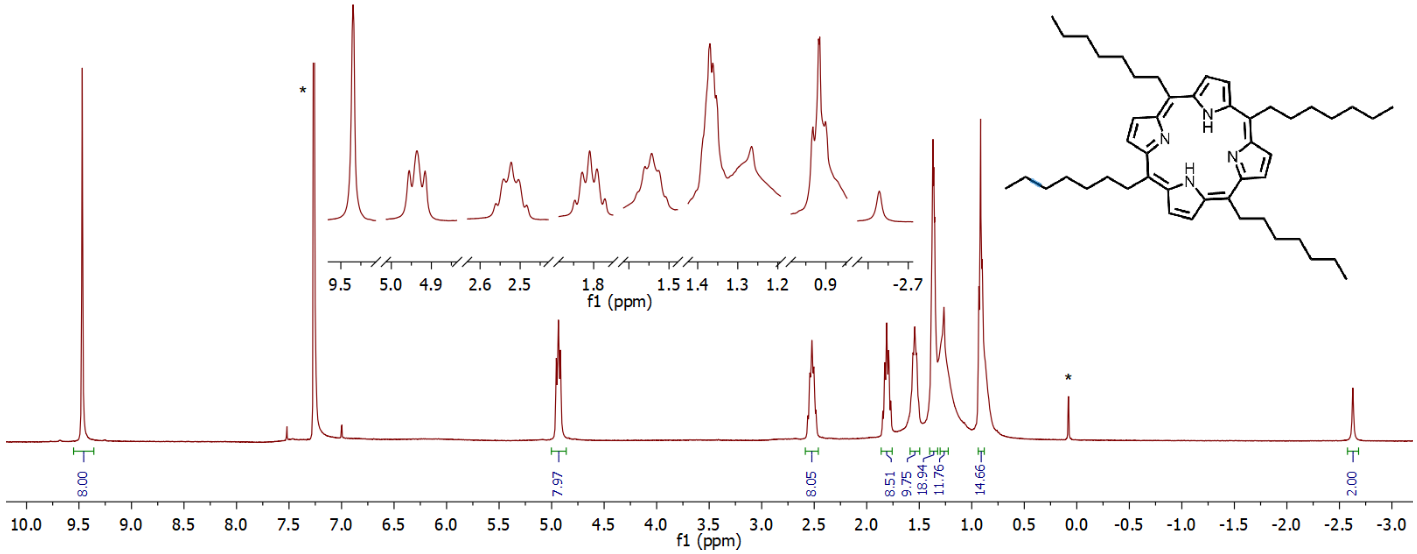

Figure S3. ^{1}HNMR spectrum of 5,10,15,20-tetraheptylporphyrin (H_2C7P) in $CDCl_3$.

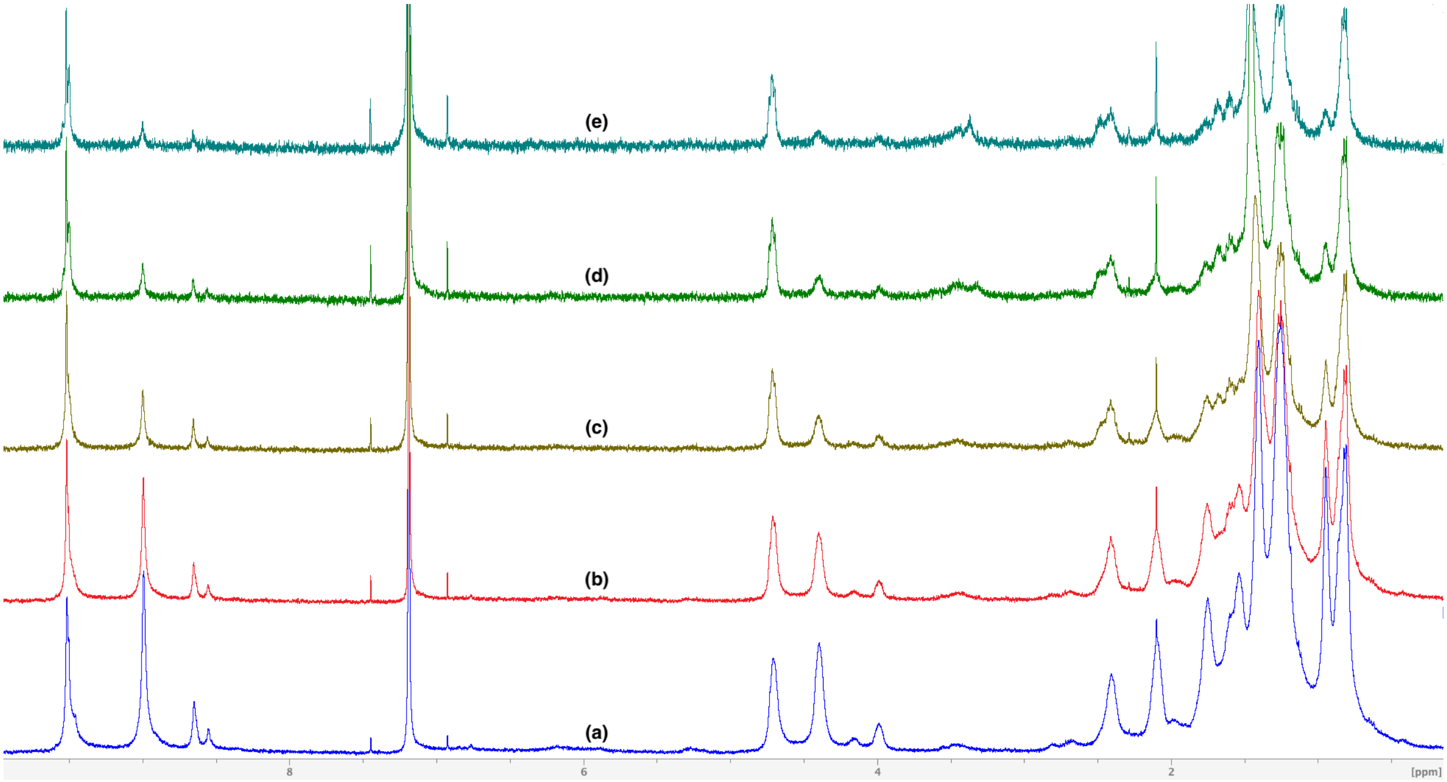

Figure S4. ^{1}H NMR spectra of 5,10,15,20-tetraheptylaluminum(III) porphyrin (AlC7P) in $CDCl_3$ at concentrations of (a) 4.3×10^{-3} M, (b) 2.1×10^{-3} M, (c) 1.1×10^{-3} M, (d) 5.4×10^{-4} M, (e) 2.7×10^{-4} M.

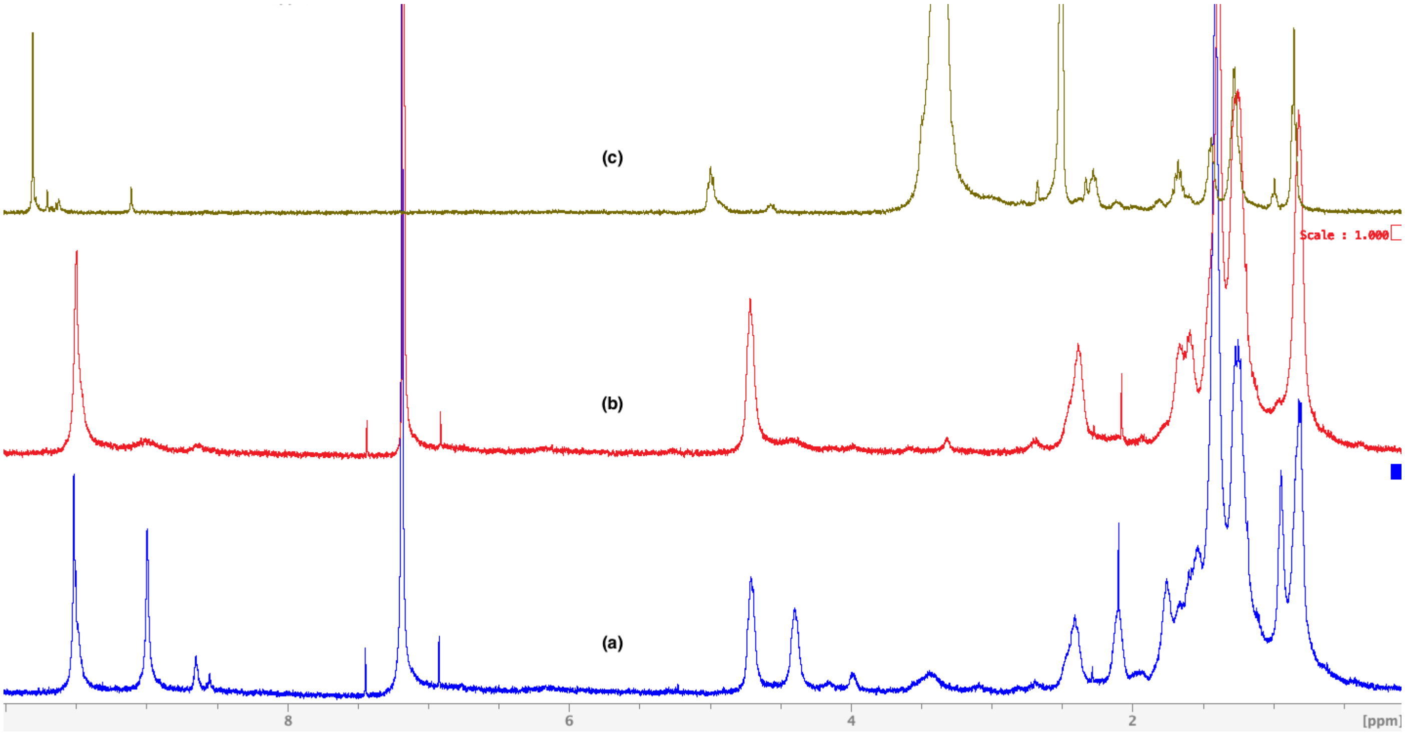

Figure S5. ^{1}H NMR spectra of 5,10,15,20-tetraheptylaluminum(III) porphyrin (AlC7P) in $CDCl_3$ (a) at 25°C, (b) at 50°C, and (c) in DMSO-d_6.

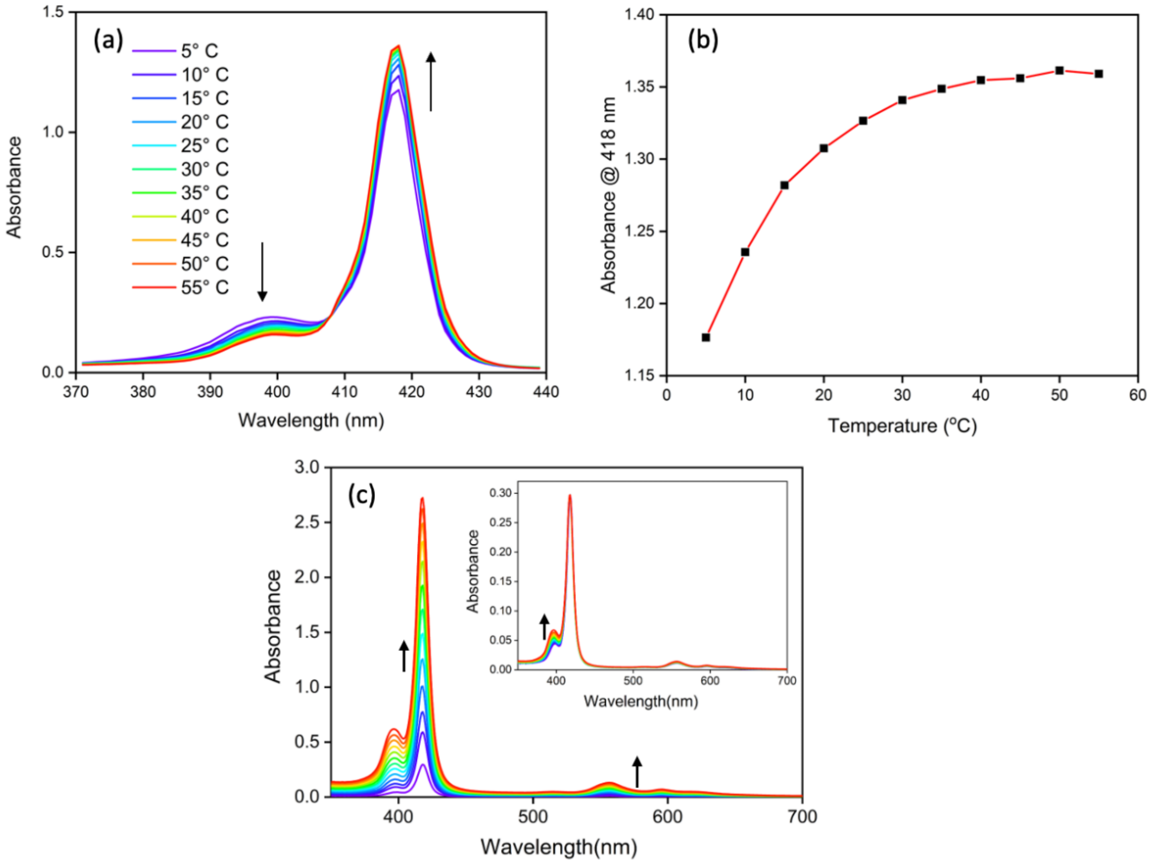

Figure S6. Temperature dependent (a) absorbance @B-band concentration 4.01×10^{-6} M, (b) Absorbance *vs* T curve at 418 nm, and (c) concentration (1.58×10^{-6} - 1.74×10^{-5} M) dependent absorption spectra of AlC7P in hexanes.

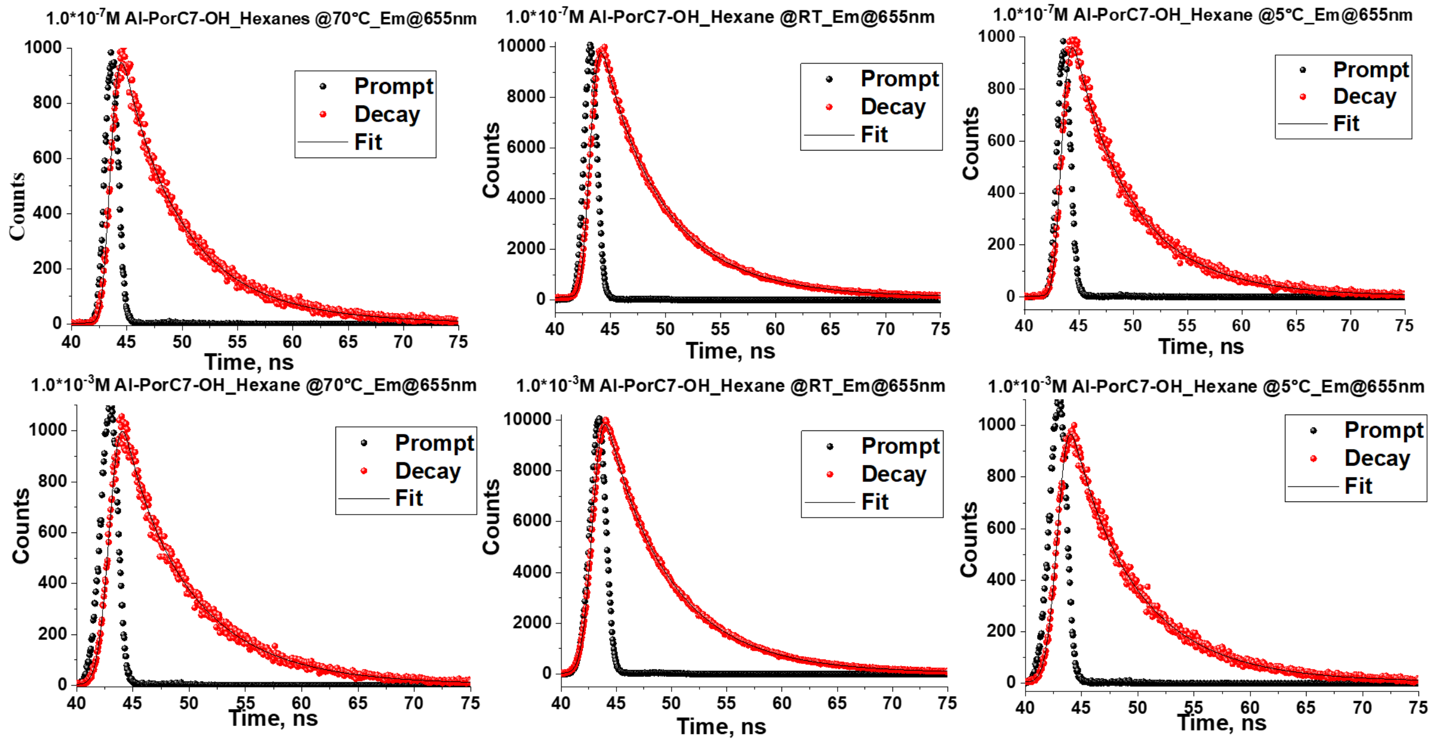

Figure S7. Decay profiles of AlC7P at various concentrations and temperatures in hexanes.

Table S1. Lifetime data of AlC7P in hexane. Excitation at 555 nm, Emission collected at 655 nm.

**Temperature**	**Chi.sq.**	**Avg. Lifetime (ns)**
Concentration = 1.0 $\times 10^{-7}$ M		
70°C	1.10	5.51
RT	1.18	5.56
5°C	1.19	5.42
Concentration = 1.0 $\times 10^{-3}$ M		
70°C	1.19	5.64
RT	1.08	5.65
5°C	1.16	5.63

DFT calculation details

Assembly of Tetraheptylaluminum(III) Porphyrin. The tetraheptylaluminum(III) Porphyrin and Tetraheptylaluminum(III) Porphyrin aggregate structures studied herein were assembled on a local pc using the *GaussView* 6.0[ref] program. During the assembly process, the bond lengths, bond angles, and dihedrals were carefully chosen based on valence bond and hybridization theory. The *GaussView* "clean" function was avoided as it is known to distort the porphyrin structure. Once the structures were assembled, they were uploaded to a supercomputer where final editing for optimization was completed.

Ground state optimization of Tetraheptylaluminum(III) Porphyrin and Aggregates. The self-consistent field (SCF) convergence constraints and the DFT grid used in the calculations were the *Gaussian 16* default values, Tight and UltraFine, respectively. The optimization of each chemical species continued until the maximum force, root mean square (rms) force, maximum displacement, and rms displacement reached the default *Gaussian 16* minima, and the predicted energy change upon another geometry optimization cycle was in the range of $-5x10^{-9}$ A.U.

To avoid SCF (Self-Consistent Field) convergence failure, initial DFT computations of the tetraheptylaluminum(III) Porphyrin and aggregate structures were performed on a supercomputer via the *Gaussian 16* software suite *sans* symmetry constraints *in-vacuo* as neutral species, utilizing the modest B3LYP/3-21G* split-valence model chemistry. After convergence to a stationary point on the Born-Oppenheimer surface with the B3LYP/3-21G* model chemistry, the resulting geometry was then optimized using the B3LYP/6-31G model chemistry under the same constraints. Finally, using the previously optimized structures of tetraheptylaluminum(III) porphyrin and aggregates as input, the 6-31G(d,p) basis set was coupled with the B3LYP method to form the B3LYP/6-31G(d,p) model chemistry which was utilized to optimize the molecular structures *sans* symmetry constraints *in-vacuo* to a stationary point on the Born-Oppenheimer surface.

Upon final optimization, FMO (HOMO-2, HOMO-1, HOMO, LUMO, LUMO+1, and LUMO+2) cube files were generated on the supercomputer using the G16 cubegen function. (cubegen 0 MO=*nnn filename*.fch *filename*-HOMO.cub). Where "*nnn*" is the MO number of the HOMO and "*filename*" is the name of the formatted check file. At the same time cube files of the electron density and potential of the ground state were generated also using the G16 cubegen function. (cubegen 0 density=scf *filename*.fch *filename* -Den.cub 0 h) and (cubegen 0 potential=scf *filename*.fch *filename* -POT.cub 0 h). Following *in-vacuo* optimization, to investigate the impact of solvation, tetraheptylaluminum(III) porphyrin and aggregates, were optimized (using the same model chemistry delineated above) in *n*-hexane and dimethylsulfoxide utilizing the self-consistent reaction field (SCRF) with the conductor polarizable continuum model (CPCM). Investigation of the impact on aggregation energy of the heptyl structures was performed by removing all four heptyl structures, replacing the structures with hydrogen atoms, and optimizing the structures using the same model chemistry delineated above.

Table S2. Optimized atom coordinates for their DFT structures.

(a) Mono Stack

C	1.83216800	3.83068400	-0.14371300
C	2.91751500	3.07383800	0.17015500
C	0.70984800	2.93715200	-0.24495100
C	2.48358700	1.70250800	0.20630100
N	1.12632000	1.63160600	-0.05336800
C	2.91404200	-0.71009100	0.27285900
C	3.80211700	-1.84325700	0.28686000
C	3.05900100	-2.93749900	-0.02530200
N	1.62305400	-1.12632200	0.00467000
C	1.69803200	-2.49537100	-0.17475300
C	-0.70911000	-2.93302800	-0.22893100
C	-1.83264100	-3.82669700	-0.13156900
C	-2.91672400	-3.07034700	0.18372000
C	-2.47998400	-1.69912700	0.22361300
N	-1.12377500	-1.63014300	-0.02871700
C	-1.69694800	2.49692400	-0.17625800
C	-3.05883300	2.93859600	-0.02986200
C	-3.80039100	1.84528400	0.28686600
C	-2.91063200	0.71300500	0.27865800
N	-1.62035800	1.12920700	0.01129000
C	3.34047700	0.61545800	0.41543000
C	-0.61680600	3.36253400	-0.38413100
C	-3.33701400	-0.61165600	0.42794600
C	0.61551100	-3.36015100	-0.37510200
H	3.92409800	3.42265900	0.34499500
H	1.79673200	4.90413100	-0.25368600
H	-3.40592900	3.95666300	-0.12232400
H	-4.86135200	1.81391400	0.48331600
H	-3.92445500	-3.41767200	0.35432700
H	-1.79840100	-4.89938000	-0.24831200
H	3.40467700	-3.95663900	-0.11143500
H	4.86299000	-1.81264000	0.48426400
Al	-0.00762400	-0.00621600	-0.49724700
C	4.80631400	0.88591000	0.71485800
C	5.69536800	1.01313000	-0.53992600
H	5.20070700	0.09054000	1.35428900
H	4.89462500	1.79930000	1.31072500
C	7.16708900	1.28428400	-0.20606500
H	5.61311700	0.09331600	-1.13277100
H	5.30548500	1.81794600	-1.17610400
H	7.24209600	2.20410300	0.39186000
H	7.55023900	0.47722800	0.43505200
C	-0.89239400	4.83754700	-0.62860400
C	-1.01677700	5.68779400	0.65371300
H	-1.80890500	4.94096200	-1.21760100
H	-0.09954200	5.25341400	-1.25814200
C	-1.29352200	7.16797700	0.36465900

H	-1.81714700	5.27743300	1.28221400
H	-0.09492100	5.59137800	1.24105900
H	-0.49083000	7.57217900	-0.26894800
H	-2.21642500	7.25787900	-0.22630100
C	-4.80372300	-0.88097400	0.72329300
C	-5.68772600	-1.00950800	-0.53501100
H	-5.20007000	-0.08474800	1.36056800
H	-4.89416900	-1.79361400	1.31997900
C	-7.16038900	-1.28142900	-0.20602500
H	-5.60332400	-0.09037000	-1.12832700
H	-5.29429000	-1.81366200	-1.16945200
H	-7.23721100	-2.20129700	0.39170500
H	-7.54664500	-0.47458800	0.43364600
C	0.88893600	-4.83485200	-0.62238700
C	1.01152600	-5.68801800	0.65815100
H	1.80550600	-4.93816700	-1.21127400
H	0.09581500	-5.24730700	-1.25356800
C	1.28618100	-7.16797000	0.36592100
H	1.81218400	-5.28042500	1.28823900
H	0.08951300	-5.59160400	1.24523100
H	0.48329400	-7.56929700	-0.26918400
H	2.20933300	-7.25798200	-0.22465500
C	8.05979100	1.41246800	-1.44661600
C	9.53275300	1.68342200	-1.11712900
H	7.67650000	2.21962000	-2.08689100
H	7.98447600	0.49257100	-2.04362300
C	10.42578800	1.81138200	-2.35734800
H	9.60867700	2.60371700	-0.52019600
H	9.91665200	0.87627100	-0.47669800
C	11.89505200	2.08181100	-2.02002900
H	10.35016900	0.89173500	-2.95335200
H	10.04242000	2.61806100	-2.99688700
H	12.50503500	2.16770200	-2.92512400
H	12.00691500	3.01384700	-1.45434900
H	12.31693400	1.27456700	-1.41047100
C	1.40832400	-8.02652600	1.63095800
C	1.68299000	-9.50751000	1.34313800
H	0.48485700	-7.93643400	2.22044700
H	2.21125400	-7.62496300	2.26537800
C	1.80430200	-10.36653500	2.60781400
H	0.88027800	-9.90959800	0.70835800
H	2.60701000	-9.59832400	0.75413600
C	2.07856600	-11.84401700	2.31202000
H	2.60654200	-9.96494100	3.24176200
H	0.88085300	-10.27610100	3.19586200
H	2.15953900	-12.42927500	3.23375800
H	1.27566100	-12.28324900	1.70903900
H	3.01422100	-11.96982100	1.75533100
C	-8.04833600	-1.41032000	-1.44992100
C	-9.52231100	-1.68248700	-1.12603300
H	-7.66179000	-2.21696100	-2.08875100
H	-7.97136100	-0.49039900	-2.04660800
C	-10.41052200	-1.81093500	-2.36966100
H	-9.59984700	-2.60298900	-0.52957800
H	-9.90940200	-0.87578900	-0.48688300
C	-11.88086600	-2.08266300	-2.03810500
H	-10.33334400	-0.89112500	-2.96520000
H	-10.02399600	-2.61710700	-3.00789900
H	-12.48734200	-2.16886600	-2.94555100
H	-11.99414100	-3.01493000	-1.47305700
H	-12.30579400	-1.27593100	-1.42994400
C	-1.41790400	8.02339100	1.63161500
C	-1.69469900	9.50460500	1.34705800
H	-0.49474100	7.93334300	2.22159600
H	-2.22067100	7.61904700	2.26442600
C	-1.81839100	10.36042100	2.61368400
H	-0.89212800	9.90951300	0.71386800
H	-2.61840200	9.59532200	0.75756300
C	-2.09477200	11.83815600	2.32116900
H	-2.62051100	9.95603800	3.24598700
H	-0.89528100	10.27004100	3.20227900
H	-2.17743300	12.42106100	3.24423800
H	-1.29207900	12.28012900	1.71990200
H	-3.03018000	11.96378700	1.76403900
O	-0.07589900	-0.06705200	-2.24265700
H	0.59007400	0.41968400	-2.73608400

(b) Di Stack

C	1.07164500	4.03611200	2.59749700
C	-0.23262100	4.18661300	2.24670000
C	1.35961100	2.62680300	2.56593800
C	-0.77288400	2.86824800	2.05310800
N	0.21535900	1.91859700	2.24134600
C	-2.66030200	1.32028100	1.92431000
C	-4.06537400	1.04481300	2.04456400
C	-4.19082800	-0.27763200	2.33311900
N	-1.93538700	0.15007800	2.07576200
C	-2.86878000	-0.84065900	2.33310800
C	-1.31574400	-2.72954000	2.35648400
C	-1.02349900	-4.13725200	2.35304200
C	0.29492200	-4.27196700	2.04987100
C	0.83916900	-2.94669400	1.93090300
N	-0.16301600	-2.00737000	2.10347100
C	2.91784200	0.73630100	2.54085900
C	4.24284800	0.17757500	2.55270700
C	4.13108800	-1.12956500	2.19531900
C	2.73095000	-1.40052600	2.01591800
N	1.99607400	-0.24388100	2.21371300
C	-2.12940700	2.61074100	1.82543000
C	2.63739900	2.09000100	2.75743600
C	2.20616300	-2.67986800	1.79973300
C	-2.59854300	-2.19908700	2.52533800
H	-0.78269900	5.11141500	2.16199100
H	1.77568100	4.81895700	2.83527600
H	5.15152000	0.71216500	2.78412200
H	4.93018800	-1.84848400	2.09826800
H	0.85251600	-5.19168400	1.95851700
H	-1.73443000	-4.92883500	2.53540100
H	-5.10649700	-0.82017100	2.51261800
H	-4.85863400	1.77151900	1.96254200
Al	0.02994900	-0.03688700	1.70726100
C	-3.07562400	3.78212400	1.62291000
C	-3.62798900	4.39359100	2.92792500
H	-3.91127600	3.46320800	0.99380500
H	-2.56759700	4.55701900	1.04192800
C	-4.58489100	5.56522100	2.67711500
H	-4.14219600	3.61357600	3.50370700
H	-2.79183600	4.72689800	3.55554900
H	-4.06621200	6.34167600	2.09649900
H	-5.41881700	5.22550700	2.04589900
C	3.78111100	3.02646400	3.11182700
C	4.48873000	3.64384300	1.88708000
H	4.51643600	2.49110400	3.71962300
H	3.41084800	3.82943400	3.75627400
C	5.65873600	4.55801900	2.26888200
H	4.84237200	2.83888800	1.23079800
H	3.75499800	4.20771000	1.29719700
H	5.29783000	5.35570200	2.93423500
H	6.39228200	3.98639200	2.85574100
C	3.16242400	-3.83733000	1.56584900
C	3.66823900	-4.52393900	2.85226100
H	4.02087100	-3.48141700	0.98848600
H	2.67829300	-4.57741100	0.92235500
C	4.63352700	-5.68113400	2.56875100
H	4.16107900	-3.77938500	3.49020900
H	2.81006700	-4.89129200	3.42913100
H	4.13609600	-6.42146900	1.92569400
H	5.48989800	-5.30738800	1.98892200
C	-3.74547900	-3.13692800	2.86716500
C	-4.46023400	-3.75177100	1.64663900
H	-4.47855400	-2.60195400	3.47830400
H	-3.37531900	-3.94219300	3.50924300
C	-5.64313300	-4.64609200	2.03604000
H	-4.79951500	-2.94770000	0.98258800
H	-3.73487600	-4.33237900	1.06280800
H	-5.29478400	-5.44057800	2.71187700
H	-6.37052300	-4.05787800	2.61411000
C	-2.66271000	3.38472700	-2.18607100
C	-1.49459800	4.04823400	-1.97410000
C	-2.35262200	1.97365100	-2.19453900
C	-0.44794700	3.05283300	-1.90139500
N	-1.00083900	1.80267200	-2.02355800
C	1.94400600	2.40103700	-1.95655900
C	3.34599300	2.71893500	-2.09122000

C	4.00010500	1.55352200	-2.34832100
N	1.77177300	1.03982800	-2.08400100
C	3.01505200	0.49900900	-2.33375700
C	2.34550600	-1.88701100	-2.34511600
C	2.65792700	-3.29652700	-2.32373300
C	1.50130900	-3.95903200	-2.04887700
C	0.45773600	-2.96677500	-1.93729000
N	0.99831200	-1.71457000	-2.12061500
C	-3.01579900	-0.40953800	-2.11276700
C	-4.00480500	-1.46053900	-2.05494700
C	-3.33878600	-2.62516300	-1.83293400
C	-1.92882500	-2.30705000	-1.79448800
N	-1.76442700	-0.95048200	-1.93749600
C	0.92306200	3.35649200	-1.81283200
C	-3.31209100	0.95175100	-2.29889000
C	-0.90290600	-3.26723400	-1.74278300
C	3.30170500	-0.86613700	-2.50248200
H	-1.35903400	5.11769800	-1.90932000
H	-3.64418700	3.81948800	-2.30550400
H	-5.07254600	-1.33311400	-2.15613800
H	-3.77080900	-3.61014600	-1.74008800
H	1.37347300	-5.02757200	-1.95799200
H	3.63397400	-3.73282400	-2.47683900
H	5.05962300	1.43064900	-2.51789800
H	3.78192400	3.70514600	-2.03211800
Al	-0.03465800	0.04447000	-2.24009000
C	1.32559600	4.81639400	-1.66499800
C	1.48917200	5.56891400	-3.00193500
H	2.26229400	4.88139400	-1.10243100
H	0.58792100	5.33704900	-1.04663100
C	1.89631900	7.03567300	-2.81799800
H	2.23611100	5.05034800	-3.61592000
H	0.54830500	5.51212800	-3.56332700
H	1.14616000	7.54802300	-2.19778300
H	2.83801200	7.08510200	-2.25163800
C	-4.75328300	1.34072800	-2.59703500
C	-5.62721500	1.62454700	-1.35923500
H	-5.22283600	0.54817800	-3.18742100
H	-4.76122500	2.22063700	-3.24745800
C	-7.07964200	1.96234300	-1.71645200
H	-5.59926000	0.75630800	-0.68923100
H	-5.18612800	2.45564000	-0.79354900
H	-7.09642300	2.82286900	-2.40065400
H	-7.52061300	1.12531900	-2.27669500
C	-1.29648600	-4.72805800	-1.58232700
C	-1.54885000	-5.46595500	-2.91356300
H	-2.19388900	-4.79835800	-0.96050300
H	-0.52145600	-5.25620600	-1.01896400
C	-1.94997600	-6.93308000	-2.71940300
H	-2.33198900	-4.93851700	-3.47270900
H	-0.64510300	-5.40803700	-3.53330900
H	-1.16433000	-7.45463300	-2.15321800
H	-2.85410100	-6.98440300	-2.09501900
C	4.73989900	-1.26562200	-2.79890700
C	5.60676400	-1.51115900	-1.54702800
H	5.21221000	-0.49246700	-3.41230100
H	4.74853400	-2.16581200	-3.42101500
C	7.05946800	-1.86940400	-1.88194200
H	5.58016100	-0.61862200	-0.90918400
H	5.15582400	-2.31771100	-0.95423500
H	7.07631200	-2.75470700	-2.53388100
H	7.50891000	-1.05580400	-2.46933000
O	0.24291700	0.07261100	-0.07462000
C	2.06177800	7.79437000	-4.14063100
C	2.46945300	9.26160600	-3.96028500
H	1.12040800	7.74569400	-4.70591400
H	2.81160200	7.28243000	-4.76014500
C	2.63450500	10.02088400	-5.28253500
H	1.71954000	9.77418500	-3.34041600
H	3.41166100	9.31074600	-3.39531700
C	3.04202400	11.48510600	-5.09353100
H	3.38381200	9.50922600	-5.90170400
H	1.69318300	9.97267800	-5.84663000
H	3.15159500	11.99853000	-6.05436300
H	2.29435800	12.03231100	-4.50794000
H	3.99788600	11.56531300	-4.56319200
C	7.92453400	-2.13686700	-0.64404000
C	9.37999500	-2.48925500	-0.97433500

H	7.47790100	-2.95507100	-0.06028000
H	7.90467600	-1.25319800	0.00988500
C	10.24385700	-2.75943600	0.26369200
H	9.40044600	-3.37199500	-1.62944000
H	9.82821200	-1.67149300	-1.55670200
C	11.69577400	-3.10960900	-0.07550400
H	10.22344500	-1.87724300	0.91815000
H	9.79645800	-3.57728000	0.84509000
H	12.28518000	-3.29659100	0.82803700
H	11.75105400	-4.00836200	-0.70022900
H	12.18121200	-2.29603600	-0.62643800
C	-2.20252700	-7.67731500	-4.03646900
C	-2.60420500	-9.14487600	-3.84564700
H	-1.29871000	-7.62638400	-4.65997300
H	-2.98805400	-7.15620100	-4.60174400
C	-2.85620400	-9.88952700	-5.16251100
H	-1.81861100	-9.66663800	-3.28003200
H	-3.50879300	-9.19645200	-3.22246500
C	-3.25671900	-11.35428200	-4.96305200
H	-3.64138400	-9.36883100	-5.72730100
H	-1.95251000	-9.83853500	-5.78491500
H	-3.42931300	-11.85709800	-5.92020900
H	-2.47548100	-11.91013800	-4.43198800
H	-4.17718500	-11.43685800	-4.37378200
C	-7.95494100	2.27041800	-0.49528100
C	-9.40922000	2.60465400	-0.84890700
H	-7.51614100	3.11095600	0.06214600
H	-7.93702700	1.41080600	0.19016700
C	-10.28373400	2.91570200	0.37192700
H	-9.42783900	3.46313800	-1.53550100
H	-9.84974900	1.76466700	-1.40491500
C	-11.73418400	3.24775800	0.00905100
H	-10.26529600	2.05770400	1.05787700
H	-9.84391800	3.75565800	0.92695800
H	-12.33142400	3.46483600	0.90064800
H	-11.78772900	4.12321400	-0.64806100
H	-12.21228900	2.41300800	-0.51602700
C	-5.14372700	6.18347900	3.96461100
C	-6.10179700	7.35485900	3.71645900
H	-4.30983800	6.52364500	4.59497400
H	-5.66240500	5.40666000	4.54421400
C	-6.66186300	7.97355900	5.00314600
H	-5.58328900	8.13234300	3.13711900
H	-6.93590200	7.01505600	3.08576800
C	-7.61791800	9.14217000	4.74631100
H	-7.18013100	7.19672900	5.58166200
H	-5.82856800	8.31373100	5.63294400
H	-7.99987800	9.56109300	5.68292500
H	-7.11829700	9.95043600	4.20020800
H	-8.47987200	8.82508500	4.14827900
C	6.35891600	5.18700900	1.05784600
C	7.53385400	6.09847200	1.43227200
H	5.62556400	5.76177100	0.47433400
H	6.71605300	4.38912900	0.39145400
C	8.23237400	6.72819500	0.22080200
H	7.17720300	6.89638300	2.09944000
H	8.26828500	5.52403600	2.01506000
C	9.40549000	7.63572900	0.60256500
H	8.58806200	5.93086500	-0.44588100
H	7.49883700	7.30315700	-0.36061100
H	9.88177200	8.06897500	-0.28288700
H	9.07518100	8.46336000	1.24059100
H	10.17272200	7.08088400	1.15454400
C	5.14507200	-6.37445500	3.83753900
C	6.11031600	-7.53243800	3.55648000
H	4.28865500	-6.74801600	4.41670000
H	5.64318500	-5.63405000	4.47946400
C	6.62234600	-8.22695600	4.82434200
H	5.61241600	-8.27316000	2.91429300
H	6.96728900	-7.15919500	2.97745500
C	7.58563100	-9.38184800	4.53458400
H	7.12015900	-7.48694600	5.46566500
H	5.76621700	-8.60044900	5.40250700
H	7.93242700	-9.85611200	5.45839100
H	7.10529300	-10.15507900	3.92422300
H	8.46937800	-9.03335700	3.98804500
C	-6.34877500	-5.28021800	0.83084400
C	-7.53680500	-6.17135000	1.21291300

H	-5.62191100	-5.87214900	0.25623000
H	-6.69373900	-4.48580500	0.15393800
C	-8.24041300	-6.80648400	0.00723300
H	-7.19257300	-6.96566000	1.89081800
H	-8.26499500	-5.58002900	1.78653900
C	-9.42642900	-7.69373000	0.39675700
H	-8.58376300	-6.01272800	-0.67007600
H	-7.51302300	-7.39812100	-0.56512500
H	-9.90607100	-8.13142700	-0.48468100
H	-9.10879800	-8.51777900	1.04577200
H	-10.18770100	-7.12208100	0.93975100
O	-0.13396400	0.12892000	-4.01771300
H	0.18348700	-0.65396600	-4.47823100
H	1.14738500	0.39356600	-0.21475800

(c) Tri Stack

C	-2.96445500	-3.10384200	-4.59297400
C	-3.74782400	-2.04390200	-4.26114600
C	-1.60050000	-2.64778100	-4.56828500
C	-2.87713100	-0.91313700	-4.08717400
N	-1.55985200	-1.29655700	-4.26745700
C	-2.48642400	1.49829000	-4.01881600
C	-2.95576500	2.84713900	-4.17610100
C	-1.87906500	3.61014900	-4.50196900
N	-1.11168500	1.45461600	-4.18261300
C	-0.72817100	2.75017000	-4.48984500
C	1.68356300	2.35162800	-4.55385200
C	3.04748600	2.80309500	-4.60360500
C	3.83285000	1.74204900	-4.27878700
C	2.96284100	0.61296800	-4.09247600
N	1.64316300	1.00266000	-4.24719400
C	0.81543100	-3.04964400	-4.54210600
C	1.96346300	-3.91522900	-4.54200800
C	3.04220700	-3.15240200	-4.22061500
C	2.57587000	-1.80050500	-4.07632000
N	1.20320500	-1.75185800	-4.25323000
C	-3.33488000	0.39410500	-3.88705600
C	-0.49711700	-3.48940300	-4.74544400
C	3.42245800	-0.69712300	-3.92008100
C	0.57777500	3.18995400	-4.72660500
H	-4.82400200	-2.02871400	-4.17968400
H	-3.28978200	-4.10881400	-4.81479600
H	1.95463000	-4.97566700	-4.74305100
H	4.06641300	-3.47963000	-4.12782100
H	4.91018900	1.72342900	-4.21499100
H	3.37097400	3.80670700	-4.83447100
H	-1.87276300	4.66786800	-4.71680600
H	-3.98168900	3.17051100	-4.09354300
Al	0.04280000	-0.13923200	-3.78498100
C	-4.82259200	0.62839100	-3.68614200
C	-5.63347200	0.76895800	-4.99201700
H	-4.96544700	1.52553800	-3.07743400
H	-5.23589700	-0.18700300	-3.08575200
C	-7.12606500	1.01446600	-4.74073200
H	-5.21912400	1.59151400	-5.58861900
H	-5.50457300	-0.13581600	-5.59933700
H	-7.53441100	0.19137000	-4.13674500
H	-7.24831800	1.92226700	-4.13227500
C	-0.73498100	-4.95294900	-5.08077300
C	-0.91793200	-5.86174000	-3.84708000
H	0.09795800	-5.32836100	-5.68228000
H	-1.61371100	-5.04214300	-5.72652100
C	-1.10814500	-7.33818000	-4.21388100
H	-0.05050300	-5.74985700	-3.18457200
H	-1.78268900	-5.51040900	-3.26975600
H	-1.97213300	-7.43939500	-4.88645300
H	-0.23768100	-7.68640700	-4.78845000
C	4.90920100	-0.93415800	-3.71459200
C	5.72468200	-1.07489700	-5.01755500
H	5.04759300	-1.83347200	-3.10723500
H	5.32177200	-0.12080900	-3.11102100
C	7.21596700	-1.32313400	-4.76126300
H	5.31103300	-1.89592200	-5.61669300
H	5.59946500	-0.16910000	-5.62416700
H	7.62364300	-0.50108400	-4.15542100
H	7.33455200	-2.23150200	-4.15291000
C	0.80532900	4.63769800	-5.13205600

C	1.00941600	5.61438700	-3.95606600
H	-0.03989400	4.97927200	-5.73685700
H	1.67120500	4.69427700	-5.79887000
C	1.17788300	7.06846000	-4.41232300
H	0.16109200	5.53279000	-3.26570100
H	1.89179400	5.30373800	-3.38235300
H	2.01976000	7.13535700	-5.11657100
H	0.28765500	7.37858800	-4.97839800
C	-4.16357300	0.53593100	0.10783800
C	-4.12727200	-0.80688200	-0.10780200
C	-2.79701100	0.99133700	0.17977800
C	-2.73928200	-1.19936500	-0.11649300
N	-1.94925000	-0.08666500	0.06517200
C	-0.95484300	-2.90504100	-0.06549600
C	-0.49479300	-4.26760000	0.02005000
C	0.83711300	-4.22630900	0.30133100
N	0.11272400	-2.04942700	0.11858200
C	1.22368900	-2.83875000	0.33773800
C	2.92596400	-1.04919900	0.25388900
C	4.29246200	-0.59997200	0.14961600
C	4.25730400	0.71862500	-0.18585300
C	2.86902800	1.10492800	-0.25172200
N	2.07681100	0.01077800	0.01953300
C	-1.09964400	2.77599200	0.02135300
C	-0.71286300	4.15706200	-0.13189400
C	0.61902300	4.17190100	-0.40697500
C	1.07413900	2.80218600	-0.38049100
N	0.00430800	1.97034000	-0.13794900
C	-2.29473200	-2.52563200	-0.24493300
C	-2.41858800	2.34268800	0.23468300
C	2.42059900	2.41570500	-0.48779700
C	2.54697300	-2.38642600	0.46442000
H	-4.97188100	-1.47065000	-0.21822700
H	-5.04170400	1.15864100	0.18479800
H	-1.37131900	5.00953700	-0.05726200
H	1.23574000	5.04072000	-0.57983900
H	5.10400900	1.36988400	-0.34375100
H	5.17183200	-1.20905200	0.29483600
H	1.49643200	-5.06953100	0.44352000
H	-1.10838000	-5.14966100	-0.08723100
Al	0.04393700	-0.01316400	0.19207800
C	-3.31985100	-3.60876600	-0.54855700
C	-3.97800200	-4.26186700	0.68239300
H	-2.84634400	-4.38904700	-1.15317000
H	-4.09991400	-3.19080700	-1.19204100
C	-5.03365200	-5.30780700	0.30440300
H	-3.20030200	-4.72935800	1.29902900
H	-4.42645500	-3.48421400	1.31154000
H	-5.81378400	-4.83349900	-0.30902000
H	-4.57478000	-6.07571500	-0.33593000
C	-3.49669700	3.39259100	0.45580400
C	-4.14944000	3.94649400	-0.82558100
H	-3.07366900	4.21878700	1.03333300
H	-4.27464400	2.97498300	1.10047200
C	-5.19803200	5.02863600	-0.54107500
H	-3.37210900	4.34527500	-1.48883400
H	-4.61753200	3.11958300	-1.37523500
H	-5.96473300	4.62467600	0.13568200
H	-4.72580000	5.85922900	0.00286000
C	3.45882000	3.48987000	-0.77427300
C	4.02247700	4.18504700	0.48160600
H	3.02595900	4.24584400	-1.43638800
H	4.28319400	3.05476100	-1.34655100
C	5.08630500	5.23923900	0.15319800
H	3.19647100	4.65235300	1.03197600
H	4.43895600	3.43008000	1.15933600
H	5.91573300	4.76445600	-0.39134000
H	4.66232500	5.98407800	-0.53651400
C	3.63372200	-3.41339200	0.74210900
C	4.25146300	-4.05425100	-0.51724100
H	3.22954700	-4.19715000	1.38859200
H	4.42859700	-2.94771600	1.33185500
C	5.31449000	-5.11023900	-0.19177000
H	3.45548300	-4.50200200	-1.12563400
H	4.69593000	-3.26538800	-1.13803100
H	6.10024000	-4.65704300	0.42972800
H	4.86449100	-5.90205300	0.42415500
O	0.08906600	-0.32221600	-1.98911300

C	-5.68619900	-5.98506300	1.51588900
C	-6.74438500	-7.02926900	1.13960800
H	-6.14420000	-5.21820300	2.15639300
H	-4.90697200	-6.46270000	2.12695400
C	-7.39693500	-7.70792700	2.35020600
H	-7.52438600	-6.55231800	0.52856200
H	-6.28638100	-7.79636600	0.49843100
C	-8.45209600	-8.74898000	1.96478300
H	-6.61793600	-8.18476100	2.96047000
H	-7.85508300	-6.94184900	2.99035600
H	-8.89840100	-9.21432300	2.84960100
H	-9.26293000	-8.29519000	1.38347600
H	-8.01636200	-9.54763800	1.35355100
C	5.95385200	-5.73879700	-1.43610100
C	7.01445100	-6.79869800	-1.11453800
H	6.40874100	-4.94656300	-2.04874600
H	5.16799700	-6.18950500	-2.05924300
C	7.65518500	-7.42535400	-2.35903000
H	7.80000600	-6.34878700	-0.49068200
H	6.56107300	-7.59173500	-0.50275700
C	8.71279400	-8.48261100	-2.02831900
H	6.87009100	-7.87534000	-2.98207000
H	8.10862200	-6.63278100	-2.97001400
H	9.15049100	-8.91009200	-2.93626300
H	9.52916000	-8.05452000	-1.43560400
H	8.28176400	-9.30609600	-1.44770000
C	5.63897600	5.95551100	1.39166400
C	6.70490200	7.00944500	1.06820800
H	6.06092800	5.21159700	2.08199500
H	4.80977700	6.43316200	1.93291200
C	7.25472700	7.72771400	2.30668700
H	7.53568100	6.53266400	0.52805600
H	6.28305300	7.75304400	0.37653800
C	8.31799500	8.77897400	1.97504700
H	6.42479800	8.20394500	2.84615900
H	7.67689300	6.98510800	2.99726900
H	8.68947900	9.27269600	2.87886200
H	9.17727600	8.32708600	1.46638300
H	7.91552500	9.55519600	1.31413200
C	-5.87527100	5.57156400	-1.80554700
C	-6.92186800	6.65718100	-1.52675600
H	-6.35230200	4.74065000	-2.34599700
H	-5.10854900	5.97318700	-2.48379000
C	-7.60021200	7.19696200	-2.79187300
H	-7.68832600	6.25684000	-0.84782700
H	-6.44683100	7.48933100	-0.98771700
C	-8.64357500	8.28064500	-2.50428600
H	-6.83410000	7.59726900	-3.47002300
H	-8.07526400	6.36524900	-3.33007700
H	-9.10873100	8.64456700	-3.42618100
H	-9.44190200	7.90025000	-1.85698500
H	-8.19182000	9.14117900	-1.99789900
C	-7.94722900	1.15371800	-6.02857200
C	-9.43992400	1.40150300	-5.77949100
H	-7.82618500	0.24525500	-6.63559800
H	-7.53822400	1.97640100	-6.63213000
C	-10.26267400	1.53997100	-7.06633300
H	-9.84920700	0.57917400	-5.17517300
H	-9.56132100	2.31052100	-5.17297200
C	-11.75186400	1.78778700	-6.80846600
H	-9.85376300	2.36168500	-7.66991500
H	-10.14204500	0.63135300	-7.67182100
H	-12.31065700	1.88191600	-7.74519000
H	-12.19745800	0.96561400	-6.23698400
H	-11.90702300	2.70879200	-6.23506900
C	-1.30645400	-8.24716800	-2.99450700
C	-1.49331000	-9.72594100	-3.35488300
H	-2.17901300	-7.90025500	-2.42252800
H	-0.44350000	-8.14363300	-2.32148700
C	-1.69155600	-10.63462300	-2.13542600
H	-2.35649700	-9.82998500	-4.02791600
H	-0.62114000	-10.07352100	-3.92711100
C	-1.87667300	-12.10962800	-2.50372000
H	-0.82901600	-10.53048700	-1.46321400
H	-2.56332400	-10.28765700	-1.56416900
H	-2.01536700	-12.73063000	-1.61288900
H	-2.75281200	-12.25077900	-3.14681100
H	-1.00546300	-12.49539900	-3.04529900

C	8.04123200	-1.46301200	-6.04641800
C	9.53274100	-1.71311200	-5.79259100
H	7.92354600	-0.55408000	-6.65338500
H	7.63299400	-2.28477200	-6.65175000
C	10.35943400	-1.85213200	-7.07684600
H	9.94126000	-0.89167300	-5.18654700
H	9.65086300	-2.62262600	-5.18615200
C	11.84741800	-2.10229200	-6.81432800
H	9.95124500	-2.67291100	-7.68218300
H	10.24210100	-0.94301700	-7.68222100
H	12.40906400	-2.19673200	-7.74930700
H	12.29239400	-1.28108600	-6.24098100
H	11.99938400	-3.02385000	-6.24095600
C	1.40809200	8.04760700	-3.25443500
C	1.57431600	9.50370200	-3.70586300
H	2.30051900	7.73878200	-2.69127800
H	0.56738800	7.97969400	-2.54947500
C	1.80849500	10.48250100	-2.54864600
H	2.41378600	9.57148400	-4.41271800
H	0.68148100	9.81421700	-4.26742200
C	1.97312400	11.93415400	-3.00815300
H	0.96972500	10.41474300	-1.84258400
H	2.70088200	10.17239700	-1.98813900
H	2.13849100	12.60647400	-2.16016000
H	2.82624700	12.04055200	-3.68786200
H	1.08203700	12.28474900	-3.54123800
O	0.18740300	0.14932000	2.05240200
H	1.08089000	0.50963800	2.15026400
H	0.30629500	-1.25484200	-1.83924600
C	-3.08168800	-2.87251000	3.98168700
C	-3.87316300	-1.77940900	4.14725600
C	-1.71801400	-2.39830300	3.88362000
C	-3.01017900	-0.62038900	4.11478400
N	-1.71083700	-1.02666000	3.93181800
C	-2.61854900	1.82848100	4.11986900
C	-3.08192600	3.19785700	4.11630700
C	-1.99536500	3.98579300	3.89529400
N	-1.26014100	1.80870500	3.92901400
C	-0.84620900	3.10997300	3.81095200
C	1.60760100	2.73104200	3.89836500
C	2.95995100	3.21284600	4.06979300
C	3.73666100	2.13432600	4.36099700
C	2.87893700	0.97197200	4.33424800
N	1.59328900	1.36274500	4.03622900
C	0.73166400	-2.78034700	4.05201500
C	1.87527500	-3.64441200	4.23899000
C	2.94706400	-2.84317200	4.48814300
C	2.48185200	-1.47703400	4.41904800
N	1.13158500	-1.46940800	4.15731600
C	-3.45415900	0.70592600	4.26180900
C	-0.58909300	-3.23851900	3.88484100
C	3.31124200	-0.34717400	4.55758300
C	0.48498200	3.56262400	3.73417900
H	-4.94602200	-1.76908500	4.27247500
H	-3.39875100	-3.90437100	3.96725300
H	1.86686400	-4.72423400	4.21468500
H	3.96112700	-3.15999600	4.68357800
H	4.79728600	2.13763200	4.56553800
H	3.27997200	4.24264100	4.01287100
H	-1.97856400	5.06406900	3.83462400
H	-4.10371000	3.52333500	4.24674900
Al	-0.09652400	0.17096600	4.13682900
C	-4.92131500	0.93744200	4.59910800
C	-5.86661400	1.07697300	3.38996300
H	-5.00989800	1.83170100	5.22329900
H	-5.27589700	0.11695000	5.23018600
C	-7.33569400	1.24800100	3.79500600
H	-5.55136500	1.93844600	2.78679400
H	-5.75745700	0.19881700	2.74175600
H	-7.65134900	0.38133300	4.39356600
H	-7.43273600	2.12065200	4.45687400
C	-0.81832000	-4.74208700	3.83636400
C	-0.98065000	-5.40322600	5.22086500
H	0.00816600	-5.22098500	3.30291500
H	-1.70555100	-4.95777700	3.23361900
C	-1.21828400	-6.91592900	5.14036200
H	-0.08564000	-5.20064100	5.82250100
H	-1.81426300	-4.92510400	5.75050700

H	-2.11413000	-7.11282700	4.53304400
H	-0.38247700	-7.38909300	4.60413500
C	4.76998200	-0.56805100	4.93325200
C	5.72462000	-0.75979800	3.73792900
H	4.84874900	-1.43693800	5.59388700
H	5.12041900	0.27668400	5.53393900
C	7.18885100	-0.92983200	4.15999500
H	5.40328700	-1.63793400	3.16283100
H	5.62826600	0.09596800	3.05815200
H	7.50897200	-0.04488500	4.72874300
H	7.27264800	-1.77859700	4.85410800
C	0.72128300	5.05977600	3.59668800
C	0.78434100	5.81931400	4.93817000
H	-0.06338300	5.49679500	2.97148400
H	1.65111300	5.23382300	3.04591000
C	1.02652700	7.32354700	4.76552100
H	-0.15038900	5.65448600	5.48827400
H	1.57769700	5.38462000	5.55891800
H	1.96257000	7.48158600	4.20950100
H	0.23010200	7.75261500	4.13966000
C	-8.28526200	1.41086100	2.60160000
C	-9.75593100	1.57807100	3.00260000
H	-8.18728000	0.53903500	1.93867900
H	-7.97188700	2.28043200	2.00545200
C	-10.70484000	1.74290300	1.80908200
H	-10.07104900	0.70895300	3.59769600
H	-9.85472400	2.44910800	3.66615700
C	-12.17121900	1.90864300	2.21907800
H	-10.39056800	2.61199800	1.21490900
H	-10.60637000	0.87228900	1.14616100
H	-12.82207000	2.02383900	1.34610600
H	-12.52498700	1.03927000	2.78490600
H	-12.30762300	2.79142000	2.85405600
C	1.09275900	8.08815900	6.09339200
C	1.33413700	9.59318800	5.92547500
H	1.88944000	7.65997800	6.71796400
H	0.15754500	7.93006300	6.64881400
C	1.40075800	10.35722800	7.25357200
H	2.26984600	9.75208900	5.36987900
H	0.53681000	10.02234400	5.30132900
C	1.64170400	11.85947800	7.07746900
H	0.46607200	10.19894700	7.80847900
H	2.19762200	9.92921400	7.87681700
H	1.68392000	12.37536900	8.04235600
H	2.58706100	12.05103000	6.55683800
H	0.84220300	12.32276700	6.48788700
C	8.14552100	-1.14574300	2.98078700
C	9.61149000	-1.31149700	3.39922200
H	8.06050000	-0.29851400	2.28511900
H	7.82821900	-2.03417700	2.41530800
C	10.56761400	-1.53088200	2.22035900
H	9.93068300	-0.42328100	3.96312900
H	9.69685400	-2.15743000	4.09632500
C	12.02918100	-1.69475500	2.64790500
H	10.24929400	-2.41906300	1.65735700
H	10.48265500	-0.68537000	1.52393800
H	12.68537700	-1.84948900	1.78511500
H	12.38721300	-0.80774300	3.18281300
H	12.15216200	-2.55382400	3.31722600
C	-1.38132000	-7.58305100	6.51172200
C	-1.61990600	-9.09594100	6.43560000
H	-2.21681100	-7.11017000	7.04697000
H	-0.48584100	-7.38681100	7.11823500
C	-1.78301000	-9.76253200	7.80714100
H	-2.51594700	-9.29300800	5.82922700
H	-0.78404800	-9.56981100	5.90061000
C	-2.02068600	-11.27321000	7.72255700
H	-0.88792500	-9.56610100	8.41285100
H	-2.61838000	-9.28985500	8.34132100
H	-2.13277500	-11.71842500	8.71659400
H	-2.92899600	-11.50028500	7.15262800
H	-1.18529900	-11.77869700	7.22452500
O	-0.22433400	0.29838000	5.92209700
H	0.19832700	-0.42423000	6.39690100

(d) Tetra Stack

C	5.78973000	-0.31935000	5.42127300
C	5.05377500	-1.45668400	5.53152100
C	6.12928400	-0.16652700	4.03192800
C	4.98465700	-2.04386400	4.22097100
N	5.63901900	-1.23878100	3.30526700
C	4.65226900	-3.96899500	2.75289800
C	4.42884400	-5.37601800	2.56588000
C	4.96060300	-5.70881400	1.35996200
N	5.25817000	-3.43352500	1.62827000
C	5.46226100	-4.49987100	0.76741400
C	6.37576000	-3.21675300	-1.10303700
C	6.79841500	-3.08558800	-2.47081000
C	6.92090200	-1.75600600	-2.72657800
C	6.63996600	-1.06142100	-1.49975300
N	6.28967900	-1.96766600	-0.51310500
C	6.94747000	1.15158300	2.13675800
C	7.45855500	2.36139100	1.55163600
C	7.38483300	2.21747600	0.20152800
C	6.88131800	0.89590500	-0.05560700
N	6.59267500	0.25856500	1.13951200
C	4.43598200	-3.30572400	3.96525900
C	6.78734500	0.95346300	3.51244800
C	6.83698700	0.31302100	-1.32718000
C	6.04112400	-4.43510900	-0.50370100
H	4.63040800	-1.87442600	6.43222500
H	6.05841700	0.36093800	6.21502000
H	7.81495100	3.22360600	2.09442800
H	7.68340700	2.93551700	-0.54678000
H	7.21879700	-1.29550000	-3.65622500
H	6.96170500	-3.89954300	-3.16076200
H	4.99386900	-6.69050600	0.91255300
H	3.96696500	-6.04061000	3.27927700
Al	5.51928900	-1.47136800	1.28740800
C	3.73247300	-4.04236800	5.09267200
C	4.66678100	-4.86670700	6.00386800
H	2.97105000	-4.70340100	4.66946900
H	3.17468900	-3.32238200	5.69824800
C	3.91265500	-5.59861400	7.12052300
H	5.21928900	-5.59240000	5.39374400
H	5.42474500	-4.20489700	6.44149200
H	3.35780800	-4.86731800	7.72583400
H	3.15303300	-6.25765900	6.67561100
C	7.26676800	2.03371500	4.46859800
C	6.20864000	3.11035600	4.78863300
H	8.15626700	2.51722100	4.05454600
H	7.60353700	1.57240000	5.40180900
C	6.73476400	4.20810000	5.72069300
H	5.85109000	3.55509000	3.85142500
H	5.33443900	2.62747700	5.24384700
H	7.10302400	3.75183500	6.65086300
H	7.60663500	4.69260900	5.25799200
C	7.17271900	1.16578000	-2.53927200
C	8.67659100	1.23405300	-2.88051300
H	6.79638900	2.18037300	-2.37882400
H	6.61811300	0.79307200	-3.40512400
C	8.96993800	2.11277300	-4.10240900
H	9.22789000	1.61408100	-2.01118400
H	9.05584600	0.21929400	-3.05520800
H	8.41078400	1.73121200	-4.96889100
H	8.58711800	3.12768500	-3.92150100
C	6.26624600	-5.72501000	-1.27692200
C	5.08356300	-6.16289400	-2.16461500
H	6.50121400	-6.52952400	-0.57380700
H	7.16123300	-5.62232100	-1.89813100
C	5.33563900	-7.49635600	-2.87824700
H	4.17641200	-6.23054600	-1.55181800
H	4.88490800	-5.37974900	-2.90736400
H	6.25484500	-7.42240800	-3.47712400
H	5.52655900	-8.27968000	-2.13059400
C	0.42131700	-2.59131500	3.74195200
C	0.85904400	-1.47068500	4.37697700
C	0.70817800	-2.42334300	2.33890400
C	1.36972900	-0.57950300	3.36478500
N	1.26004900	-1.17949400	2.13003700
C	2.18945400	1.62186000	2.60847300
C	2.50727000	3.00962500	2.82661800

C	2.66853400	3.58355600	1.60222500
N	2.20575700	1.35575100	1.25336100
C	2.50561400	2.54428600	0.61812000
C	2.82565600	1.63544700	-1.65556300
C	3.30162800	1.75024400	-3.01173700
C	3.39090800	0.48857300	-3.51406200
C	2.93117800	-0.41300000	-2.48671100
N	2.58730400	0.30905500	-1.36405100
C	1.12196200	-3.35721000	0.09398300
C	1.19521400	-4.46178000	-0.83014400
C	1.88677600	-4.02832800	-1.91839400
C	2.20510300	-2.63877500	-1.69437600
N	1.74402100	-2.25856000	-0.45363800
C	1.86075300	0.71037200	3.62423600
C	0.56308300	-3.43864400	1.37986400
C	2.80720300	-1.80293000	-2.64918300
C	2.73739900	2.71187100	-0.75647600
H	0.81015000	-1.26205700	5.43532100
H	-0.02891400	-3.46132600	4.19460400
H	0.79249000	-5.45017000	-0.66647400
H	2.12958300	-4.59874400	-2.80194100
H	3.71558700	0.20686700	-4.50457100
H	3.55707700	2.66924600	-3.51699300
H	2.90563700	4.61659400	1.39610600
H	2.57155100	3.49983500	3.78652600
Al	1.76552600	-0.41807100	0.35422800
C	2.00930500	1.15217300	5.07309600
C	0.76625800	1.81845200	5.69421400
H	2.85695100	1.84022300	5.15187500
H	2.29337300	0.28798600	5.68094500
C	0.96422900	2.18515400	7.16989600
H	0.51911700	2.72096700	5.12145300
H	-0.09730300	1.15190800	5.58487800
H	1.20547400	1.27739200	7.74217700
H	1.84001600	2.84342300	7.26882200
C	-0.15450300	-4.71981100	1.77662000
C	0.74071900	-5.82278100	2.37424400
H	-0.68029200	-5.11572400	0.90365700
H	-0.94645900	-4.47806700	2.49067500
C	-0.03653600	-7.10010400	2.71473300
H	1.55237400	-6.05477800	1.67391300
H	1.22684500	-5.43813000	3.28000500
H	-0.85619100	-6.85498600	3.40541900
H	-0.51716400	-7.48733800	1.80488600
C	3.27154100	-2.41787400	-3.96078900
C	2.19838200	-2.46239700	-5.06706600
H	3.63588500	-3.43288300	-3.77647700
H	4.14400200	-1.86991200	-4.32765000
C	2.72464200	-3.04149600	-6.38568100
H	1.34804100	-3.06020900	-4.71584900
H	1.80405000	-1.45231500	-5.23193800
H	3.57156700	-2.43494600	-6.73854700
H	3.13058500	-4.04806800	-6.20663200
C	3.01371700	4.11494500	-1.27393000
C	4.49704100	4.53595300	-1.24361900
H	2.42088200	4.83180100	-0.69949900
H	2.63980100	4.20129000	-2.29820100
C	4.72146100	5.96748400	-1.74513600
H	4.88444700	4.43356700	-0.22206100
H	5.07981700	3.83671600	-1.85718500
H	4.31634800	6.06465000	-2.76270900
H	4.14251900	6.66603500	-1.12414200
O	3.88926200	-0.77733600	0.93624700
C	-0.25571300	2.86969000	7.79869700
C	-0.06283500	3.23437500	9.27566700
H	-1.13126300	2.21281400	7.69828900
H	-0.49395100	3.77939400	7.22910800
C	-1.28262000	3.91954800	9.90390600
H	0.17512400	2.32511500	9.84649300
H	0.81307700	3.89151800	9.37665600
C	-1.08163500	4.28029400	11.37871300
H	-1.52017700	4.82795300	9.33386000
H	-2.15738900	3.26287200	9.80348000
H	-1.96870000	4.76698500	11.79674800
H	-0.87762000	3.38765300	11.98107200
H	-0.23535800	4.96472400	11.50780200
C	6.19503900	6.39265300	-1.74548100
C	6.42163700	7.82459700	-2.24536300

H	6.77264500	5.69596000	-2.37071600
H	6.60129000	6.29473700	-0.72843700
C	7.89527800	8.24922000	-2.24965700
H	6.01378000	7.92310200	-3.26153500
H	5.84587100	8.52238800	-1.62065900
C	8.11123000	9.68017700	-2.75105000
H	8.30256300	8.15180600	-1.23395600
H	8.47048500	7.55190200	-2.87412800
H	9.17156300	9.95261400	-2.74217200
H	7.74595200	9.79978500	-3.77733300
H	7.57689200	10.40411400	-2.12544000
C	1.66119500	-3.11479700	-7.48839000
C	2.18498500	-3.68794600	-8.81079300
H	1.25260700	-2.10974000	-7.66470500
H	0.81768600	-3.72639100	-7.13731200
C	1.12077800	-3.76289500	-9.91255800
H	3.02812000	-3.07639100	-9.16339100
H	2.59462500	-4.69313700	-8.63467400
C	1.65222800	-4.33594700	-11.22966000
H	0.27879700	-4.37424300	-9.56055600
H	0.71179100	-2.75876400	-10.08867700
H	0.86958000	-4.37596400	-11.99409700
H	2.47218500	-3.72576200	-11.62539200
H	2.03567500	-5.35361100	-11.09328700
C	0.83425600	-8.20015000	3.33432000
C	0.06054700	-9.48020800	3.67275500
H	1.31024700	-7.81385700	4.24768400
H	1.65539500	-8.44451500	2.64498500
C	0.93073500	-10.57880700	4.29551500
H	-0.76159200	-9.23627700	4.36064000
H	-0.41488800	-9.86860900	2.76074500
C	0.14938200	-11.85284700	4.63013900
H	1.75191700	-10.82297800	3.60769700
H	1.40541200	-10.19056100	5.20711700
H	0.79747200	-12.61634900	5.07247600
H	-0.65681500	-11.64735700	5.34343800
H	-0.30790200	-12.28448300	3.73260100
C	4.82609700	-6.42432900	8.03484700
C	4.07343500	-7.15682900	9.15226100
H	5.58522900	-5.76531400	8.47962000
H	5.37939700	-7.15605100	7.42921400
C	4.98506600	-7.98391800	10.06706000
H	3.52042800	-6.42500700	9.75851900
H	3.31353500	-7.81547600	8.70766500
C	4.22539500	-8.71154100	11.18018600
H	5.53683100	-8.71559200	9.46132200
H	5.74410800	-7.32574300	10.51134700
H	4.90305000	-9.29162800	11.81484200
H	3.69162900	-8.00346900	11.82425600
H	3.48328600	-9.40393700	10.76671600
C	5.68454900	5.27301400	6.06049600
C	6.20643900	6.37481600	6.99066700
H	4.81452300	4.78775000	6.52582700
H	5.31534100	5.72778700	5.13027900
C	5.15585700	7.43864700	7.33219200
H	6.57673900	5.92009600	7.92069800
H	7.07601700	6.86115400	6.52570300
C	5.68537400	8.53528400	8.26090200
H	4.78601700	7.89279900	6.40285300
H	4.28746300	6.95262900	7.79737500
H	4.91279300	9.27768300	8.48528100
H	6.03000800	8.11614700	9.21307000
H	6.53221600	9.06344900	7.80815100
C	10.46066100	2.18905000	-4.45481000
C	10.75770000	3.06803800	-5.67576600
H	10.84241100	1.17430800	-4.63637800
H	11.01885900	2.57060400	-3.58810400
C	12.24811500	3.14413900	-6.02923600
H	10.19924700	2.68692300	-6.54284100
H	10.37659200	4.08330900	-5.49433400
C	12.53630300	4.02364700	-7.24947100
H	12.80580700	3.52534800	-5.16299600
H	12.62852800	2.12972700	-6.21082100
H	13.60696300	4.05713700	-7.47543700
H	12.02041000	3.64733900	-8.14017100
H	12.19923400	5.05334300	-7.08453500
C	4.17596700	-7.93163100	-3.78277800
C	4.42159300	-9.26641800	-4.49667100

H	3.98806000	-7.14923800	-4.53217100
H	3.25675900	-8.00393100	-3.18421000
C	3.26290900	-9.70018000	-5.40297700
H	5.34158600	-9.19497600	-5.09448000
H	4.60837500	-10.04981200	-3.74815900
C	3.51603300	-11.03395800	-6.11183400
H	2.34392300	-9.77163200	-4.80545800
H	3.07677100	-8.91750600	-6.15096700
H	2.67201000	-11.31491200	-6.74985900
H	4.40894400	-10.98315200	-6.74525900
H	3.67054100	-11.84370600	-5.38966600
O	0.03984500	0.04271200	-0.25037300
H	0.19197600	0.10666800	-1.20430200
H	3.97321900	0.16685900	1.13678100
C	-2.22508000	1.98867500	3.53127500
C	-2.85031500	0.78948300	3.67534700
C	-1.82041800	2.09432200	2.14901400
C	-2.80096300	0.12769100	2.39403100
N	-2.16192800	0.93875500	1.48564700
C	-3.10072400	-1.84751300	0.93844400
C	-3.38507500	-3.24820300	0.74099900
C	-2.92602300	-3.57957800	-0.49630700
N	-2.52136000	-1.33610700	-0.19885700
C	-2.40165800	-2.37445200	-1.09223600
C	-1.59706200	-1.07559300	-3.03476200
C	-1.30472800	-0.93340600	-4.43954600
C	-1.17870300	0.39882700	-4.69130600
C	-1.34106800	1.08808100	-3.43499100
N	-1.59886700	0.16839600	-2.44136400
C	-1.09275500	3.42400000	0.19057500
C	-0.70587600	4.66569900	-0.43498700
C	-0.74405100	4.46667200	-1.78097900
C	-1.11737900	3.08996300	-1.99772400
N	-1.33325900	2.47969000	-0.78191500
C	-3.30202300	-1.16199100	2.14832000
C	-1.26947100	3.25322800	1.57485600
C	-1.16823900	2.47131700	-3.25946200
C	-1.90665300	-2.29251600	-2.40505300
H	-3.29367900	0.39132400	4.57586500
H	-2.08569900	2.74249100	4.29107200
H	-0.46509000	5.58526700	0.07746400
H	-0.52324700	5.19144000	-2.55005600
H	-0.97226800	0.86242100	-5.64451700
H	-1.23690800	-1.73849000	-5.15588700
H	-2.97334400	-4.54935000	-0.96927600
H	-3.85282800	-3.90533500	1.45815100
Al	-2.00838600	0.57423600	-0.48647600
C	-4.06004100	-1.86533200	3.26470500
C	-3.19708500	-2.67863600	4.24790800
H	-4.81299300	-2.52373400	2.82351600
H	-4.63756900	-1.12314500	3.82168600
C	-4.02024500	-3.31758500	5.37310900
H	-2.66466100	-3.46348200	3.69511700
H	-2.42040700	-2.03206300	4.67399100
H	-4.54738800	-2.53020000	5.93077700
H	-4.80518300	-3.95153900	4.93597200
C	-0.94475000	4.42624200	2.48777900
C	-2.11169400	5.40761300	2.71484800
H	-0.08839200	4.97276500	2.08270400
H	-0.60317200	4.04859500	3.45622300
C	-1.72756000	6.59640000	3.60398500
H	-2.47850300	5.76742800	1.74624500
H	-2.95229500	4.86554400	3.16516000
H	-1.34955500	6.22594600	4.56849500
H	-0.89029700	7.14144600	3.14351600
C	-0.95426800	3.32968400	-4.49710400
C	0.51442300	3.48437700	-4.93842900
H	-1.38018200	4.32222000	-4.32458000
H	-1.53928400	2.91658100	-5.32314800
C	0.66960200	4.31945300	-6.21510600
H	1.08569200	3.94929600	-4.12448300
H	0.95735900	2.49153200	-5.08577000
H	0.10238400	3.84726800	-7.03011900
H	0.21010700	5.30689200	-6.06338900
C	-1.74573000	-3.58146600	-3.19861900
C	-2.97955000	-4.02667100	-4.00666700
H	-1.45720100	-4.38523500	-2.51485600
H	-0.89591600	-3.47640100	-3.88048900

C	-2.76077100	-5.35647800	-4.73827900
H	-3.84532700	-4.10268600	-3.33898000
H	-3.23409500	-3.24502200	-4.73288900
H	-1.88343100	-5.27230200	-5.39684000
H	-2.51169600	-6.13909100	-4.00648500
C	-7.29343600	-0.63767600	2.11636400
C	-6.92310500	0.51403300	2.73803800
C	-6.88046000	-0.52543400	0.73520500
C	-6.32310400	1.36481400	1.73221200
N	-6.29011000	0.69650300	0.53659800
C	-5.61444900	3.60788900	0.92963300
C	-5.49429100	5.03758600	1.11151800
C	-5.32530700	5.58619700	-0.12185600
N	-5.46393400	3.30672800	-0.40306500
C	-5.30260100	4.49785800	-1.07250400
C	-5.02106900	3.57404500	-3.36071500
C	-4.70196400	3.71291000	-4.76325600
C	-4.63317500	2.45744900	-5.28460300
C	-4.94697700	1.53502600	-4.21645200
N	-5.16480500	2.24044700	-3.05762000
C	-6.52428500	-1.46448500	-1.53400600
C	-6.61311000	-2.52917500	-2.50794400
C	-6.02115900	-2.08040300	-3.64655900
C	-5.59342000	-0.72073600	-3.39511400
N	-5.87952300	-0.38921300	-2.09472700
C	-5.95608800	2.70619700	1.95409200
C	-7.04677100	-1.53527400	-0.23006700
C	-5.10275800	0.14632400	-4.38937800
C	-5.13083000	4.65123200	-2.45973200
H	-7.08086900	0.76981300	3.77552000
H	-7.78611500	-1.48765100	2.56566700
H	-7.06272400	-3.49864400	-2.35037000
H	-5.92312000	-2.61590100	-4.57880600
H	-4.41653300	2.19369800	-6.30941700
H	-4.53354100	4.64212300	-5.28789500
H	-5.21995900	6.63597400	-0.35367300
H	-5.56438800	5.56746100	2.04981600
Al	-5.79908900	1.45610000	-1.27111800
C	-6.06863100	3.24885500	3.37161100
C	-7.46109300	3.80814500	3.73054400
H	-5.32131300	4.03428700	3.52255400
H	-5.80073700	2.46324600	4.08478700
C	-7.53856200	4.35248800	5.16194600
H	-7.72360900	4.59999200	3.01799500
H	-8.21141900	3.01995100	3.59193300
H	-7.27066300	3.55549700	5.87128700
H	-6.78113400	5.13920700	5.29516500
C	-7.85471100	-2.76938800	0.15075500
C	-7.04353800	-3.94127500	0.73661700
H	-8.39900300	-3.12445600	-0.72954800
H	-8.63197700	-2.48410600	0.86592300
C	-7.90488900	-5.17386000	1.03739300
H	-6.23892500	-4.20825600	0.04059800
H	-6.54768400	-3.60878700	1.65796600
H	-8.71622200	-4.89357900	1.72463600
H	-8.39746400	-5.50787400	0.11275100
C	-4.85976400	-0.42587700	-5.77852000
C	-6.08750600	-0.36769500	-6.71102100
H	-4.52759100	-1.46478600	-5.69390000
H	-4.02375600	0.10009700	-6.24918700
C	-5.81662700	-0.95702700	-8.10049600
H	-6.92141100	-0.90191000	-6.23853500
H	-6.41472400	0.67504300	-6.80897300
H	-4.97644800	-0.42183300	-8.56707500
H	-5.48626400	-2.00114300	-7.99624200
C	-5.03565400	6.06578700	-3.01551900
C	-3.61068900	6.65176300	-3.06355800
H	-5.67048600	6.73001900	-2.42129500
H	-5.46417000	6.09154900	-4.02214600
C	-3.57331300	8.09750300	-3.57298300
H	-3.16323800	6.59571800	-2.06351100
H	-2.98639700	6.01900300	-3.70762300
H	-4.03709400	8.14616100	-4.56890800
H	-4.19488700	8.72909600	-2.92186400
O	-3.77956800	1.20061600	-0.82129800
C	-8.91820500	4.91283900	5.52920400
C	-8.99959400	5.45951800	6.95961600
H	-9.67480300	4.12652600	5.39728900

H	-9.18551700	5.70920600	4.82033800
C	-10.37914700	6.02016300	7.32643600
H	-8.73289700	4.66300700	7.66950200
H	-8.24294700	6.24656700	7.09210300
C	-10.45197800	6.56439100	8.75625900
H	-10.64555000	6.81609400	6.61780600
H	-11.13503900	5.23404700	7.19502000
H	-11.44801900	6.95654200	8.98678700
H	-10.22489300	5.78243600	9.49006200
H	-9.73186500	7.37666700	8.90836300
C	-2.15860400	8.68554500	-3.64548500
C	-2.11820900	10.13343800	-4.14900600
H	-1.53844800	8.05705800	-4.30129100
H	-1.69358100	8.63456400	-2.65045600
C	-0.70343500	10.72089100	-4.22269100
H	-2.58355900	10.18534200	-5.14382000
H	-2.73728600	10.76305700	-3.49387200
C	-0.67302700	12.16720800	-4.72578100
H	-0.23859300	10.66940800	-3.22851100
H	-0.08490500	10.09241200	-4.87812800
H	0.34936500	12.55718200	-4.76716900
H	-1.09950800	12.24547900	-5.73232900
H	-1.25391200	12.82664100	-4.07089500
C	-7.03174800	-0.90270300	-9.03470800
C	-6.76637800	-1.49160500	-10.42552100
H	-7.36116000	0.14077000	-9.13929900
H	-7.87092000	-1.43767800	-8.56805700
C	-7.98203600	-1.43634000	-11.35887100
H	-5.92703400	-0.95668100	-10.89330100
H	-6.43758300	-2.53592300	-10.32190200
C	-7.70929300	-2.02649200	-12.74566700
H	-8.82035200	-1.97098800	-10.89196600
H	-8.31021000	-0.39321400	-11.46309000
H	-8.59525100	-1.97207000	-13.38683700
H	-6.89902800	-1.48979600	-13.25251500
H	-7.41318900	-3.07960900	-12.67703000
C	-7.11408700	-6.34223400	1.63893100
C	-7.97171100	-7.57789800	1.93771400
H	-6.62431700	-6.00919500	2.56568700
H	-6.30217600	-6.62199000	0.95219200
C	-7.18070200	-8.74593800	2.53939300
H	-8.78367000	-7.29918100	2.62464900
H	-8.46196900	-7.91210200	1.01205900
C	-8.04520300	-9.97535000	2.83452600
H	-6.36984900	-9.02506700	1.85256300
H	-6.69116900	-8.41221900	3.46467200
H	-7.45241200	-10.79045100	3.26266800
H	-8.84401600	-9.73630100	3.54579200
H	-8.52032600	-10.35369100	1.92223000
C	-3.18113200	-4.15272300	6.34809800
C	-4.00047900	-4.79078000	7.47649300
H	-2.39500900	-3.51959700	6.78444200
H	-2.65780400	-4.94326300	5.79027700
C	-3.16112400	-5.62745700	8.44984100
H	-4.52371400	-4.00207900	8.03586700
H	-4.78708800	-5.42348700	7.04103200
C	-3.98770800	-6.26096600	9.57289800
H	-2.63847100	-6.41584000	7.89084200
H	-2.37564500	-4.99483300	8.88572600
H	-3.36072600	-6.85077500	10.24959300
H	-4.49450200	-5.49574400	10.17177200
H	-4.75920300	-6.92675400	9.16970600
C	-2.88580200	7.56957400	3.85614400
C	-2.50458800	8.76688400	4.73526000
H	-3.71850700	7.02636500	4.32542200
H	-3.26853700	7.93372700	2.89237600
C	-3.66455000	9.73768400	4.98819500
H	-2.12077400	8.40310300	5.69950500
H	-1.67266100	9.31208900	4.26616100
C	-3.27565200	10.93149400	5.86532400
H	-4.04821900	10.10062000	4.02506200
H	-4.49506500	9.19350400	5.45796800
H	-4.12432100	11.60410800	6.02688900
H	-2.92103900	10.60211200	6.84877100
H	-2.47122900	11.51649100	5.40478800
C	2.12759000	4.50301400	-6.65419800
C	2.28577600	5.33275300	-7.93410400
H	2.58851500	3.51581200	-6.80278900

H	2.69306200	4.98153100	-5.84107400
C	3.74389700	5.51841700	-8.37159900
H	1.72202600	4.85510500	-8.74821800
H	1.82401500	6.31955500	-7.78666600
C	3.89174500	6.34775300	-9.65071300
H	4.30710000	5.99655500	-7.55835300
H	4.20522600	4.53227700	-8.51945300
H	4.94261900	6.46227800	-9.93605800
H	3.36875800	5.87679800	-10.49079500
H	3.47083500	7.35151800	-9.52287900
C	-3.97139100	-5.80601700	-5.56571000
C	-3.75849700	-7.13829200	-6.29463000
H	-4.21686600	-5.02620300	-6.30083000
H	-4.84862900	-5.88781800	-4.90858200
C	-4.96867400	-7.58636800	-7.12332600
H	-2.88038100	-7.05730800	-6.95173100
H	-3.51414300	-7.91950200	-5.56020500
C	-4.74796400	-8.91788400	-7.84725300
H	-5.84575000	-7.66746200	-6.46704100
H	-5.21238000	-6.80652900	-7.85754300
H	-5.62856200	-9.20920300	-8.42899800
H	-3.89878700	-8.85783700	-8.53769400
H	-4.53741300	-9.72538400	-7.13661700
O	-7.53631900	1.80918700	-1.57754900
H	-7.70665600	2.16699000	-2.45470500
H	-3.66667700	2.14691700	-0.65201900

(e) Penta-Stack

C	-0.26537100	2.85737400	3.13028900
C	0.04754700	3.66486800	2.08125700
C	-0.30781200	1.50462200	2.62831800
C	0.16000700	2.82265100	0.91549300
N	-0.06685300	1.51413500	1.27360200
C	0.33048200	2.48434700	-1.52530200
C	0.32277600	2.98564500	-2.87776700
C	0.06708800	1.92856700	-3.69531600
N	0.13355400	1.12318200	-1.54261900
C	-0.03477400	0.75804200	-2.85812300
C	-0.13373600	-1.68890800	-2.53206400
C	-0.12142700	-3.04300900	-3.02618200
C	0.09775500	-3.85910900	-1.95848700
C	0.17246400	-3.02026700	-0.78823100
N	0.03248700	-1.70116000	-1.16334400
C	-0.29599500	-0.94652100	2.96803700
C	-0.27967800	-2.11810000	3.80984200
C	-0.00546800	-3.18448800	3.00977700
C	0.11163000	-2.68634500	1.66117400
N	-0.05401000	-1.31834100	1.66405400
C	0.41329900	3.29719100	-0.38252200
C	-0.46672000	0.36693900	3.43821300
C	0.27456300	-3.50170300	0.52767300
C	-0.22883700	-0.54629100	-3.34335700
H	0.17378500	4.73715000	2.10093900
H	-0.42047600	3.15703600	4.15542800
H	-0.42881700	-2.13163500	4.87927300
H	0.08998100	-4.21580600	3.31389300
H	0.17664700	-4.93601600	-1.97010200
H	-0.23202600	-3.34028100	-4.05820800
H	-0.01361800	1.94409300	-4.77213000
H	0.46670800	4.01417900	-3.17103400
Al	0.12145300	-0.07354400	0.05637600
C	0.73737900	4.77338400	-0.56091300
C	-0.47375400	5.70678600	-0.74790900
H	1.41080800	4.88936700	-1.41443700
H	1.32204100	5.11251300	0.29819100
C	-0.07273200	7.18185600	-0.87104500
H	-1.02701200	5.40264000	-1.64571400
H	-1.16846000	5.57697800	0.09059000
H	0.47478800	7.48686400	0.03233100
H	0.63524600	7.29929800	-1.70410800
C	-0.75247200	0.56632800	4.91939800
C	0.49813100	0.69175100	5.81162700
H	-1.36953200	-0.26086900	5.28086500
H	-1.37455900	1.45651800	5.05149200
C	0.15704400	0.83270600	7.29996700
H	1.14533100	-0.17968000	5.65629400

H	1.08513800	1.55909400	5.48521300
H	-0.50332400	1.70073500	7.44357600
H	-0.42358300	-0.04280600	7.62593800
C	0.49258900	-4.99348300	0.73182400
C	-0.79415100	-5.83709900	0.82259300
H	1.08504200	-5.14961000	1.63796400
H	1.11920300	-5.37615000	-0.07809400
C	-0.51398200	-7.33614100	0.98317700
H	-1.39539600	-5.48348500	1.67007200
H	-1.40689300	-5.66533100	-0.07099500
H	0.08211800	-7.68869600	0.12919800
H	0.11247800	-7.49712000	1.87242100
C	-0.51795800	-0.73034800	-4.82652800
C	0.71612200	-0.89908800	-5.73302300
H	-1.10756900	0.12007100	-5.18081200
H	-1.17347000	-1.59697400	-4.95680000
C	0.34976400	-1.02253900	-7.21697300
H	1.39735100	-0.05356100	-5.58334500
H	1.27452600	-1.78902000	-5.41762700
H	-0.34291000	-1.86616300	-7.35413400
H	-0.20420300	-0.12594900	-7.53186000
C	4.32775400	4.10423600	-0.42428900
C	4.06080900	4.07005000	0.90951700
C	4.34152800	2.73852800	-0.88995900
C	3.95774400	2.68089000	1.28686700
N	4.13203500	1.89350200	0.17382900
C	3.89629100	0.88404900	2.98303200
C	3.94933800	0.41947600	4.34727000
C	4.19828700	-0.91848000	4.30694900
N	4.06758700	-0.18465400	2.13092400
C	4.25195300	-1.30086000	2.91709300
C	4.23708600	-2.99698500	1.11436200
C	4.18529900	-4.36177800	0.64889400
C	3.93270900	-4.32139700	-0.68804500
C	3.86384700	-2.93041400	-1.06677900
N	4.04586500	-2.14536000	0.04907000
C	4.25042300	1.04985400	-2.69407500
C	4.22533100	0.66512800	-4.08473200
C	3.95544900	-0.66715500	-4.12702800
C	3.85004100	-1.12366800	-2.76094400
N	4.00972700	-0.05462900	-1.91096700
C	3.77424100	2.23190300	2.60603300
C	4.46172700	2.36233900	-2.23858500
C	3.72217700	-2.47353000	-2.38885000
C	4.38877500	-2.62298200	2.46146300
H	3.97699800	4.91429600	1.57790200
H	4.47911800	4.98161800	-1.03440100
H	4.38362900	1.32343000	-4.92596300
H	3.87220500	-1.28407300	-5.00865700
H	3.83396400	-5.16521000	-1.35479300
H	4.31015900	-5.24392000	1.25862700
H	4.31448900	-1.58282000	5.15040100
H	3.84597700	1.03421100	5.22885500
Al	4.13539300	-0.10408700	0.09633100
C	3.47905200	3.26329000	3.68610300
C	4.70759200	3.87436800	4.38617000
H	2.83101800	2.80993700	4.44280600
H	2.87874700	4.06818400	3.25210000
C	4.32775900	4.93709700	5.42448800
H	5.27991000	3.07443800	4.87161800
H	5.37995800	4.30490900	3.63547300
H	3.75847600	5.74005300	4.93330500
H	3.64461900	4.49655600	6.16597900
C	4.80453700	3.43427800	-3.26312600
C	3.60495800	4.14573500	-3.91662200
H	5.42739400	2.99033500	-4.04382400
H	5.44794300	4.18242100	-2.79269800
C	4.02987800	5.17482500	-4.97133000
H	2.93925500	3.40044000	-4.36831300
H	3.01322600	4.64408700	-3.13791700
H	4.70565400	5.91047800	-4.51155000
H	4.62012500	4.67329300	-5.75173900
C	3.52607200	-3.50905800	-3.48635200
C	4.83013900	-4.06983700	-4.08700700
H	2.92180200	-3.07603400	-4.28915100
H	2.92380600	-4.33537300	-3.09838200
C	4.58233500	-5.13716500	-5.15968700
H	5.41114200	-3.24383600	-4.51506600

H	5.45305100	-4.48256700	-3.28473300
H	4.00665400	-5.96697400	-4.72358700
H	3.94772000	-4.71771100	-5.95453900
C	4.65465200	-3.71180700	3.49046700
C	3.39583400	-4.35230400	4.10679700
H	5.28058000	-3.30311800	4.28823500
H	5.26519700	-4.49632100	3.03427800
C	3.72356800	-5.40954200	5.16807700
H	2.76395600	-3.56864300	4.54262700
H	2.79766100	-4.80837100	3.30742400
H	4.36813300	-6.18295400	4.72569000
H	4.31718600	-4.94821600	5.97048400
O	2.03594300	-0.13184800	0.27575400
C	5.53465600	5.54513200	6.14968200
C	5.15668900	6.61020900	7.18626300
H	6.21800200	5.98495600	5.40965000
H	6.10135200	4.74336400	6.64448100
C	6.36282300	7.21762400	7.91306500
H	4.59036100	7.41317200	6.69235700
H	4.47247700	6.17053900	7.92654300
C	5.97572000	8.27975700	8.94643300
H	6.92853400	6.41578000	8.40666300
H	7.04589000	7.65776600	7.17398300
H	6.85742600	8.69332200	9.44682600
H	5.43909100	9.11235500	8.47712900
H	5.32110300	7.86081900	9.71936900
C	2.48204400	-6.07396300	5.77580900
C	2.80672500	-7.12907100	6.84026000
H	1.89133500	-6.53990900	4.97346600
H	1.83617000	-5.30091400	6.21656900
C	1.56524200	-7.79468200	7.44661700
H	3.45294500	-7.90208800	6.40020600
H	3.39678700	-6.66482600	7.64344800
C	1.89922200	-8.84699200	8.50821500
H	0.91994600	-7.02227300	7.88724300
H	0.97575800	-8.25890000	6.64399200
H	0.99339600	-9.30295600	8.92124300
H	2.51450400	-9.65117800	8.08892800
H	2.45853500	-8.40577000	9.34103100
C	5.87120100	-5.68889200	-5.78162200
C	5.63013400	-6.76295600	-6.84923000
H	6.50785600	-6.10278600	-4.98706700
H	6.44220800	-4.86024000	-6.22435400
C	6.92002500	-7.31216800	-7.47103300
H	5.05979500	-7.59313800	-6.40767900
H	4.99308500	-6.34926600	-7.64452700
C	6.67115300	-8.38449300	-8.53589300
H	7.48956500	-6.48316800	-7.91258300
H	7.55618500	-7.72570800	-6.67676800
H	7.61029000	-8.75541500	-8.95930800
H	6.13411200	-9.24292400	-8.11619700
H	6.06726300	-7.99070300	-9.36154600
C	2.85171100	5.90952300	-5.62266300
C	3.27535500	6.93654500	-6.67979100
H	2.26380100	6.41518100	-4.84260300
H	2.17515700	5.17441900	-6.08205900
C	2.09797100	7.67159900	-7.33200800
H	3.95136700	7.67223300	-6.22094500
H	3.86379100	6.43270100	-7.45997200
C	2.53150800	8.69515700	-8.38571500
H	1.42298500	6.93657700	-7.79170000
H	1.51000300	8.17551100	-6.55254900
H	1.66976000	9.20206800	-8.83233800
H	3.17914400	9.46358100	-7.94847500
H	3.09199500	8.21585100	-9.19641000
C	-1.26329800	8.12514900	-1.08328500
C	-0.86583200	9.60159900	-1.20081400
H	-1.97328400	8.00601800	-0.25205100
H	-1.80680400	7.82389300	-1.99092500
C	-2.05606600	10.54453700	-1.41583900
H	-0.32354500	9.90472700	-0.29377300
H	-0.15489500	9.72131800	-2.03078600
C	-1.64938600	12.01663600	-1.53113100
H	-2.59729800	10.24226900	-2.32297000
H	-2.76642700	10.42492900	-0.58621400
H	-2.51985100	12.66299600	-1.68386000
H	-1.13683000	12.35816500	-0.62468000
H	-0.96658900	12.17415300	-2.37371400

C	1.39117300	0.98336200	8.19803100
C	1.05593800	1.11629100	9.68852700
H	1.96700400	1.86312800	7.87667900
H	2.05370500	0.11881400	8.05065700
C	2.29090300	1.26824200	10.58509400
H	0.39288600	1.98102100	9.83667600
H	0.48062700	0.23653600	10.01159600
C	1.94771500	1.39977100	12.07189400
H	2.95306600	0.40441000	10.43723100
H	2.86533000	2.14729200	10.26282600
H	2.84936100	1.50632400	12.68367800
H	1.31536500	2.27570700	12.25693200
H	1.40367700	0.51929800	12.43266100
C	-1.78399400	-8.18809700	1.09918100
C	-1.50695400	-9.68846000	1.25362200
H	-2.41253400	-8.02482900	0.21183300
H	-2.37650200	-7.83972500	1.95796300
C	-2.77675900	-10.54022100	1.37221100
H	-0.91556500	-10.03846000	0.39545800
H	-0.87747300	-9.85220300	2.13996900
C	-2.48992400	-12.03688400	1.52548700
H	-3.36732500	-10.19102500	2.23040200
H	-3.40557500	-10.37689400	0.48616000
H	-3.41501700	-12.61676300	1.60758600
H	-1.93081400	-12.42313300	0.66571300
H	-1.89228600	-12.23586800	2.42227500
C	1.56504000	-1.21307500	-8.13300800
C	1.20393500	-1.33055500	-9.61875000
H	2.11522800	-2.11268900	-7.82193400
H	2.25878200	-0.37200100	-7.99361600
C	2.41982800	-1.52213200	-10.53361300
H	0.50963700	-2.17176600	-9.75906700
H	0.65434200	-0.43084500	-9.93149100
C	2.05078800	-1.63767600	-12.01553900
H	3.11325000	-0.68188300	-10.39345500
H	2.96845800	-2.42120700	-10.22179400
H	2.93928200	-1.77324100	-12.64067900
H	1.38621200	-2.49096300	-12.19338300
H	1.53203800	-0.73809500	-12.36628000
O	6.05388700	-0.25956000	0.02186400
H	6.13335600	-1.15580000	-0.33454700
H	2.07990300	-0.42206200	1.19789900
C	7.96918600	2.94799000	3.15964900
C	8.19458000	3.74034400	2.07827900
C	7.83431600	1.59190700	2.67164000
C	8.16651800	2.88653300	0.91181700
N	7.92094300	1.59294300	1.30238500
C	8.28434200	2.50653200	-1.53818700
C	8.38746700	2.97434600	-2.90258800
C	8.19159500	1.89690500	-3.70926900
N	8.05126900	1.15510300	-1.53903600
C	8.01525000	0.74787700	-2.84647900
C	8.03968400	-1.71046900	-2.47777800
C	8.24157500	-3.05959700	-2.95874500
C	8.45583300	-3.84577600	-1.86950100
C	8.35242100	-2.99644400	-0.70444500
N	8.08171600	-1.70835100	-1.10424800
C	7.92852500	-0.86456500	3.04672600
C	8.07354900	-2.01325500	3.91284900
C	8.33643000	-3.08517600	3.11622900
C	8.31815400	-2.61430300	1.75001100
N	8.06810700	-1.26233300	1.73923600
C	8.38961600	3.33180000	-0.40361700
C	7.77833500	0.45859100	3.50460600
C	8.50345200	-3.43776800	0.62224400
C	7.94787700	-0.58013400	-3.31048300
H	8.35984000	4.80785500	2.08078000
H	7.93771000	3.25819700	4.19317000
H	8.01397000	-2.00822800	4.99137400
H	8.50922700	-4.10255500	3.43586000
H	8.65892300	-4.90675000	-1.86776800
H	8.25594100	-3.37088300	-3.99266900
H	8.20511600	1.88529400	-4.78928400
H	8.56786700	3.99303400	-3.21372800
Al	8.11394100	-0.02450200	0.10245800
C	8.79183500	4.78709700	-0.60777100
C	7.62880200	5.77943400	-0.80011200
H	9.46275400	4.85717300	-1.46920500

H	9.39446100	5.11350900	0.24515400
C	8.09914500	7.23247000	-0.93865600
H	7.05804700	5.49400300	-1.69341000
H	6.93341100	5.69023900	0.04341200
H	8.66274600	7.51998500	-0.03926900
H	8.80982400	7.30800300	-1.77439900
C	7.69452900	0.68179300	5.00771900
C	9.06402100	0.79934000	5.70835200
H	7.12280800	-0.13054600	5.46629500
H	7.11316000	1.58569900	5.21181800
C	8.94883200	1.03110500	7.21977300
H	9.64455000	-0.11192100	5.51713600
H	9.63118600	1.61947400	5.25037300
H	8.36339600	1.94373800	7.40568800
H	8.37492900	0.20927400	7.67284500
C	8.87258700	-4.89739400	0.85130900
C	7.67537100	-5.86097400	0.96984900
H	9.48595400	-4.98095300	1.75373500
H	9.52003000	-5.23752200	0.03736900
C	8.09774300	-7.32534200	1.13845400
H	7.05599500	-5.55672200	1.82343800
H	7.03745700	-5.75507500	0.08374000
H	8.71211700	-7.62850600	0.27826300
H	8.74903400	-7.41887800	2.01963100
C	7.91383900	-0.80560500	-4.81533300
C	9.30425800	-0.88069900	-5.47990600
H	7.33010000	-0.01058700	-5.28941600
H	7.36580800	-1.72742400	-5.03439900
C	9.23413100	-1.11195400	-6.99411500
H	9.85265700	0.04614800	-5.27104900
H	9.88378400	-1.68414700	-5.00845600
H	8.68133900	-2.04132300	-7.19672200
H	8.64718900	-0.30666300	-7.46011500
C	6.95420400	8.22913600	-1.15785400
C	7.42076800	9.68367200	-1.29323200
H	6.24223700	8.15294900	-0.32338700
H	6.39350200	7.94372200	-2.05993300
C	6.27613900	10.67961000	-1.51666600
H	7.97937700	9.97142400	-0.39096900
H	8.13436500	9.76025800	-2.12620100
C	6.75140300	12.12951900	-1.65028100
H	5.71859100	10.39273300	-2.41886100
H	5.56313000	10.60344700	-0.68424100
H	5.91221400	12.81495700	-1.80874000
H	7.28226700	12.45694900	-0.74906600
H	7.43876300	12.24463700	-2.49614500
C	10.61095500	-1.18686000	-7.66574700
C	10.54531300	-1.41815600	-9.18025700
H	11.19714000	-1.99190800	-7.20050600
H	11.16279900	-0.25795500	-7.46386700
C	11.92219100	-1.49208800	-9.85170600
H	9.99415300	-2.34801700	-9.38297000
H	9.95887200	-0.61296000	-9.64636500
C	11.84793800	-1.72321200	-11.36398300
H	12.47269300	-0.56316700	-9.64999000
H	12.50796400	-2.29668700	-9.38680000
H	12.84560800	-1.77115200	-11.81281400
H	11.33362500	-2.66284200	-11.59653300
H	11.29823800	-0.91603800	-11.86178500
C	6.91512700	-8.29169300	1.27993300
C	7.33327000	-9.75805600	1.44289300
H	6.26219400	-8.19634900	0.40047800
H	6.30393600	-7.99174300	2.14368300
C	6.15038000	-10.72363500	1.58615900
H	7.94355400	-10.05978300	0.57950600
H	7.98687300	-9.85416000	2.32182300
C	6.57723200	-12.18568900	1.74779900
H	5.54092600	-10.42282000	2.44940900
H	5.49750500	-10.62800000	0.70770600
H	5.71150300	-12.84880600	1.84753100
H	7.15886000	-12.52633800	0.88358400
H	7.20265400	-12.31965900	2.63780400
C	10.30554000	1.14910000	7.92521700
C	10.19571400	1.38127800	9.43705100
H	10.87836500	1.97073800	7.47246800
H	10.89019900	0.23692400	7.73969300
C	11.55294300	1.49910600	10.14138100
H	9.61138500	2.29407700	9.62362200

H	9.62319500	0.55928400	9.89095300
C	11.43502800	1.73063200	11.65081600
H	12.13664700	0.58729600	9.95545100
H	12.12460700	2.32061700	9.68865600
H	12.41962000	1.81019100	12.12336800
H	10.88657600	2.65438000	11.86845000
H	10.89862300	0.90779800	12.13733900
O	9.91248100	0.06735500	0.06837800
H	10.33064000	-0.37624500	0.81331900
C	-8.77495900	3.18305700	2.77254200
C	-8.34385200	3.96620600	1.74876300
C	-8.77385200	1.82528400	2.29781700
C	-8.13050600	3.10402200	0.61816900
N	-8.38729800	1.79013300	0.96833300
C	-7.92384300	2.74421900	-1.79016200
C	-7.97785300	3.23915700	-3.13805600
C	-8.30505200	2.18922000	-3.93698900
N	-8.14989700	1.37766400	-1.77941900
C	-8.39693800	1.02719400	-3.09699200
C	-8.60315500	-1.38352500	-2.74250300
C	-8.69670200	-2.73676600	-3.21849600
C	-8.47351100	-3.55227300	-2.15391600
C	-8.30723800	-2.70846500	-1.00199100
N	-8.37204400	-1.37725300	-1.37783100
C	-8.87104900	-0.59459200	2.66273000
C	-8.97519200	-1.75318100	3.50771400
C	-8.65742100	-2.83504600	2.74780000
C	-8.41034200	-2.35718200	1.41483900
N	-8.51949300	-0.97703600	1.37891200
C	-7.82779800	3.57095000	-0.66598800
C	-9.04853200	0.71932800	3.10940400
C	-8.23293400	-3.19481400	0.30782500
C	-8.67158200	-0.25919800	-3.57116700
H	-8.22064400	5.03849000	1.75556700
H	-9.05017500	3.50409900	3.76561800
H	-9.24102400	-1.74984300	4.55383100
H	-8.63282000	-3.86695100	3.06281600
H	-8.46757600	-4.63164300	-2.14948800
H	-8.88709500	-3.03434200	-4.23849300
H	-8.45742400	2.20784200	-5.00535800
H	-7.83180300	4.26496700	-3.43862700
Al	-7.90241100	0.18366900	-0.18318300
C	-7.55603600	5.05292500	-0.86265400
C	-8.81955700	5.91472800	-1.07170100
H	-6.88668000	5.18531200	-1.71724200
H	-6.99436200	5.43160500	-0.00394500
C	-8.49999100	7.40086900	-1.27305000
H	-9.37649900	5.53592800	-1.93798600
H	-9.48817900	5.79395400	-0.21012100
H	-7.93458900	7.77310500	-0.40653800
H	-7.83219700	7.51609200	-2.13911000
C	-9.47145100	0.95182700	4.55102500
C	-8.29633000	1.07557900	5.54348500
H	-10.12813900	0.13849600	4.87293800
H	-10.08793000	1.85377800	4.61005100
C	-8.75550300	1.26249000	6.99429700
H	-7.66117700	0.18435000	5.46397800
H	-7.66431600	1.92174200	5.24472300
H	-9.40209500	2.14927100	7.06135200
H	-9.38317700	0.40985500	7.29091900
C	-8.12146000	-4.69386000	0.53039800
C	-9.47344700	-5.43691500	0.58334600
H	-7.57527500	-4.87880800	1.46007800
H	-7.49648700	-5.12809800	-0.25511500
C	-9.31628000	-6.94411500	0.81763700
H	-10.09392400	-5.00231000	1.37718100
H	-10.01982200	-5.26327900	-0.35226700
H	-8.68975800	-7.37366600	0.02264700
H	-8.76763700	-7.11188700	1.75580800
C	-9.00650400	-0.44532600	-5.04277700
C	-7.78980700	-0.68859200	-5.95870000
H	-9.55228100	0.43252300	-5.40101700
H	-9.70829900	-1.27782600	-5.15125500
C	-8.17312500	-0.81009700	-7.43830600
H	-7.06666600	0.12501000	-5.82380800
H	-7.27557600	-1.60265900	-5.63528800
H	-8.90816100	-1.61871300	-7.56021300
H	-8.68278900	0.10979300	-7.75933400

C	-3.79589200	4.22005400	-0.55040000
C	-4.10617600	4.17497800	0.77337400
C	-3.73418200	2.85767800	-1.01826800
C	-4.18538200	2.78407800	1.14523700
N	-3.94614400	2.00034000	0.03811500
C	-4.33852900	0.97869600	2.82026600
C	-4.39710200	0.49670200	4.17614300
C	-4.16923600	-0.84540400	4.13715300
N	-4.12026100	-0.08375900	1.96519600
C	-4.02208200	-1.21387800	2.75235400
C	-4.04362100	-2.88721500	0.93712600
C	-4.19608800	-4.24003200	0.46225400
C	-4.44891300	-4.17063100	-0.87314400
C	-4.41321800	-2.77712500	-1.24210800
N	-4.15832400	-2.01321000	-0.12322700
C	-3.86009200	1.19317600	-2.83323500
C	-3.98184700	0.83725600	-4.22529600
C	-4.32273100	-0.47874900	-4.27381100
C	-4.36743100	-0.95702500	-2.91313600
N	-4.09893700	0.08474800	-2.05319800
C	-4.43446700	2.32941400	2.45029700
C	-3.61468000	2.49595300	-2.36971900
C	-4.56444300	-2.30074900	-2.55492200
C	-3.91878000	-2.53632500	2.29206900
H	-4.24121800	5.01489700	1.43842300
H	-3.65237600	5.10230100	-1.15503900
H	-3.84940100	1.50631100	-5.06238700
H	-4.49881600	-1.07093200	-5.15885200
H	-4.61833200	-4.99903000	-1.54465600
H	-4.14130300	-5.13266100	1.06660900
H	-4.13586500	-1.52236300	4.97767800
H	-4.56138100	1.10173700	5.05524500
Al	-3.88785200	0.00615500	-0.05607400
C	-4.79373500	3.35196400	3.51887500
C	-3.60182500	3.96620400	4.27839600
H	-5.47397100	2.88983400	4.24111200
H	-5.37772900	4.15532200	3.06014400
C	-4.03151100	5.02692600	5.29896900
H	-3.05154800	3.16633100	4.78953100
H	-2.89665900	4.40020100	3.55971900
H	-4.57744800	5.82982700	4.78231800
H	-4.74811600	4.58431200	6.00646600
C	-3.29675300	3.57871100	-3.38970200
C	-4.51775300	4.29776600	-3.99600800
H	-2.69990100	3.14204000	-4.19498000
H	-2.63948200	4.32109100	-2.92870200
C	-4.12837800	5.34573700	-5.04565700
H	-5.19504500	3.55789400	-4.43966600
H	-5.08662500	4.77988100	-3.19066200
H	-3.43931000	6.07455000	-4.59507200
H	-3.56327700	4.85908900	-5.85352800
C	-4.87914600	-3.30503000	-3.65336300
C	-3.64407000	-3.94618800	-4.31733700
H	-5.48617800	-2.82037300	-4.42371400
H	-5.51941400	-4.09379100	-3.24851500
C	-4.01349700	-4.98777900	-5.38024900
H	-3.03257300	-3.15692900	-4.77245100
H	-3.01322400	-4.40462000	-3.54604300
H	-4.62060100	-5.78027400	-4.91873300
H	-4.65720700	-4.52114200	-6.14039800
C	-3.78199400	-3.64768800	3.32107100
C	-5.11867300	-4.21026400	3.84590600
H	-3.18543800	-3.28535400	4.16265500
H	-3.19439500	-4.46528200	2.89362000
C	-4.93309100	-5.29925300	4.90925600
H	-5.72378900	-3.39068600	4.25348800
H	-5.69206500	-4.61496800	3.00188700
H	-4.31543300	-6.11024200	4.49719000
H	-4.36339500	-4.88882800	5.75532200
O	-6.12348200	0.05200800	0.10130900
C	-2.85982600	5.63415800	6.08042100
C	-3.28605000	6.69893300	7.09837700
H	-2.14239600	6.07441500	5.37352100
H	-2.31732200	4.83192600	6.60094700
C	-2.11463000	7.30527600	7.88079000
H	-3.82812500	7.50216400	6.57863400
H	-4.00423800	6.25916900	7.80549100
C	-2.54936000	8.36767800	8.89463100

H	-1.57344600	6.50297300	8.40044200
H	-1.39738400	7.74486100	7.17448900
H	-1.69159900	8.78006200	9.43556900
H	-3.06230000	9.20082700	8.40068400
H	-3.23974700	7.94937200	9.63600200
C	-6.25466100	-5.88236600	5.42475600
C	-6.07348200	-6.96698700	6.49358500
H	-6.82072500	-6.29942300	4.57885700
H	-6.87421400	-5.07084600	5.83291300
C	-7.39508200	-7.55076300	7.00797100
H	-5.45365400	-7.77847200	6.08623100
H	-5.50785500	-6.55150000	7.33981900
C	-7.20413900	-8.63246600	8.07524600
H	-8.01418200	-6.73973900	7.41537600
H	-7.95992300	-7.96661300	6.16234600
H	-8.16439900	-9.02821500	8.42141000
H	-6.61871900	-9.47350100	7.68677900
H	-6.67301800	-8.23783700	8.94883400
C	-2.79655100	-5.61897000	-6.06786100
C	-3.16386200	-6.66403300	-7.12835600
H	-2.15167900	-6.08336200	-5.30843500
H	-2.19273600	-4.82692700	-6.53363900
C	-1.94755000	-7.29465300	-7.81748600
H	-3.76752800	-7.45681400	-6.66333900
H	-3.80951500	-6.19978700	-7.88782500
C	-2.32390700	-8.33672100	-8.87485800
H	-1.34466300	-6.50270600	-8.28219300
H	-1.30290200	-7.75898200	-7.05893400
H	-1.43515000	-8.76729200	-9.34724000
H	-2.89743000	-9.15994400	-8.43373100
H	-2.93934100	-7.89408000	-9.66640600
C	-5.32868800	6.08907600	-5.64478200
C	-4.94119500	7.13434400	-6.69790000
H	-5.89022800	6.58113800	-4.83698800
H	-6.01938600	5.36080400	-6.09385900
C	-6.14064300	7.87988900	-7.29577900
H	-4.24961400	7.86199000	-6.24974500
H	-4.38029300	6.64396700	-7.50634100
C	-5.74323300	8.92138300	-8.34602500
H	-6.83134600	7.15272500	-7.74432400
H	-6.70078300	8.37017700	-6.48774400
H	-6.62004300	9.43530100	-8.75302100
H	-5.08069100	9.68241500	-7.91845300
H	-5.21193000	8.45623900	-9.18407300
C	-9.74719200	8.27019100	-1.47565100
C	-9.43201000	9.75728600	-1.67766500
H	-10.41360500	8.15474200	-0.60907600
H	-10.31189900	7.89759100	-2.34202800
C	-10.67918000	10.62722700	-1.87729100
H	-8.86577300	10.12994100	-0.81195800
H	-8.76702800	9.87361600	-2.54551900
C	-10.35614900	12.11074600	-2.07822800
H	-11.24459100	10.25500500	-2.74243300
H	-11.34310800	10.51114400	-1.00986000
H	-11.26587600	12.70359700	-2.21746300
H	-9.82149500	12.52075300	-1.21386200
H	-9.72249300	12.26278300	-2.95938800
C	-7.59471900	1.40627900	7.98645600
C	-8.04856300	1.59103000	9.43955100
H	-6.96946100	2.26097800	7.69053800
H	-6.94758200	0.52025800	7.91800400
C	-6.88796100	1.73681400	10.43144700
H	-8.69667800	2.47649900	9.50810800
H	-8.67321200	0.73636400	9.73651500
C	-7.34979400	1.92046500	11.88006000
H	-6.24062700	0.85194500	10.36283300
H	-6.26434600	2.59114600	10.13483700
H	-6.49928200	2.02141600	12.56182300
H	-7.97020900	2.81742300	11.98772300
H	-7.94634100	1.06527600	12.21745000
C	-10.65272900	-7.69468300	0.87108300
C	-10.50058900	-9.20257500	1.10498700
H	-11.20041900	-7.52622600	-0.06702000
H	-11.27797600	-7.26498100	1.66653900
C	-11.83703100	-9.95306900	1.15776700
H	-9.87519300	-9.63282900	0.30956300
H	-9.95331300	-9.37177100	2.04349500
C	-11.67693100	-11.45817900	1.39132800

H	-12.46143900	-9.52335700	1.95285200
H	-12.38345000	-9.78401400	0.21992500
H	-12.64724600	-11.96404700	1.42362400
H	-11.08654900	-11.92269500	0.59335500
H	-11.16532900	-11.66016900	2.33924700
C	-6.97476700	-1.07235500	-8.35893600
C	-7.35320600	-1.19012200	-9.84034800
H	-6.46794700	-1.99459400	-8.04004100
H	-6.23912300	-0.26485000	-8.23535300
C	-6.15536100	-1.45327500	-10.76119400
H	-8.08937100	-1.99728700	-9.96432900
H	-7.85982800	-0.26821100	-10.16012900
C	-6.54211500	-1.56859900	-12.23856500
H	-5.42000000	-0.64672200	-10.63702000
H	-5.64976200	-2.37482800	-10.44202400
H	-5.66652100	-1.75605100	-12.86828600
H	-7.24965600	-2.38975300	-12.40028700
H	-7.01813200	-0.64872200	-12.59674500
O	-2.01400700	-0.21812000	-0.09522300
H	-1.94989500	-1.11412700	-0.45577800
H	-6.04041700	-0.19943400	1.03318700

(f) Hexa-Stack

C	10.82191800	4.21944400	-0.32286600
C	10.42242200	4.19036900	0.97590400
C	10.81020500	2.86291000	-0.80099100
C	10.21842300	2.81043300	1.32424400
N	10.44736200	2.00409800	0.22314800
C	10.05743200	1.02298300	2.98294400
C	10.15044900	0.56615600	4.34203100
C	10.47079000	-0.75416700	4.29947800
N	10.25442800	-0.03742400	2.11368300
C	10.51972700	-1.13621300	2.91536200
C	10.66378700	-2.79713400	1.12645500
C	10.73472600	-4.15127800	0.64924600
C	10.48138300	-4.11932400	-0.68602300
C	10.31820600	-2.73892300	-1.05281800
N	10.41410000	-1.93648600	0.07165500
C	10.86951400	1.20179400	-2.59960500
C	10.94746200	0.82554900	-3.98506000
C	10.62424700	-0.49288300	-4.06248800
C	10.39977700	-0.95253200	-2.71922900
N	10.52948900	0.10145100	-1.83015200
C	9.95196000	2.37141600	2.62605300
C	11.05734100	2.50659100	-2.13107100
C	10.22086400	-2.29768000	-2.37691200
C	10.77157900	-2.43871900	2.47379600
H	10.31566700	5.03130000	1.64410300
H	11.08300000	5.09090000	-0.90372900
H	11.20078500	1.48124700	-4.80414200
H	10.58197900	-1.10126100	-4.95282600
H	10.45357600	-4.95833000	-1.36451500
H	10.93225200	-5.02316200	1.25423100
H	10.64544600	-1.40823700	5.14009500
H	10.03342500	1.17928400	5.22198200
Al	9.95640400	0.03115800	0.12835800
C	9.71077300	3.40416300	3.71423600
C	10.99328600	3.94316300	4.38328600
H	9.06140300	2.97274200	4.48114200
H	9.13869900	4.23805800	3.29750200
C	10.70355700	4.96770500	5.48674300
H	11.56383700	3.10354200	4.79991500
H	11.63940900	4.39430300	3.61978200
H	10.12746100	5.80452400	5.06615700
H	10.05527000	4.50954500	6.24778500
C	11.46190600	3.58860100	-3.11933100
C	10.27440200	4.30497300	-3.79595700
H	12.10473800	3.15447900	-3.89048500
H	12.08771800	4.32990400	-2.61356200
C	10.71579400	5.35580000	-4.82141900
H	9.63012300	3.55914100	-4.27833700
H	9.65629700	4.77994500	-3.02319600
H	11.36912100	6.09221000	-4.33174500
H	11.33215400	4.87440100	-5.59426800
C	10.08052000	-3.32657300	-3.48632800
C	11.41780600	-3.88372000	-4.01942600
H	9.52116400	-2.88508100	-4.31641700

H	9.45615800	-4.15214300	-3.13314500
C	11.22897100	-4.91229700	-5.14085800
H	12.03765300	-3.05319800	-4.38010000
H	11.97766700	-4.33755800	-3.19200200
H	10.60335800	-5.73948500	-4.77556100
H	10.66634900	-4.45238600	-5.96624600
C	11.12579100	-3.50740600	3.49594500
C	9.91878200	-4.26104600	4.09064600
H	11.69722300	-3.05151500	4.30982700
H	11.80998000	-4.22998600	3.04091600
C	10.32430800	-5.28765500	5.15486200
H	9.21378700	-3.53677500	4.51676000
H	9.37672700	-4.76487800	3.28014700
H	11.04159600	-5.99932600	4.72128000
H	10.86159800	-4.77731700	5.96717800
C	5.91933100	2.94915100	3.04682000
C	6.19933400	3.74801700	1.98209700
C	5.84716000	1.59602300	2.55450700
C	6.25070500	2.90147300	0.81598100
N	6.02194600	1.59496500	1.18851200
C	6.36074200	2.55394400	-1.62470400
C	6.39735200	3.03367200	-2.98224300
C	6.15756500	1.96645600	-3.79341400
N	6.14408600	1.18990900	-1.62585400
C	6.02410600	0.80822100	-2.94736100
C	6.04345500	-1.63489500	-2.59235900
C	6.18131200	-2.98503500	-3.07848900
C	6.44451300	-3.77457700	-2.00173100
C	6.43170800	-2.92534600	-0.83657700
N	6.17830000	-1.62535100	-1.21976100
C	5.98133400	-0.83978200	2.91356500
C	6.12351600	-1.99279200	3.76776900
C	6.43282600	-3.04823600	2.96752600
C	6.44005800	-2.56385400	1.60835300
N	6.17885800	-1.21139600	1.60333000
C	6.47370000	3.37058500	-0.48863900
C	5.75286500	0.46480300	3.38055000
C	6.60419700	-3.38368800	0.47998200
C	5.90932900	-0.50830400	-3.42134100
H	6.33303000	4.81944200	1.99080800
H	5.80353400	3.25377400	4.07555200
H	6.02530800	-1.99859700	4.84303500
H	6.61273600	-4.06622500	3.27769800
H	6.60792200	-4.84184700	-2.00543300
H	6.11062900	-3.29666700	-4.10944900
H	6.10790100	1.96964300	-4.87202700
H	6.55622100	4.05731300	-3.28673700
Al	5.93641100	-0.01410700	0.00721300
C	6.82451300	4.83911800	-0.68141500
C	5.62424000	5.78846100	-0.86252300
H	7.48856900	4.93779000	-1.54576800
H	7.42284900	5.17691200	0.16979800
C	6.04203700	7.25799600	-0.99326200
H	5.06035200	5.48659900	-1.75401000
H	4.93434000	5.66790300	-0.01897800
H	6.59877600	7.56031300	-0.09426500
H	6.74568500	7.36494100	-1.83191000
C	5.48431500	0.66514900	4.86445000
C	6.73653600	0.84110200	5.74540000
H	4.89958300	-0.18060900	5.23582400
H	4.83135500	1.53242200	4.99558200
C	6.40055300	0.99144600	7.23408200
H	7.40924900	-0.01256900	5.59874500
H	7.29360200	1.72327200	5.40486600
H	5.71710000	1.84198600	7.37015200
H	5.84658900	0.10448100	7.57367800
C	6.90548600	-4.85950400	0.69346700
C	5.65842400	-5.75615000	0.82920800
H	7.52930300	-4.97806100	1.58423000
H	7.52365500	-5.22529900	-0.13106100
C	6.00314800	-7.24225900	0.98296300
H	5.07234400	-5.42326000	1.69505200
H	5.00991100	-5.61056500	-0.04333300
H	6.58385800	-7.57452900	0.11008300
H	6.66389400	-7.37658900	1.85196500
C	5.74336200	-0.72244000	-4.91782700
C	7.06380600	-0.83159400	-5.70707500
H	5.14004100	0.09050700	-5.33101600

H	5.15042200	-1.62543900	-5.08947600
C	6.84531300	-1.00882100	-7.21436000
H	7.67470100	0.06066000	-5.52101500
H	7.64396100	-1.67822200	-5.31785300
H	6.22079300	-1.89656700	-7.39034800
H	6.26971100	-0.15583400	-7.60163600
O	8.17055900	0.11560900	-0.13166600
C	4.85985600	8.21292000	-1.20082300
C	5.27265500	9.68442100	-1.32645100
H	4.15479600	8.10395100	-0.36458900
H	4.30718700	7.91362800	-2.10305200
C	4.09053700	10.63875900	-1.53592700
H	5.82487300	9.98529600	-0.42446600
H	5.97829100	9.79401800	-2.16266000
C	4.51164900	12.10586200	-1.66125500
H	3.53840500	10.33790800	-2.43663800
H	3.38638600	10.53030900	-0.69947300
H	3.64635800	12.75988900	-1.80918600
H	5.03527000	12.44638700	-0.76069700
H	5.18831500	12.25283900	-2.51080200
C	8.14917000	-1.14242900	-8.01083500
C	7.93331500	-1.31378700	-9.51937600
H	8.72115300	-2.00029100	-7.62772200
H	8.77571800	-0.25670100	-7.83227000
C	9.23669400	-1.44980500	-10.31605500
H	7.30586700	-2.19865900	-9.69820100
H	7.36212000	-0.45665100	-9.90376400
C	9.01082300	-1.62014300	-11.82123200
H	9.86336100	-0.56522600	-10.13766400
H	9.80711500	-2.30674500	-9.93223200
H	9.95871600	-1.71451900	-12.36075800
H	8.41677700	-2.51615000	-12.03392500
H	8.47362100	-0.76211500	-12.24095200
C	4.77024900	-8.14069500	1.14184300
C	5.10800800	-9.62922400	1.28915400
H	4.10778300	-8.00282000	0.27559900
H	4.19383300	-7.81179800	2.01856800
C	3.87438100	-10.52630400	1.44872000
H	5.68452300	-9.95960100	0.41306700
H	5.77031500	-9.76795100	2.15589800
C	4.22027700	-12.01089600	1.59628000
H	3.29789900	-10.19579400	2.32341100
H	3.21353900	-10.38879300	0.58173300
H	3.31958900	-12.62305000	1.70758200
H	4.76642000	-12.37969300	0.72057700
H	4.85130400	-12.18540100	2.47522900
C	7.63480300	1.18762700	8.12315600
C	7.30374500	1.33133000	9.61362200
H	8.18478200	2.07928100	7.78781300
H	8.32014400	0.33902100	7.98440600
C	8.53850400	1.52801300	10.50170700
H	6.61854000	2.17961400	9.75336200
H	6.75381200	0.44097000	9.95054900
C	8.19843000	1.66973800	11.98827000
H	9.22295200	0.68006900	10.36193800
H	9.08753200	2.41821300	10.16531700
H	9.09958000	1.80834200	12.59425400
H	7.54369500	2.53062400	12.16474300
H	7.67981500	0.77992300	12.36274800
C	11.96928800	5.51155100	6.16088900
C	11.68346800	6.53175400	7.26952300
H	12.61524800	5.97280100	5.40040400
H	12.54573000	4.67378500	6.57825100
C	12.94890700	7.07534000	7.94427100
H	11.10700000	7.37018200	6.85281200
H	11.03750800	6.07045000	8.03036700
C	12.65508300	8.09230200	9.05106100
H	13.52477700	6.23744400	8.36020900
H	13.59361000	7.53717200	7.18415900
H	13.57730600	8.46049100	9.51189800
H	12.11069900	8.95893100	8.65904500
H	12.04149300	7.64940200	9.84379600
C	9.54365700	6.08459400	-5.49024700
C	9.98072800	7.13384800	-6.51964100
H	8.92968000	6.56877700	-4.71719300
H	8.88979400	5.34767100	-5.97804500
C	8.80927500	7.86262800	-7.18942500
H	10.63440300	7.87120700	-6.03200700

H	10.59542700	6.65038900	-7.29244500
C	9.25502800	8.90801900	-8.21606000
H	8.15645300	7.12575100	-7.67687400
H	8.19547600	8.34616800	-6.41727800
H	8.39717900	9.40937400	-8.67549400
H	9.88043400	9.67863300	-7.75125600
H	9.84145600	8.44911500	-9.02014100
C	12.54955100	-5.47574200	-5.68022700
C	12.36414300	-6.50578200	-6.80103800
H	13.11163600	-5.93473600	-4.85455600
H	13.17371100	-4.64842400	-6.04666000
C	13.68415800	-7.06996500	-7.34081500
H	11.73992100	-7.33348700	-6.43471100
H	11.80204900	-6.04714000	-7.62725700
C	13.49010500	-8.09817300	-8.45914300
H	14.30733900	-6.24302700	-7.70755500
H	14.24554800	-7.52823200	-6.51527300
H	14.44928900	-8.48107900	-8.82214600
H	12.90033500	-8.95442200	-8.11259000
H	12.96259400	-7.65966900	-9.31385800
C	9.13637900	-6.06063100	5.74114600
C	9.53851200	-7.08577300	6.80842500
H	8.60177800	-6.57394900	4.92887600
H	8.41847600	-5.34908400	6.17340500
C	8.35175800	-7.86079900	7.39408900
H	10.25742200	-7.79677800	6.37664700
H	10.07258700	-6.57315400	7.62132200
C	8.76284300	-8.88195200	8.45887700
H	7.63372200	-7.15033800	7.82583300
H	7.81856500	-8.37325400	6.58176900
H	7.89482500	-9.41754500	8.85639700
H	9.45343400	-9.62707000	8.04789000
H	9.26723400	-8.39512200	9.30126700
O	4.05868800	-0.20335700	-0.07992300
H	3.98976700	-1.10164100	-0.43297000
H	8.06863000	0.51208400	-1.00975400
C	2.27912900	4.23373200	-0.59469700
C	1.99939000	4.20330800	0.73683900
C	2.31869500	2.86734800	-1.05399500
C	1.90299500	2.81745500	1.12050400
N	2.09795800	2.02235900	0.01177500
C	1.82309600	1.02382400	2.81552400
C	1.88289700	0.56698400	4.18307300
C	2.14285400	-0.76834700	4.14721900
N	1.99759800	-0.04485600	1.96861600
C	2.18704600	-1.15447800	2.75727200
C	2.15802600	-2.85383400	0.96517500
C	2.12542100	-4.21649800	0.49913500
C	1.87956900	-4.18012500	-0.83924100
C	1.79794600	-2.79325300	-1.22128800
N	1.95662000	-2.00249600	-0.10213700
C	2.29459800	1.16798000	-2.85121100
C	2.26386300	0.78476400	-4.24315100
C	1.97565700	-0.54392400	-4.28622300
C	1.86813100	-0.99927400	-2.92034000
N	2.05720800	0.06278900	-2.06706700
C	1.70480700	2.37161200	2.43799000
C	2.46672600	2.48719300	-2.39894400
C	1.69179000	-2.34285800	-2.54799300
C	2.32794700	-2.47784800	2.30783300
H	1.88629100	5.05120900	1.39526800
H	2.41692800	5.11166300	-1.20705400
H	2.41244000	1.44503600	-5.08468000
H	1.86719800	-1.15784400	-5.16793800
H	1.78729300	-5.02529000	-1.50376300
H	2.25073200	-5.09711700	1.11086300
H	2.25729100	-1.43222600	4.99140500
H	1.77086700	1.18564200	5.06119400
Al	1.91320000	0.01456700	-0.03805400
C	1.40073700	3.40436500	3.51367700
C	2.62454800	4.02249800	4.21658200
H	0.74397500	2.95418700	4.26291800
H	0.80602900	4.20913300	3.07213800
C	2.23889000	5.09769400	5.23984100
H	3.19225600	3.22766400	4.71722900
H	3.30167200	4.44670800	3.46552000
H	1.67789500	5.89652600	4.73351700
H	1.54676900	4.66770300	5.97806300

C	2.74602500	3.57468100	-3.42593400
C	1.49356400	4.22288500	-4.04777500
H	3.37412500	3.16704100	-4.22294600
H	3.35550400	4.35612200	-2.96305800
C	1.83201200	5.36281100	-5.01595300
H	0.91473200	3.45290800	-4.57288400
H	0.83974900	4.59226000	-3.24878600
H	2.40388500	6.13610300	-4.48195900
H	2.50010300	4.98739300	-5.80499700
C	1.47131500	-3.37537400	-3.64320600
C	2.75809700	-3.96463500	-4.25349100
H	0.86328000	-2.93321200	-4.43686100
H	0.86011300	-4.19106900	-3.24781000
C	2.47963100	-5.04268100	-5.30788400
H	3.34489200	-3.15380400	-4.70379600
H	3.38465600	-4.37826900	-3.45378200
H	1.90072400	-5.85816700	-4.85069100
H	1.83764400	-4.62546100	-6.09703100
C	2.64348800	-3.56350500	3.32711700
C	1.42524700	-4.24428200	3.97986400
H	3.28246100	-3.14426300	4.10962500
H	3.25883000	-4.33052600	2.84727500
C	1.82062000	-5.37759300	4.93429600
H	0.84232400	-3.49197800	4.52567200
H	0.75904800	-4.62723400	3.19810800
H	2.39538100	-6.13530700	4.38137500
H	2.50265500	-4.98696900	5.70376600
C	3.44055600	5.70955300	5.97041800
C	3.05565800	6.78783400	6.99069500
H	4.13359000	6.13912700	5.23267200
H	3.99896700	4.91134900	6.48123900
C	4.25605800	7.39932100	7.72353600
H	2.49768700	7.58653900	6.48137400
H	2.36197500	6.35893200	7.72791000
C	3.86094600	8.47447200	8.74025000
H	4.81347300	6.60115400	8.23284600
H	4.94875500	7.82900200	6.98686000
H	4.73835200	8.89090700	9.24570100
H	3.33233100	9.30301200	8.25541500
H	3.19637400	8.06644900	9.51020200
C	0.62272800	-6.05060000	5.61567700
C	1.01489100	-7.18909800	6.56525600
H	-0.06071900	-6.43827500	4.84703000
H	0.05185800	-5.29441800	6.17349900
C	-0.18288400	-7.86013400	7.24867500
H	1.58456500	-7.94699100	6.00813600
H	1.69968800	-6.80203700	7.33372200
C	0.21770500	-8.99641800	8.19418300
H	-0.75140300	-7.10337900	7.80605500
H	-0.86697800	-8.24656300	6.48106900
H	-0.65840900	-9.45367200	8.66520500
H	0.75708900	-9.78623100	7.65883500
H	0.87367700	-8.63411300	8.99404400
C	3.75089400	-5.61987400	-5.94277400
C	3.47860100	-6.70452400	-6.99206100
H	4.39491000	-6.03291200	-5.15302900
H	4.32576700	-4.80479700	-6.40668000
C	4.75078800	-7.27928200	-7.62709800
H	2.90457400	-7.52044000	-6.53008800
H	2.83428600	-6.29252300	-7.78185500
C	4.46978600	-8.36192500	-8.67333900
H	5.32413500	-6.46382800	-8.08918800
H	5.39421200	-7.69142900	-6.83759800
H	5.39661300	-8.75130100	-9.10707600
H	3.92817300	-9.20693800	-8.23337400
H	3.85768300	-7.97064100	-9.49382500
C	0.59752500	6.00403100	-5.66179300
C	0.93118800	7.15363700	-6.62026800
H	-0.07353300	6.37240000	-4.87302300
H	0.03138200	5.23321400	-6.20423800
C	-0.30380200	7.79353400	-7.26632500
H	1.49674400	7.92561600	-6.07852600
H	1.60294300	6.78607700	-7.40953300
C	0.03798100	8.94224900	-8.21985100
H	-0.86793200	7.02288100	-7.80895200
H	-0.97512800	8.15971900	-6.47779900
H	-0.86364500	9.37674700	-8.66346700
H	0.57151400	9.74511000	-7.69823900

H	0.67955700	8.59953600	-9.03976500
O	-0.00920600	0.18949700	-0.12462300
H	-0.07038800	1.07763700	-0.50417800
C	-1.91989000	3.12240000	-3.02990200
C	-2.19072100	3.86734500	-1.92307800
C	-1.85170600	1.74431900	-2.61081700
C	-2.25509100	2.96080000	-0.80373100
N	-2.04116100	1.67350400	-1.24654800
C	-2.32432700	2.47772800	1.62175700
C	-2.27725400	2.90185400	3.00027200
C	-2.01644500	1.79812900	3.75103300
N	-2.13567100	1.11641000	1.55792000
C	-1.93888800	0.67703900	2.84632800
C	-1.81822800	-1.74461500	2.37147600
C	-1.83698900	-3.12505600	2.79053700
C	-2.06934900	-3.87724400	1.68112900
C	-2.14855300	-2.97056800	0.56162000
N	-1.99052800	-1.67952400	1.00904600
C	-1.83714200	-0.68475800	-3.08315500
C	-1.89657500	-1.80433800	-3.99141200
C	-2.13739100	-2.91609000	-3.24385900
C	-2.18377200	-2.49924300	-1.86416700
N	-2.01188300	-1.13447800	-1.79349800
C	-2.43995800	3.35733400	0.53147600
C	-1.70218300	0.65516700	-3.48629300
C	-2.30832200	-3.37669100	-0.77383700
C	-1.73151000	-0.65233900	3.25056100
H	-2.31879100	4.93861100	-1.87789700
H	-1.80715200	3.48254600	-4.04129000
H	-1.80054400	-1.75331900	-5.06586900
H	-2.25232000	-3.92754900	-3.60319100
H	-2.15908900	-4.95223300	1.63100900
H	-1.72422500	-3.47942600	3.80370600
H	-1.91284000	1.75239900	4.82498700
H	-2.40367100	3.91291600	3.35538900
Al	-2.12117400	-0.01833500	-0.09539200
C	-2.70164100	4.83001700	0.81073000
C	-1.43892700	5.69412000	0.99404100
H	-3.33066400	4.92276500	1.70026000
H	-3.30747800	5.24420100	0.00056500
C	-1.75722800	7.17783200	1.21486600
H	-0.86384400	5.31178000	1.84701400
H	-0.78747000	5.57845700	0.11919000
H	-2.32798500	7.55967800	0.35608300
H	-2.41987900	7.28405200	2.08591900
C	-1.45456000	0.93900100	-4.96083800
C	-2.72385200	1.09942900	-5.82011300
H	-0.83663500	0.13962700	-5.37939400
H	-0.84441800	1.84229500	-5.05713800
C	-2.41336900	1.32145500	-7.30529900
H	-3.36084700	0.21529500	-5.69855800
H	-3.31159500	1.94297000	-5.43756800
H	-1.76524600	2.20343700	-7.41590600
H	-1.82957700	0.47093000	-7.68708200
C	-2.59624500	-4.84556200	-1.05124300
C	-1.36180700	-5.74760700	-1.23806000
H	-3.22927200	-4.92266800	-1.93987200
H	-3.21144500	-5.24339700	-0.24003100
C	-1.72973000	-7.22015500	-1.45725400
H	-0.77772600	-5.38596900	-2.09393900
H	-0.70463100	-5.65252200	-0.36549700
H	-2.31011600	-7.58252700	-0.59653400
H	-2.39852200	-7.30435300	-2.32607200
C	-1.44165200	-0.92351900	4.72015800
C	-2.67214600	-1.14838100	5.61833800
H	-0.85369300	-0.09480300	5.12361400
H	-0.78283700	-1.79333800	4.79822200
C	-2.29798900	-1.36085800	7.09032000
H	-3.35499800	-0.29647500	5.52342700
H	-3.23088200	-2.01840300	5.25251300
H	-1.60264400	-2.20949300	7.17235800
H	-1.74413200	-0.48369300	7.45617600
C	-6.31077700	4.12973900	0.72938500
C	-6.08377000	4.17407500	-0.61135300
C	-6.32143300	2.73855400	1.11278500
C	-6.00098200	2.80935500	-1.07378200
N	-6.14578900	1.95773500	-0.00471400
C	-5.99929100	1.11580300	-2.87477000

C	-6.10011500	0.73409900	-4.26196300
C	-6.34812900	-0.60411700	-4.29358400
N	-6.14175000	-0.00214600	-2.08325900
C	-6.35540800	-1.06913400	-2.92797800
C	-6.30193800	-2.87032200	-1.23018300
C	-6.24814000	-4.26050300	-0.84728100
C	-5.96363900	-4.30043900	0.48331300
C	-5.87712700	-2.93456600	0.94149100
N	-6.08008300	-2.08437800	-0.12141900
C	-6.21063800	0.94372300	2.81055300
C	-6.17065700	0.47468600	4.17484600
C	-5.90933100	-0.85922300	4.13222900
C	-5.82346800	-1.23190700	2.73960800
N	-5.98851000	-0.11243800	1.95869400
C	-5.86387100	2.43919000	-2.42274200
C	-6.41729900	2.28299900	2.43869100
C	-5.70606000	-2.55722400	2.28503600
C	-6.48546600	-2.41585500	-2.54822800
H	-6.01392400	5.05648200	-1.23016100
H	-6.43830500	4.96992800	1.39482300
H	-6.31441500	1.08132100	5.05656500
H	-5.81966600	-1.52969200	4.97318900
H	-5.85449400	-5.18262000	1.09666500
H	-6.39238800	-5.10463600	-1.50470200
H	-6.49427500	-1.21664400	-5.17090200
H	-6.02728800	1.40095900	-5.10797200
Al	-6.14504600	-0.04178600	-0.04606400
C	-5.60880400	3.53408000	-3.44928400
C	-6.86298000	4.18490400	-4.06220900
H	-4.99074500	3.12859500	-4.25637800
H	-4.99151800	4.31174300	-2.99065900
C	-6.52487500	5.31603100	-5.04097600
H	-7.45145400	3.41633000	-4.57797400
H	-7.50783300	4.56212800	-3.26052600
H	-5.93949600	6.08733500	-4.51892400
H	-5.86935600	4.92976400	-5.83572600
C	-6.73140200	3.29396800	3.53219300
C	-5.51385200	3.95467100	4.20507500
H	-7.34479000	2.80915300	4.29587600
H	-7.37539600	4.07502100	3.11966400
C	-5.91085700	4.92587600	5.32359500
H	-4.84895500	3.17830100	4.60211900
H	-4.92912700	4.49029200	3.44623100
H	-6.58759800	5.69200900	4.91806200
H	-6.49229900	4.38611100	6.08493100
C	-5.48474600	-3.65679800	3.31335600
C	-6.77317300	-4.26115200	3.90520500
H	-4.86600900	-3.27054700	4.12881100
H	-4.88674000	-4.45422800	2.86297300
C	-6.49740900	-5.39373700	4.90143600
H	-7.34759500	-3.46713600	4.39782400
H	-7.41238600	-4.62514100	3.09229100
H	-5.92800700	-6.19040300	4.40014100
H	-5.84703700	-5.02293700	5.70765100
C	-6.78579100	-3.43955600	-3.63327500
C	-5.54908300	-4.04024400	-4.32918200
H	-7.43646700	-2.98234900	-4.38349700
H	-7.38251000	-4.25114800	-3.20733400
C	-5.91350300	-5.02197200	-5.44940900
H	-4.92677500	-3.23061700	-4.72976600
H	-4.92945700	-4.55137800	-3.58112200
H	-6.54811300	-5.82215400	-5.04152300
H	-6.52882700	-4.50537100	-6.20017100
O	-4.06317100	-0.07567700	-0.26755200
C	-7.76055100	5.96491300	-5.67689000
C	-7.42740900	7.10347300	-6.64864700
H	-8.41794000	6.34626900	-4.88285600
H	-8.34151300	5.19579500	-6.20585300
C	-8.66359600	7.75142100	-7.28427400
H	-6.84691100	7.87384100	-6.12044800
H	-6.76956100	6.72288700	-7.44355100
C	-8.32233500	8.88886400	-8.25152000
H	-9.24279700	6.98248400	-7.81333100
H	-9.32091500	8.13098700	-6.49027400
H	-9.22482500	9.32976400	-8.68743900
H	-7.77344400	9.69005400	-7.74330500
H	-7.69478300	8.53277200	-9.07666300
C	-4.69391600	-5.64635400	-6.13857000

C	-5.05511100	-6.62286200	-7.26446500
H	-4.08243500	-6.16929400	-5.38868800
H	-4.05708100	-4.84639500	-6.54290500
C	-3.83539000	-7.24762000	-7.95291900
H	-5.69204200	-7.42313000	-6.86105200
H	-5.66617400	-6.10140600	-8.01517100
C	-4.20564200	-8.22035900	-9.07656700
H	-3.19905700	-6.44783100	-8.35611100
H	-3.22525700	-7.76981900	-7.20312400
H	-3.31464100	-8.64866600	-9.54748300
H	-4.81282100	-9.05042900	-8.69782200
H	-4.78658700	-7.71901700	-9.85907200
C	-7.76976800	-5.99186200	5.51437100
C	-7.50046300	-7.13375400	6.50184200
H	-8.42320600	-6.35493000	4.70865200
H	-8.33325900	-5.19746800	6.02442300
C	-8.77366600	-7.72970600	7.11491300
H	-6.93778900	-7.92967600	5.99254700
H	-6.84640400	-6.77123800	7.30823300
C	-8.49654400	-8.87047400	8.09860300
H	-9.33518700	-6.93511300	7.62472700
H	-9.42712200	-8.09151900	6.30951100
H	-9.42426100	-9.27345500	8.51795400
H	-7.96711800	-9.69609300	7.60917000
H	-7.87426100	-8.53072400	8.93455400
C	-4.71486400	5.61290500	5.99419100
C	-5.11063000	6.58246300	7.11450400
H	-4.13584700	6.15664900	5.23339700
H	-4.03726900	4.84763600	6.39953700
C	-3.91544800	7.27130300	7.78455200
H	-5.78865600	7.34756700	6.70987300
H	-5.68907500	6.04033400	7.87631200
C	-4.32091400	8.23739600	8.90178500
H	-3.23821900	6.50682400	8.18965000
H	-3.33770600	7.81375700	7.02355600
H	-3.44690700	8.71242100	9.35959100
H	-4.97027800	9.03368300	8.52049300
H	-4.87020200	7.71752700	9.69499300
C	-0.51020900	8.04698500	1.41950200
C	-0.82260200	9.53333000	1.63107800
H	0.15514000	7.93625000	0.55113000
H	0.05580400	7.67008500	2.28410000
C	0.42478400	10.40180500	1.83539400
H	-1.38918200	9.91168200	0.76820300
H	-1.48687900	9.64507700	2.49995400
C	0.10326900	11.88426200	2.04681700
H	0.99148700	10.02353700	2.69732500
H	1.08777800	10.29143100	0.96576500
H	1.01310800	12.47657300	2.18921100
H	-0.43269500	12.30000800	1.18605500
H	-0.52969900	12.03002800	2.92948200
C	-3.66595800	1.50358500	-8.17136400
C	-3.36053200	1.71808900	-9.65876600
H	-4.24571500	2.35814500	-7.79397300
H	-4.31571000	0.62441400	-8.05735000
C	-4.61380700	1.89958100	-10.52385500
H	-2.71122000	2.59817700	-9.77368900
H	-2.78034900	0.86408500	-10.03750700
C	-4.30019700	2.11278700	-12.00773100
H	-5.26213900	1.02032400	-10.40936800
H	-5.19324600	2.75280100	-10.14593300
H	-5.21436200	2.23870700	-12.59690200
H	-3.68249300	3.00566100	-12.15813700
H	-3.75182700	1.25972400	-12.42364000
C	-0.51328900	-8.13071400	-1.66592400
C	-0.87685100	-9.60505600	-1.87977500
H	0.15697000	-8.04480700	-0.79847200
H	0.06388000	-7.77167400	-2.53071300
C	0.33952500	-10.51556000	-2.08790700
H	-1.45474200	-9.96542900	-1.01670900
H	-1.54602000	-9.69204700	-2.74774300
C	-0.03325500	-11.98557900	-2.30192200
H	0.91771500	-10.15520000	-2.94986400
H	1.00721700	-10.43017100	-1.21907700
H	0.85542100	-12.60860300	-2.44680100
H	-0.58209300	-12.38440500	-1.44126200
H	-0.67203900	-12.10756700	-3.18401100
C	-3.50720800	-1.60984700	8.00027400

C	-3.13643500	-1.81634800	9.47390100
H	-4.05715600	-2.49054800	7.63865900
H	-4.20390100	-0.76385800	7.91648000
C	-4.34563900	-2.06687300	10.38332100
H	-2.43895500	-2.66242500	9.55835900
H	-2.58709800	-0.93566700	9.83725700
C	-3.96646700	-2.27141600	11.85304700
H	-5.04217700	-1.22173900	10.29918900
H	-4.89411500	-2.94688300	10.02092800
H	-4.85037100	-2.44756400	12.47463400
H	-3.29838900	-3.13190800	11.97415600
H	-3.44770100	-1.39331300	12.25458200
O	-8.07152200	-0.19606100	0.06410700
H	-8.13988900	-1.11497200	0.35977800
H	-4.11890000	-0.31595700	-1.20316100
C	-10.07928200	3.20335300	-2.81127100
C	-10.26883400	3.92550600	-1.67531700
C	-9.92736500	1.81961900	-2.41429400
C	-10.20228900	2.99987400	-0.56650600
N	-9.96944900	1.73412300	-1.04572200
C	-10.24966200	2.46517700	1.85738100
C	-10.31628100	2.84529100	3.25106600
C	-10.10616400	1.71787900	3.98224400
N	-10.02224300	1.11589400	1.76637700
C	-9.95636000	0.62573500	3.04372100
C	-9.99965200	-1.80442500	2.51971400
C	-10.19641200	-3.18082200	2.91913400
C	-10.43887600	-3.89591100	1.78775300
C	-10.35901400	-2.97463200	0.67661500
N	-10.07274400	-1.71548000	1.15050000
C	-10.03307600	-0.60838000	-2.94211500
C	-10.20585200	-1.69943500	-3.87522300
C	-10.44813000	-2.81976000	-3.14118700
C	-10.38884700	-2.43696400	-1.74880600
N	-10.13441900	-1.08884100	-1.65918900
C	-10.38409300	3.36091000	0.78073700
C	-9.89681100	0.74152600	-3.31889200
C	-10.54644200	-3.33020500	-0.67102800
C	-9.88478600	-0.72950400	3.42021300
H	-10.43435700	4.99067100	-1.60525400
H	-10.08219500	3.57802400	-3.82368900
H	-10.17750900	-1.62574200	-4.95251100
H	-10.63267900	-3.81459700	-3.51977600
H	-10.64616100	-4.95414700	1.72377600
H	-10.18817600	-3.55696100	3.93131400
H	-10.09291900	1.63755700	5.05932200
H	-10.48332100	3.84268200	3.63103700
Al	-10.12687400	0.04290000	0.05479900
C	-10.77489600	4.80116100	1.08800300
C	-9.60273400	5.77778600	1.30373000
H	-11.41749600	4.81870100	1.97334000
H	-11.40357100	5.18083000	0.27694100
C	-10.06311400	7.22087600	1.54302700
H	-9.00650700	5.43825100	2.16065900
H	-8.93330800	5.73762400	0.43579600
H	-10.65267600	7.56273500	0.67999400
H	-10.74784600	7.24777600	2.40309900
C	-9.85829400	1.05994700	-4.80660500
C	-11.24780500	1.21969500	-5.45772200
H	-9.30075700	0.27928100	-5.33264700
H	-9.28313400	1.97595600	-4.97016600
C	-11.17631700	1.54744400	-6.95400000
H	-11.82281400	0.29722300	-5.30807400
H	-11.80101000	2.00822000	-4.93212300
H	-10.59509700	2.47027400	-7.09816700
H	-10.61689400	0.75644900	-7.47497400
C	-10.93079300	-4.77057800	-0.98262000
C	-9.74460100	-5.73063200	-1.19910800
H	-11.57110000	-4.79271900	-1.86987100
H	-11.55628300	-5.15971800	-0.17351300
C	-10.18339600	-7.17830500	-1.45008900
H	-9.14852900	-5.37502700	-2.04940800
H	-9.08001200	-5.68637800	-0.32750400
H	-10.77256100	-7.53414700	-0.59241600
H	-10.86321500	-7.20938400	-2.31401800
C	-9.82060700	-1.05057700	4.90668100
C	-11.19797300	-1.16373700	5.59253900
H	-9.22413900	-0.28912700	5.41841800

H	-9.27212100	-1.98603900	5.05557300
C	-11.10022000	-1.49078800	7.08746600
H	-11.74646500	-0.22392800	5.45342600
H	-11.78975000	-1.93400500	5.08249500
H	-10.54539700	-2.43145100	7.22048800
H	-10.50355300	-0.71703200	7.59286500
C	-8.90838800	8.20054200	1.78617400
C	-9.36536000	9.64523100	2.02225300
H	-8.22245800	8.17300300	0.92726500
H	-8.32150800	7.86073000	2.65200200
C	-8.21093400	10.62421900	2.26892300
H	-9.95053000	9.98717700	1.15644600
H	-10.05267100	9.67331000	2.88003300
C	-8.67680000	12.06436700	2.50330300
H	-7.62674000	10.28318700	3.13466200
H	-7.52425500	10.59665900	1.41172000
H	-7.83075900	12.73780900	2.67624100
H	-9.23407000	12.44581100	1.64005200
H	-9.33739900	12.12996100	3.37539900
C	-12.46480700	-1.60767600	7.77774300
C	-12.37282400	-1.93376100	9.27334500
H	-13.06008300	-2.38187600	7.27331000
H	-13.01915900	-0.66793600	7.64445200
C	-13.73790300	-2.05139000	9.96235200
H	-11.81817300	-2.87390400	9.40760300
H	-11.77845300	-1.15882600	9.77901900
C	-13.63782500	-2.37654100	11.45574300
H	-14.29203800	-1.11241100	9.82854100
H	-14.33136700	-2.82610900	9.45809900
H	-14.62768300	-2.45366000	11.91767300
H	-13.11946200	-3.32824800	11.62030500
H	-13.07978300	-1.60141500	11.99360100
C	-9.01400600	-8.14036900	-1.69347400
C	-9.44991300	-9.58977900	-1.94019800
H	-8.33260900	-8.10825900	-0.83120900
H	-8.42805000	-7.78731900	-2.55461000
C	-8.28103900	-10.55138000	-2.18719900
H	-10.03426000	-9.94484500	-1.07912700
H	-10.13285000	-9.62225100	-2.80134000
C	-8.72606000	-11.99634100	-2.43219200
H	-7.69757700	-10.19727300	-3.04817400
H	-7.59879400	-10.51950200	-1.32663200
H	-7.87007400	-12.65708700	-2.60510000
H	-9.28198700	-12.39048300	-1.57379800
H	-9.38166900	-12.06582100	-3.30775200
C	-12.55244700	1.70989100	-7.61133300
C	-12.48508500	2.03800400	-9.10780500
H	-13.11074900	2.50096100	-7.09102300
H	-13.13316100	0.78767600	-7.46773900
C	-13.86132600	2.20107400	-9.76460100
H	-11.90424100	2.96061100	-9.25228000
H	-11.92738400	1.24645300	-9.62924400
C	-13.78535300	2.52825000	-11.25896200
H	-14.44164200	1.27953700	-9.62073400
H	-14.41819400	2.99234200	-9.24455600
H	-14.78263200	2.63815700	-11.69766200
H	-13.24114800	3.46374500	-11.43268200
H	-13.26456500	1.73786600	-11.81175500
O	-11.92480000	0.13341500	0.14281200
H	-12.36127800	-0.26271900	-0.61825300
